# Influence of the cation in hypophosphite-mediated catalyst-free reductive amination

**DOI:** 10.3762/bjoc.21.130

**Published:** 2025-08-20

**Authors:** Natalia Lebedeva, Fedor Kliuev, Olesya Zvereva, Klim Biriukov, Evgeniya Podyacheva, Maria Godovikova, Oleg I Afanasyev, Denis Chusov

**Affiliations:** 1 A. N. Nesmeyanov Institute of Organoelement Compounds of Russian Academy of Sciences, INEOS, Vavilova St. 28, Moscow, 119334, Russia; 2 Dmitry Mendeleev University of Chemical Technology of Russia, Miusskaya sq. 9, Moscow, 125047, Russia; 3 National Research University Higher School of Economics, Myasnitskaya St. 20, Moscow, 101000, Russia

**Keywords:** amines, DFT, hypophosphites, reductive amination, role of cations

## Abstract

Reducing agents with phosphorus–hydrogen bond, such as sodium hypophosphite, phosphite, and hypophosphorous acid are commercially available in bulk amounts, however, their usage is understudied in organic processes. While NaH_2_PO_2_ has proved to be an efficient four-electron reductant in the catalyst-free reductive amination, the influence of cation in hypophosphite salt has not been studied yet. This issue is a fundamentally important factor. In the present work, the reactivity of the hypophosphites of alkali metals (Li, K, Rb, and Cs) in reductive amination was explored for the first time. A set of secondary and tertiary amines was synthesized from various types of carbonyl compounds and amines. The remedy for Parkinson’s disease, piribedil, was obtained in high yield. The plausible mechanism of the elaborated process was proposed and supported by DFT calculations.

## Introduction

**Scheme 1:**
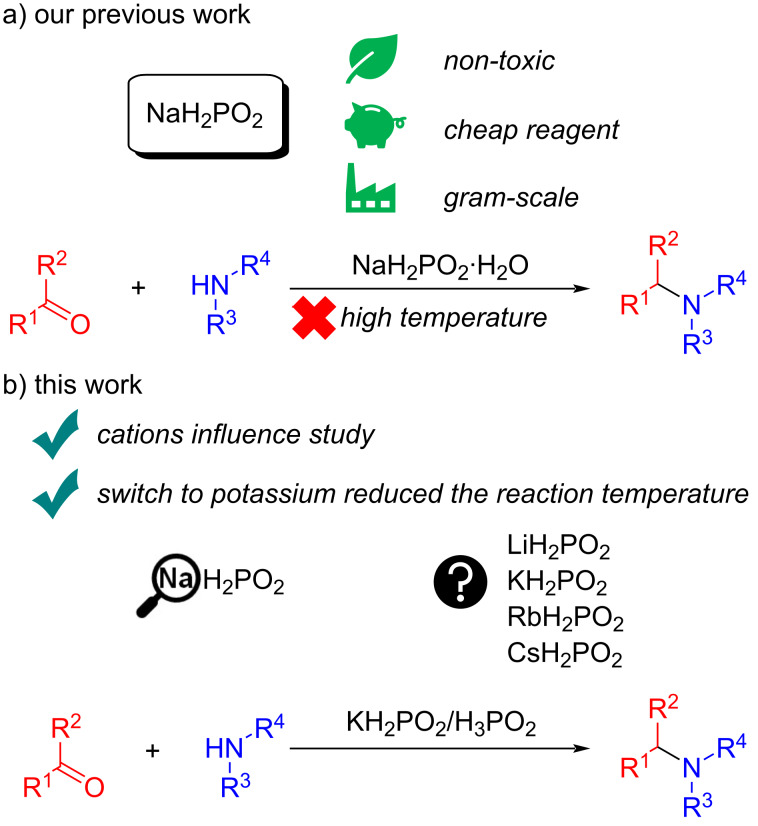
Rationale of the current study: a) Our previous work [20]; b) this work.

Sodium hypophosphite, NaH_2_PO_2_, is one of the most actively applied reductants with phosphorus-hydrogen bond in industry, for example, in production of polymers [1], pharmaceuticals [2], electroless plating [3], metal corrosion prevention [4] and even food preservation [5]. NaH_2_PO_2_ is a non-toxic (LD_50_ 7640 mg/kg – rat) (SDS Thermo Fisher Scientific) and readily available in bulk quantities compound. In organic synthesis, it is most commonly used in metal-catalyzed reductions where NaH_2_PO_2_ serves as a molecular hydrogen donor [6–16]. However, recent studies demonstrated application of hypophosphites as a halogen atom transfer (XAT) agent [17–18]. Standard reduction potentials illustrate that hypophosphite is a powerful four-electron reductant [19]. Our previous studies have proved that NaH_2_PO_2_ can be a selective reducing agent in the catalyst-free reductive amination process [20–22] that can impart an important role in medicinal and pharmaceutical chemistry [23–25] (Scheme 1a). Sodium hypophosphite exhibited good chemoselectivity – it selectively reduced imines while leaving other functional groups intact, e.g., nitro (NO₂), cyano (CN), alkene (C=C), and benzyloxy (OBn) groups. In contrast, usage of classical reducing agents – H_2_ on Pd/C or NaBH_4_ did not show similar chemoselectivity [26].

Additionally, the NaH_2_PO_2_ usability is engaging due to fine green chemistry metrics, e.g., an E-factor less than 1 was reached, moreover the main wastes of this process were safe and useful as fertilizers in agrochemistry phosphates [20].

Multiple literature reports indicate that changing the alkali metal cations can strongly affect diverse chemical processes including radical reactions [27], electrochemical processes [28], and biomass pyrolysis [29]. However, hypophosphites derived from alkali metals other than sodium have been severely understudied in reductive transformations. While at least the structure of LiH_2_PO_2_ is known [30–31], rubidium hypophosphite is not described in the literature. There is only a very limited number of KH_2_PO_2_ utilization examples in copolymerization [32] and synthesis of esters of phosphonous or alkylphosphinic acids [33–35]. Only a single application of cesium hypophosphite was shown in the literature. CsH_2_PO_2_ was prepared in situ and used for formation C–P bond by radical addition to unsaturated carboxylic acids [36]. To summarize the above, it is crucial to fundamentally study the influence of the cations in hypophosphites on this process. In this work, the hypophosphites of Li, K, Rb, and Cs were obtained in situ, the influence of alkali metal cations on the efficiency of reductive amination was assessed and the obtained results were compared with the approach where sodium hypophosphite was used as a reducing agent. The reactivity of LiH_2_PO_2_, KH_2_PO_2_, RbH_2_PO_2_, and CsH_2_PO_2_ in reductive amination was investigated for the first time (Scheme 1b).

## Results and Discussion

**Scheme 2:**
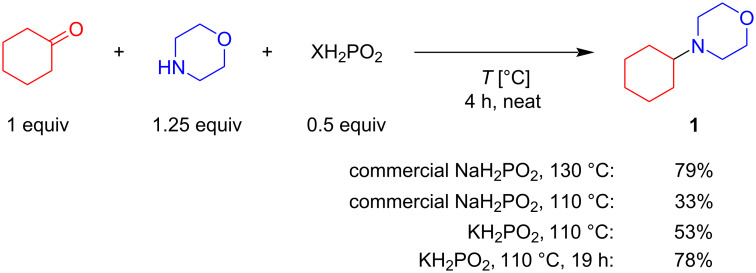
Comparison of KH_2_PO_2_ and NaH_2_PO_2_ under the optimal conditions.

At the initial step, optimization of reductive amination conditions on the benchmark reaction between cyclohexanone and morpholine was carried out (full optimization details are provided in Supporting Information File 1). The reaction could proceed in the presence of only H_3_PO_2_ furnishing the model product in 70% yield (Table 1, line 1) at 130 °C, for 4 h. To conduct the investigation of cation influence on the efficiency of reductive amination, a commercially available NaH_2_PO_2_, and in situ synthesized LiH_2_PO_2_, NaH_2_PO_2_, KH_2_PO_2_, RbH_2_PO_2_, and CsH_2_PO_2_ were compared. To account for the reactivity of H_3_PO_2_ as is, the reaction outcome both for the neutral XH_2_PO_2_ (where X is Li, Na, K, Rb, Cs) and for the 1:1 mixture of XH_2_PO_2_ and H_3_PO_2_ was measured (Table 1). Hypophosphites were generated by reaction between H_3_PO_2_ and the corresponding hydroxide or carbonate. To minimize the contribution of H_2_O from the starting materials, the in situ generated hypophosphites were dried so that the water content in the reaction medium did not exceed 0.7 equiv. In case of LiH_2_PO_2_ or NaH_2_PO_2_ the model amine formed with similar high yields of the target product (65–70%) (Table 1, lines 2 and 3) both in the presence and in the absence of H_3_PO_2_. On the contrary, Rb, K and Cs in the absence of additional H_3_PO_2_ showed lower efficiency at 130 °C, 4 h (3–55%) (Table 1, lines 4–6). However, the reactivity of the mixture of XH_2_PO_2_ and H_3_PO_2_ (at the same total H_2_PO_2_^−^ loading) demonstrated an increased reaction yield. K_2_CO_3_ was cheaper and more available base than RbOH and Cs_2_CO_3_, hence it was chosen for the further study. Using the optimal K_2_CO_3_/H_3_PO_2_ ratio 0.125/0.5, it was found that 78% yield of the model amine could be reached at lower temperature (110 °C) under prolonged reaction time (Scheme 2).

**Table 1:** Cation influence in acidic and neutral conditions.^a^

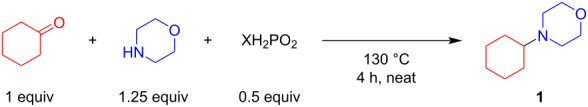						

Line No	Cation in the weakly acidic (pH 3.2)^b^ reductive system^c^ XH_2_PO_2_	Yield of **1**, %		Cation in the strongly acidic (pH 1.6)^b^ reductive system^d^ XH_2_PO_2_ + H_3_PO_2_ (1:1)	Yield of **1**, %	

1	H	70	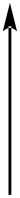			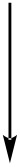
2	Li	68		Li	70	
3	Na	69		Na	65	
4	K	55		K	84	
5	Rb	15		Rb	80	
6	Cs	3		Cs	85	

^a^Reaction conditions: carbonyl compound (1 equiv), amine (1.25 equiv), neat, 130 °C. ^b^pH of corresponding water solutions (0.005–0.1 M) prior to the addition of reagents. For the all details see Table S6 in Supporting Information File 1. ^c^XH_2_PO_2_ was obtained in situ from H_3_PO_2_ (0.5 equiv) and XOH (0.5 equiv) in case Li, Na and Rb or H_3_PO_2_ (0.5 equiv) and X_2_CO_3_ (0.25 equiv) in case K and Cs; ^d^XH_2_PO_2_ was obtained in situ from H_3_PO_2_ (0.5 equiv) and XOH (0.25 equiv) in case Li, Na and Rb or H_3_PO_2_ (0.5 equiv) and X_2_CO_3_ (0.125 equiv) in case K and Cs.

The water content in the system had a crucial influence on the reaction outcome: an excess of the water led to the drop of the yield while comparably low amounts (less than 0.7 equiv) were favorable (see Table S2 in Supporting Information File 1). This influence could be explained by hindering of the iminium ion formation in the presence of water. Thus, the optimization of the reaction conditions has shown clear influence of the cation in the hypophosphite salt on the effectiveness of the reductive amination. The acidity of the reaction media was a key factor affecting the equilibrium in the interaction between carbonyl compounds and amines. Intermediately acidic media is the optimal for the synthesis of imines and enamines [37–38]. In our reducing system H_3_PO_2_ could act as an effective reductant due to its high solubility in neat conditions (Table 1, line 1) (in form of morpholinium hypophosphite). Nonetheless reductive potential of hypophosphite is pH-dependent (−1.65 V at pH 14 vs −0.5 V at pH 0) [19], and usage of an additional amount of base leads to stronger reductive properties. Moreover, the role of the cation could be critical for the thermal stability against disproportionation or aerobic oxidation of hypophosphite [39]; salts with larger cations are also more soluble in organic media. Finally, the combination of H_3_PO_2_ and KH_2_PO_2_ 1:1 with the ratio of H_2_PO_2_^−^ to amine 1:2 is optimal balance between solubility of reductant, acidity of the medium and stability of the reducing system providing the highest efficiency of the interaction.

**Figure 1:**
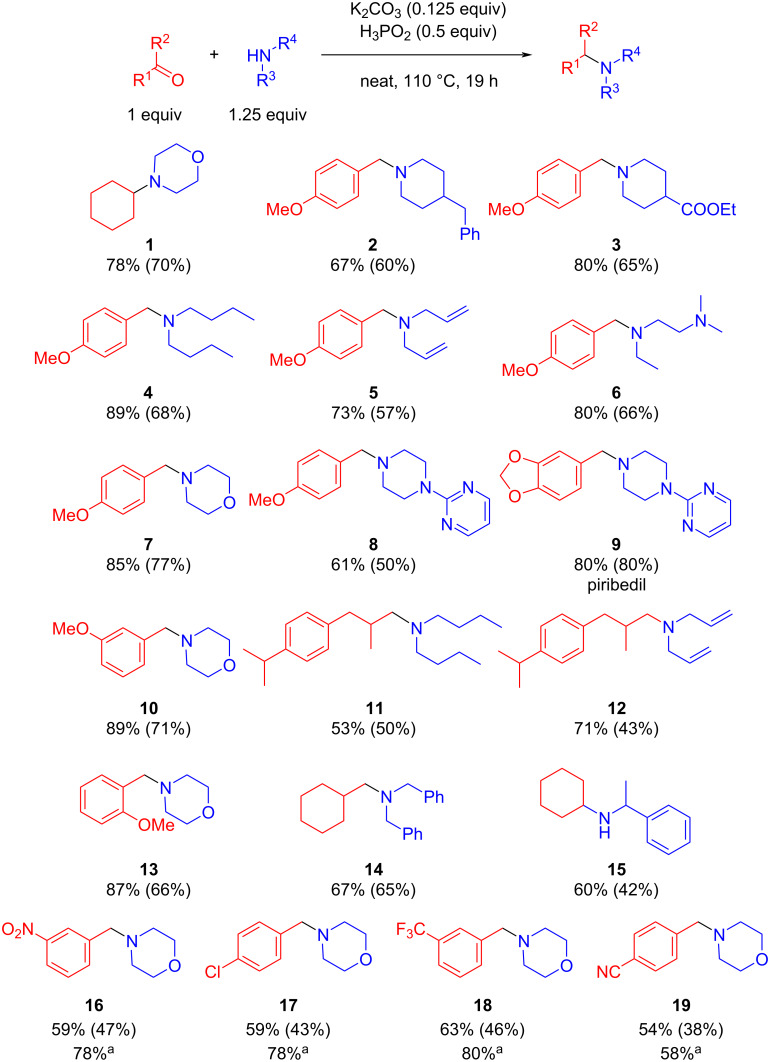
Substrate scope. Reaction conditions: carbonyl compound (1.45 mmol, 1 equiv), amine (1.81 mmol, 1.25 equiv), K_2_CO_3_ (0.181 mmol, 0.125 equiv), H_3_PO_2_ (0.725 mmol, 0.5 equiv.), neat, 110 °C. Yield was determined by NMR, isolated yield in parentheses. ^a^48 h.

Under optimized reaction conditions, the substrate scope of the developed synthetic approach was investigated. Aromatic and aliphatic carbonyl compounds reacted with primary and secondary amines. The reductive amination efficiently proceeded with both cyclic and acyclic secondary amines. Steric hindrance in *ortho*-position in carbonyl compound **13** did not decrease the target product yield. Such functional groups as multiple carbon–carbon bonds (**5**, **12**), COOR (**3**), NO_2_ (**16**), CN (**19**), heteroaromatic moieties (**8**, **9**) remained intact under our reaction conditions. Compounds **1**, **7**, **10**, and **13** were synthesized in the same yields as in the previous work [20] but under milder conditions [20]. However, reaction with benzaldehydes, bearing electron-withdrawing groups (**16**–**19**) was less effective and provided the target product in 54–63% yield. A prolonged reaction time (48 h) resulted in higher reaction yields for these substrates (58–80%). Additionally, the synthesis of the remedy for Parkinson’s disease, piribedil (**9**), in high yield (80%) demonstrated the practical utility of the elaborated synthetic method (Figure 1).

**Scheme 3:**
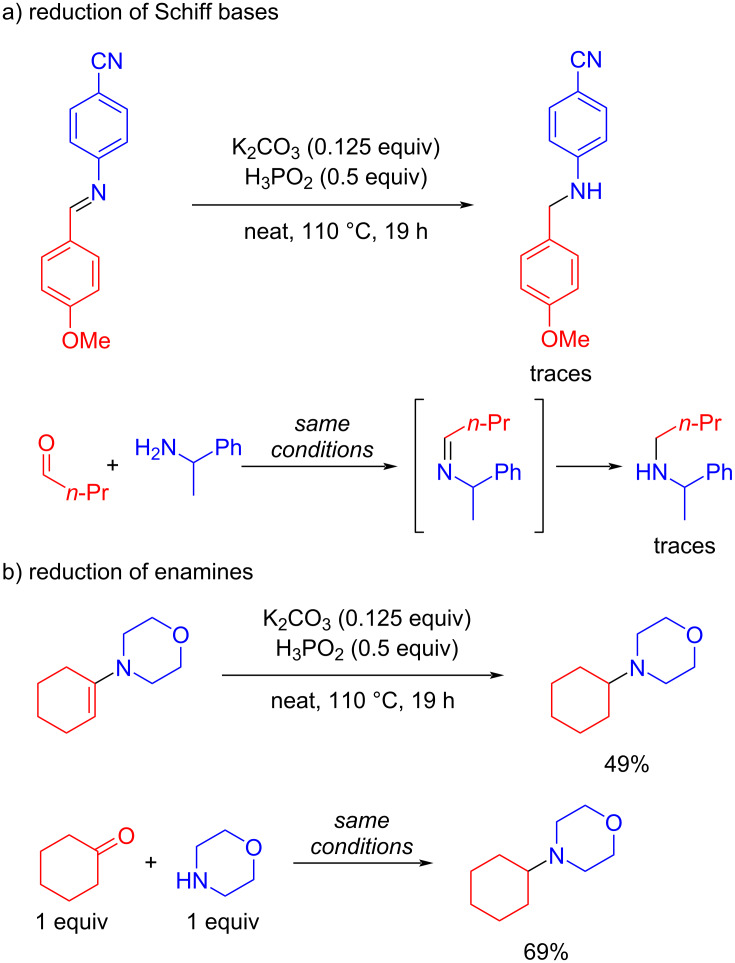
Control experiments.

To get insight into the mechanism of the developed approach the reaction mixtures were thoroughly analyzed and several control experiments were carried out. During the substrate screening we noted that reaction between aldehydes and primary amines resulted in only traces of the product of reductive amination. In these reaction mixtures, exclusively Schiff bases were detected since they precipitated under neat conditions and left the reaction medium, thereby preventing their reduction. This fact and the result of the control experiment where Schiff base was used as a starting material (Scheme 3a) demonstrated that Schiff base was not an intermediate in the developed reaction.

The reactions between carbonyl compounds and secondary amines proceeded forming products in moderate to high yields. In this process enamine can be the intermediate. Therefore, enamine was tested in the control experiment and the corresponding product was obtained in moderate yield (49%). To validate the experiment with enamine the reductive amination with equal amounts of the corresponding carbonyl compound and amine was carried out and the product was obtained in 69% yield (Scheme 3b). Therefore, enamine could be considered as an intermediate or resting state of the reaction, but more likely the real intermediate is an iminium cation or hemiaminal.

**Scheme 4:**
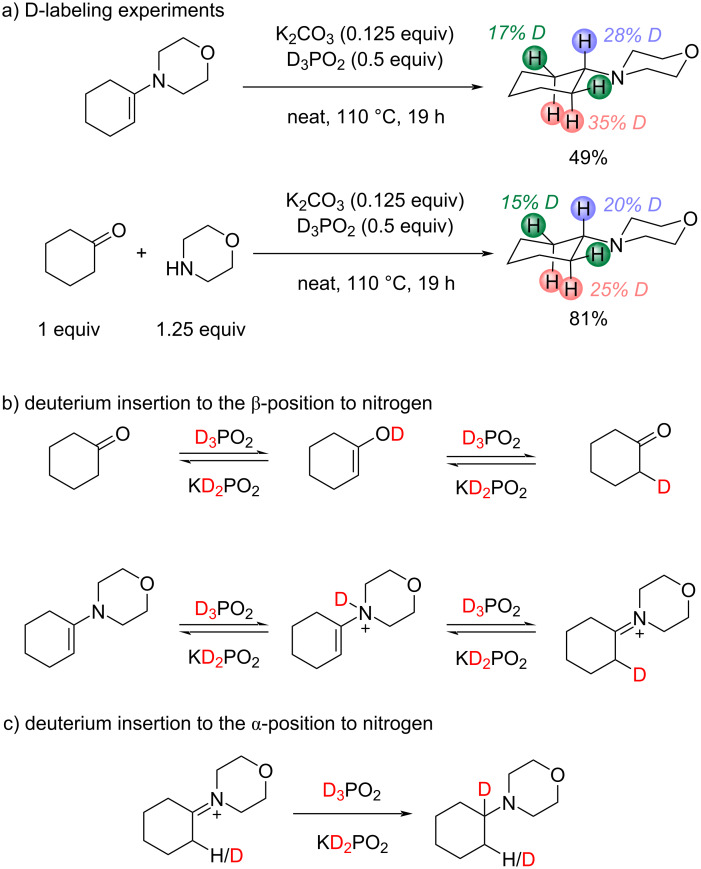
Experiments with D_3_PO_2_.

To check the reduction pathway D-labeling experiments were carried out. The experiments with D_3_PO_2_ illustrated that D-atoms were distributed between α- and β-positions to nitrogen in the product in case of both reduction of enamine and reductive amination of cyclohexanone with morpholine (Scheme 4a). Insertion of D into β-position to nitrogen atom in the product was likely to be caused by fast exchange via keto–enol tautomeric equilibrium in the starting cyclohexanone or equilibrium between iminium cation and enamine (Scheme 4b). Reduction of the iminium cation led to insertion of D into α-position of the target amine (Scheme 4c). The experiments with H_3_PO_2_ and D_3_PO_2_ resulted in the same yield of the amine obtained from enamine under the same reaction conditions. Thus, the kinetic isotope effect (KIE) was not observed in reduction of enamine. Although the experiments with D_3_PO_2_ did not allow us to identify the rate-determining step clearly, they showed the high rate of exchange through tautomeric equilibria compared to the reduction step.

**Scheme 5:**
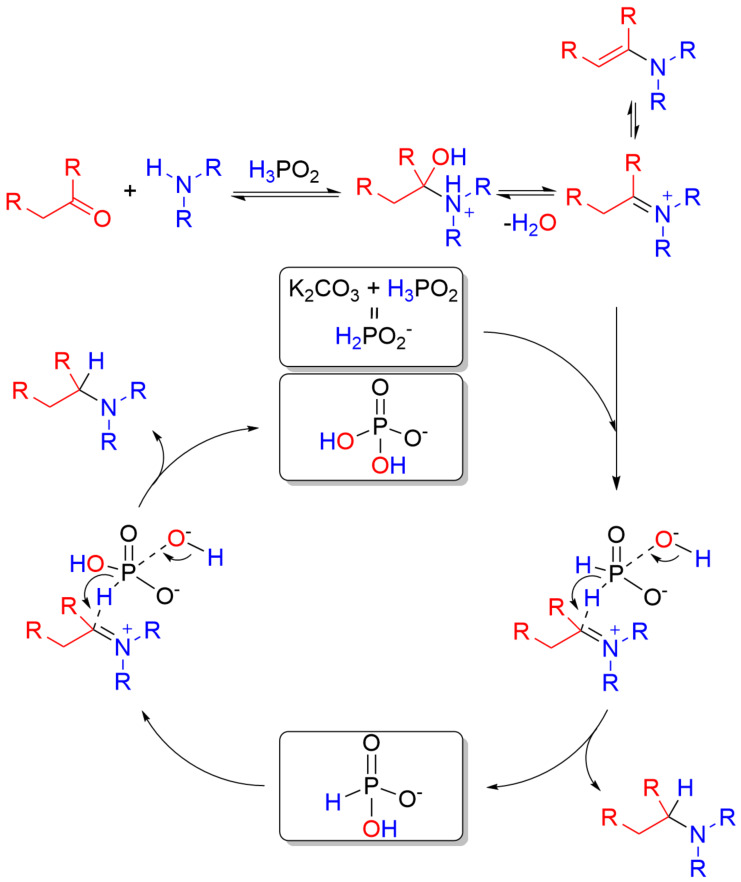
Principal steps of the mechanism of the reductive amination with K_2_CO_3_/H_3_PO_2_ reducing system.

**Figure 2:**
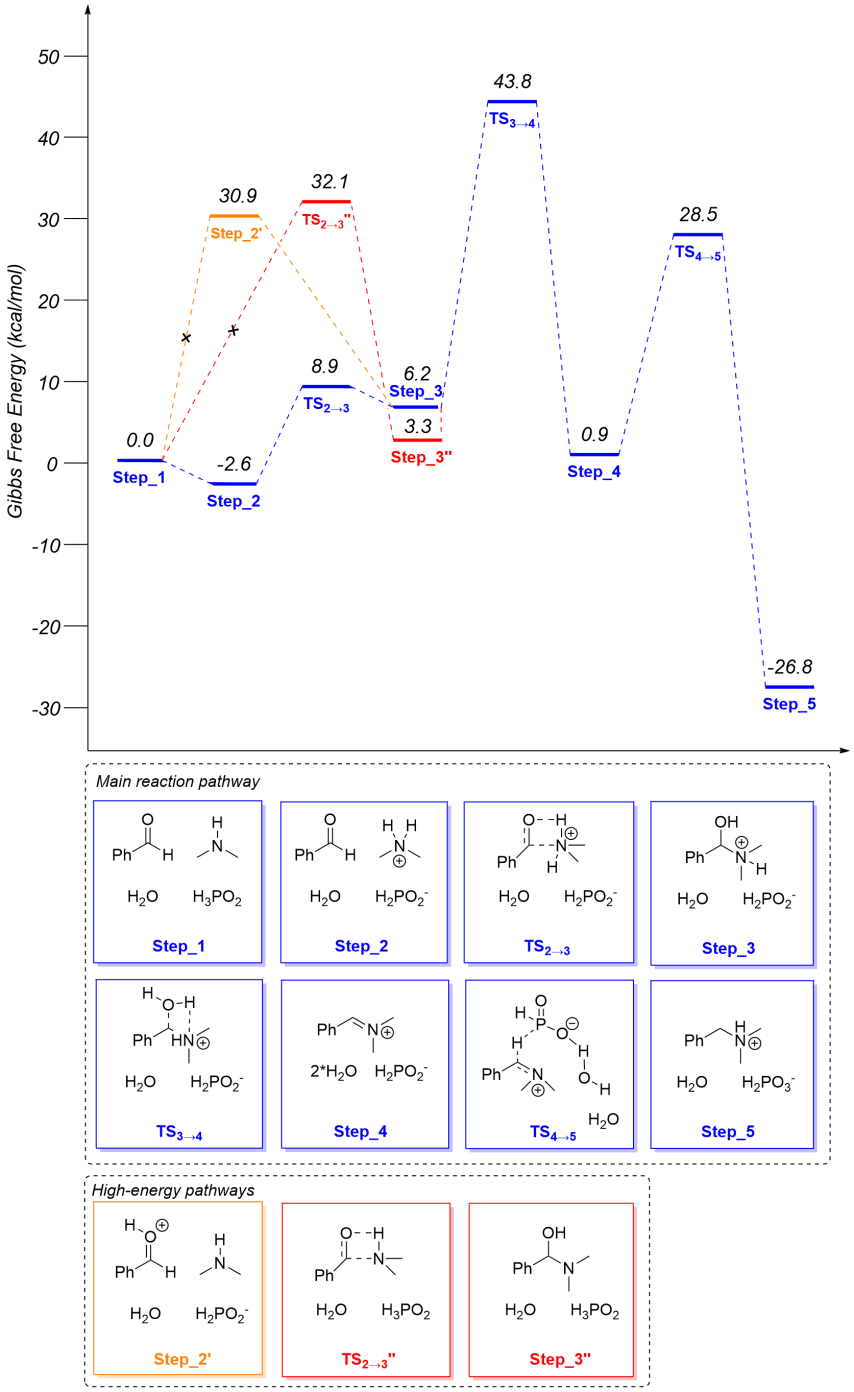
Reaction profile and DFT energies of intermediates and transition states. M062X functional with the basis set 6-311+G(d,p) on the model reaction between dimethylamine and benzaldehyde promoted by hypophosphorous acid.

Based on control experiments and previously obtained data [20], we suggested a plausible mechanism of the developed reaction. Its possibility was supported using the DFT calculations (Scheme 5, Figure 2).

Firstly, reductive amination of an aldehyde started from a nucleophilic addition of the amine to the carbonyl group of the aldehyde. In the presence of acid, this step could occur via acidic catalysis involving a protonation step of an amine (Step_2) or protonation of an aldehyde (Step_2’). Due to the higher basicity of the secondary amine compared with the carbonyl group of benzaldehyde, protonation of dimethylamine was the main reaction pathway (30.9 vs −2.6 kcal/mol). However, it was found that the protonation of the carbonyl group led to a great enhancement of electrophilicity of the reaction center – formation of hemiaminal (Step_3) occurred almost in a barrier-free manner (no TS was found using scan method and it was supported by literature data [40] (2.7 kcal/mol using MP2(full) with a 6-311+G(d,p) basis set)). On the other side, an attack of the weakly nucleophilic secondary ammonium cation to the carbonyl group occurred with Δ*E*_a_ = 11.5 kcal/mol (TS_2→3_). In recent DFT [40] and experimental [41] studies on the reductive amination reaction it was postulated that this protonation of amine played a key role in the catalytic cycle especially in the absence of an external hydrogen source. The alternative pathway to form a hemiaminal could not include the interaction of an acid with amine or aldehyde, nevertheless, the non-catalytic path had Δ*E*_a_ = 32.1 kcal/mol (TS_2→3_'') which meant that hemiaminal definitely emerged faster via the amine protonation step. Next, the formed hemiaminal was forced to exothermically eliminate water molecules to form an iminium cation (Step_4) with Δ*E*_a_ = 43.8 kcal/mol (TS_3→4_). The resulting iminium cation participated in a tautomeric equilibrium between the iminium cation and the corresponding enamine which was confirmed by D-experiments.

The whole sequence of transformations was finished by the reduction of the charged iminium cation with the hypophosphite anion forming *N*,*N*,*N*-dimethylbenzylammonium phosphite in exergonic manner with a total Gibbs free energy gain of −26.8 kcal/mol (Step_5). Noteworthy, the target reduction of iminium cation (Δ*E*_a_ = 27.6 kcal/mol TS_4→5)_ had a lower barrier than the elimination of water from the protonated hemiaminal 30.3 vs 43.8 kcal/mol, respectively, which meant that the rate-determining step in the reductive amination of dimethylamine with benzaldehyde was the formation of iminium cation and not its reduction. H_2_PO_3_^−^ generated as a result of the first step could reduce a new portion of iminium ions in a similar way forming another molecule of the target amine and ortho-phosphoric acid derivatives.

Based on the obtained data we can highlight two main reasons why the developed system is more efficient than the earlier described usage of the pure NaH_2_PO_2_: higher solubility of the potassium, rubidium and cesium salts compared to the sodium and lithium and a proper pH of the reaction medium. Acidic catalysis strongly accelerates the rate of hemiaminal and iminium ion formation [42]. The higher ionic radius of potassium facilitates rapid dissolution of the reducing agent thus increasing the reduction rate. Together these factors allow conducting reductive amination reactions selectively and at lower temperatures.

## Conclusion

In conclusion, the reactivity of hypophosphites of alkali metals, such as Li, K, Rb, and Cs was studied in the reductive amination for the first time. The reactivity was strongly influenced by acidity and the nature of the alkali metal cation: under neutral conditions, the yield decreased from Na to Cs, while acidic conditions with H_3_PO_2_ reversed this trend, enhancing yields with larger cations. This underscores the synergistic role of medium acidity and cation size in optimizing reductive amination. The KH_2_PO_2_/H_3_PO_2_ system allowed us to carry out the process under milder conditions in comparison with NaH_2_PO_2_·H_2_O. Nevertheless, the reaction efficiency falls down for some products, in particular, those containing electron-withdrawing groups. Wide range of amines was synthesized in moderate to good yields. Aliphatic ketones, aliphatic and aromatic aldehydes have successfully reacted with primary and secondary amines. Multiple bonds, heteroaromatic fragments, and COOR groups remain intact under the reaction conditions. The synthesis of an agonist of the dopamine receptor in the brain and a remedy for Parkinson’s disease, piribedil, illustrates the practical utility of the elaborated approach. Control experiments and literature data allowed us to propose a mechanism of the developed reaction. Fast proton exchange between tautomeric forms of carbonyl compounds or iminium cations and enamines was shown in the reaction with D_3_PO_2_. A combination of four-electron reductants KH_2_PO_2_ and H_3_PO_2_ are likely to transfer H-atoms to iminium ion furnishing the target amines. The mechanistic pathway of the developed transformation was calculated and the obtained data supported the role of the presence of hypophosphorous acid in the system.

## Supporting Information

File 1 Optimization details, experimental procedures, calculation details and copies of NMR and HRMS spectra.

## Data Availability

All data that supports the findings of this study is available in the published article and/or the supporting information of this article.
