# Supplementary material for: Influence of the cation in hypophosphite-mediated catalyst-free reductive amination
Source: Beilstein J Org Chem. 2025 Aug 20;21:1661–70. doi: 10.3762/bjoc.21.130 (PMC12415911; doi:10.3762/bjoc.21.130)
Supplement: File 1 — Optimization details, experimental procedures, calculation details and copies of NMR and HRMS spectra. [file Beilstein_J_Org_Chem-21-1661-s001.pdf]

BEILSTEIN JOURNAL OF ORGANIC CHEMISTRY

Supporting Information

for

Influence of the cation in hypophosphite-mediated catalyst-free reductive amination

Natalia Lebedeva, Fedor Kliuev, Olesya Zvereva, Klim Biriukov, Evgeniya Podyacheva, Maria Godovikova, Oleg I. Afanasyev and Denis Chusov

Beilstein J. Org. Chem. **2025,** *21,* 1661–1670. doi:10.3762/bjoc.21.130

Optimization details, experimental procedures, calculation details and copies of NMR and HRMS spectra

License and Terms: This is a supporting information file under the terms of the Creative Commons Attribution License (https://creativecommons.org/licenses/by/4.0). Please note that the reuse, redistribution and reproduction in particular requires that the author(s) and source are credited and that individual graphics may be subject to special legal provisions.
The license is subject to the *Beilstein Journal of Organic Chemistry* terms and conditions: (https://www.beilstein-journals.org/bjoc/terms)

Table of contents

General information..**S2**

Optimization of the reaction conditions..**S4**

Mechanistic studies..**S9**

Computational studies..**S12**

Characterization of products..**S27**

^{1}H and ^{13}C NMR spectra of the products.......................................**S36**

HRMS spectra of the products...**S60**

References..**S67**

General information

Unless otherwise stated, all reagents were purchased from commercial suppliers and used without further purification.

Isolation of products was performed by flash chromatograph InterChim PuriFlash or using column chromatography (Acros Organics, silica gel 0.06–0.200 mm); ethyl acetate/methanol/triethylamine, hexane/ethyl acetate/triethylamine or hexane/ethyl acetate binary and tertiary systems were used as an eluent. All details about physical data and particular chromatographic parameters are provided with the description of each compound.

^{1}H and ^{13}C spectra were recorded in $CDCl_3$ on Bruker Avance 300, Bruker Avance 400, or Varian Inova 400 spectrometers. ^{31}P spectra were recorded in D_2O on Bruker Avance 400. Chemical shifts are reported in parts per million relative to $CHCl_3$ (7.26 and 77.16 ppm for ^{1}H and ^{13}C respectively) or relatively to 85% wt. H_3PO_4 in H_2O (0 ppm). The following abbreviations were used to designate chemical shift multiplicities: s = singlet, d = doublet, dd = doublet of doublets, t = triplet, q = quartet, quint. = quintet, m = multiplet, br = broad, sept = septet; coupling constants are given in Hertz (Hz).

pH was measured with pH-controller Hanna BL 931700 equipped with glass electrode HI1230B with double reference silver/silver chloride electrode and double salt bridge. Calibration of the equipment was carried out with a combination kit of buffer solutions (HI-54710-10) with pH 4.01, 7.01, 10.01 at 25 °C.

Analytical gas chromatography (GC) was performed using a Chromatec Crystal 5000.2 gas chromatograph fitted with a flame ionization detector and a MS detector. Chromatec CR-5MS (30 meters) capillary column were used.

GC settings for the yield determination using FID detector and CR5ms column:

The injector temperature was 250 °C, split ratio of 10:1 at the moment of injection, the FID temperature was 250 °C. Column compartment temperature program: 100 °C for 2 min, 100 °C → 290 °C at 30 °C/min, 290 °C for 3 min. Flow rate 1 mL/min, column CR-5ms.

GC settings for the qualitative analysis using MS detector and CR5-ms column:

The injector temperature was 250 °C, split ratio of 40:1 at the moment of injection. Column compartment temperature program: 60 °C for 2 min, 60 °C → 250 °C at 30 °C/min, 250 °C for 12 min. Flow rate 1.5 mL/min. MSD parameters: ion source temperature 200 °C, transfer line temperature 290 °C. Retention times (r.t.) and integrated ratios were obtained using Chromatec Analytic Software.

High-resolution mass spectra were recorded on a LCMS-9030 device (Shimadzu, Japan) by electrospray ionization mass spectrometry (ESI-MS). Measurements were carried out in positive ion mode; samples were dissolved in acetonitrile and injected into the mass-spectrometer chamber from an HPLC system LC-40 Nexera (Shimadzu, Japan). The following parameters were used: capillary voltage 4.5 kV; mass scanning range: *m/z* 100–1000; external calibration with solution NaI in $MeOH/H_2O$; drying and

heating gases (nitrogen) (each 10 L/min); nebulizing gas (nitrogen) (3 L/min); interface temperature: 300 °C; flow rate acetonitrile 0.4 mL/min. Molecular ions in the spectra were analyzed and matched with the appropriately calculated *m*/*z* and isotopic profiles in the LabSolutions v.5.114 program.

Optimization of the reaction conditions

General procedure

A 12 mL glass tube with a screw-cap wrapped by sealing tape was charged with stirring bar, additive and 50% aqueous solution of hypophosphorous acid H_3PO_2. After CO_2 evolution ceased, the obtained solution was stirred under reduced pressure (0.2 mmHg) at 80 °C for 25 minutes to remove the excess of H_2O so that 5–18 mg (0.2–0.7 equiv) of water remained. Water was not completely removed because anhydrous hypophosphorous acid is unstable. Residual amount of H_2O was calculated as the difference between the mass of hypophosphite or system MH_2PO_2/H_3PO_2 after drying and theoretical mass of the anhydrous hypophosphite or anhydrous system MH_2PO_2/H_3PO_2. Then morpholine and cyclohexanone were added, the tube was sealed and placed into a preheated oil bath where stirring was continued at an indicated temperature. After the indicated time, the reaction mixture was cooled down to the room temperature, washed with 5 mL of DCM and 1 ml of saturated potassium carbonate solution (ca. 50 wt % solution in water), placed into ultrasonic bath for 10 minutes until all solid chunks destroyed and then centrifugated (10 minutes, 8000 rpm). A sample of the resulting solution was analyzed by GC with an external standard.

Table S1. Cation influence

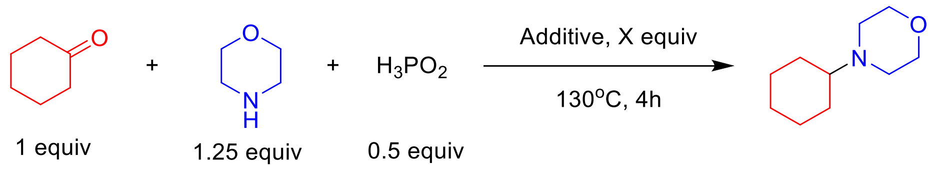

Entry[a]	Additive	Equivalents of additive	Yield, %
**1**	**NaH_2PO_2*H_2O[b]**	**0.5**	**79**
2	none	–	70
3	LiOH	0.5	68
4	LiOH	0.25	70
5	Li_2CO_3	0.125	71
6	Li_2CO_3	0.25	68
7	NaOH	0.25	71
8	NaOH	0.5	69
9	Na_2CO_3	0.125	69
10	Na_2CO_3	0.25	70
11	KOH	0.25	80
12	KOH	0.5	65
13	K_2CO_3	0.25	55
**14**	**K_2CO_3**	**0.125**	**84**
15	RbOH	0.5	15
16	RbOH	0.25	80
17	Cs_2CO_3	0.25	3
**18**	**Cs_2CO_3**	**0.125**	**85**
19	Et_3N	0.5	75
20	Et_3N	0.25	70

[a] 1 equiv, 1.45 mmol, 150 µL of cyclohexanone, 1.25 equiv, 1.81 mmol, 157 µL of morpholine, 0.5 equiv, 0.72 mmol, 78.5 µL of hypophosphorous acid, 0.125–0.5 equiv of additive, 130 °C, 4 h. [b] No H_3PO_2 was added. Yields were determined by GC with an external standard.

Table S2. Influence of the amount of water

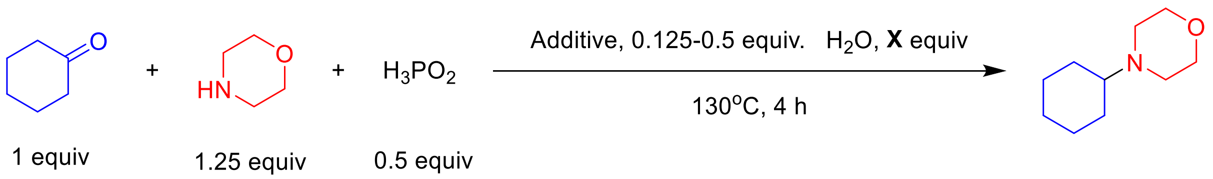

Entry[a]	Additive	Equivalents of additive	Equivalents of H_2O	Yield,%
1	NaH_2PO_2*H_2O[b,c]	0.5	3.0	9
2	NaH_2PO_2*H_2O[b,c]	0.5	2.3	12
3	NaH_2PO_2*H_2O[b,c]	0.5	1.8	15
4	NaH_2PO_2*H_2O[b]	0.5	0.5	79
5	none	-	0.7	70
6	none[d]	-	1.8	50
7	LiOH[d]	0.5	1.8	23
8	LiOH	0.5	0.7	68
9	NaOH[d]	0.5	1.8	52
10	NaOH	0.5	0.7	69
**11**	K_2CO_3*1.3H_2O[d]	0.125	2.3	30
12	**K_2CO_3**	**0.125**	**0.7**	**84**
13	Cs_2CO_3*4.9H_2O[d]	0.125	2.7	7
**14**	**Cs_2CO_3**	**0.125**	**0.7**	**85**

[a] 1 equiv, 1.45 mmol, 150 μL of cyclohexanone, 1.25 equiv, 1.81 mmol, 157 μL of morpholine, 0.5 equiv, 0.72 mmol, 78.5 μL of hypophosphorous acid, 0.125–0.5 equiv of additive, 130 °C, 4 h; [b] No H_3PO_2 was added; [c] H_2O was added; [d] H_2O was not removed. Yields were determined by GC with an external standard.

Table S3. Cation influence at the decreased temperature

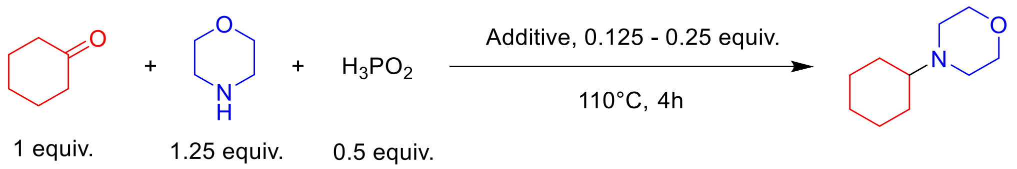

Entry[a]	Additive	Yield, %
1	NaH_2PO_2*H_2O[b]	33
2	RbOH	56
3	Cs_2CO_3	53
4	K_2CO_3	53

[a] 1 equiv, 1.45 mmol, 150 μL of cyclohexanone, 1.25 equiv, 1.81 mmol, 157 μL of morpholine, 0.5 equiv, 0.72 mmol, 78.5 μL of hypophosporous acid, 0.125–0.5 equiv of additive, 130 °C, 4 h. [b] No H_3PO_2 was added. Yields were determined by GC with an external standard.

Table S4. Additive load optimization

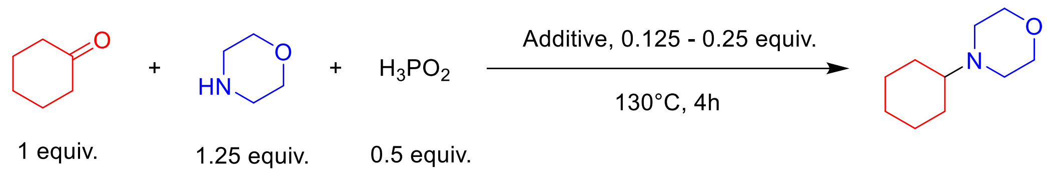

Entry[a]	Additive	Equivalents of additive	Yield, %
1	LiOH	0.5	68
2	LiOH	0.25	70
3	NaOH	0.5	69
4	NaOH	0.25	65
5	RbOH	0.5	15
6	RbOH	0.25	80
7	Cs_2CO_3	0.25	3
**8**	**Cs_2CO_3**	**0.125**	**85**
9	K_2CO_3	0.5	3
10	K_2CO_3	0.375	7
11	K_2CO_3	0.25	55
12	K_2CO_3	0.21875	64
13	K_2CO_3	0.1875	75
**14**	**K_2CO_3**	**0.125**	**84**
15	K_2CO_3	0.09375	76
16	K_2CO_3	0.0625	78
17	K_2CO_3	0.03125	76
18	K_2CO_3	0.015625	75
19	None	-	70

[a] 1 equiv, 1.45 mmol, 150 µL of cyclohexanone, 1.25 equiv, 1.81 mmol, 157 µL of morpholine, 0.5 equiv, 0.72 mmol, 78.5 µL of hypophosphorous acid, 0.125–0.5 equiv of additive, 130 °C, 4 h. Yields were determined by GC with an external standard.

Figure S5. Influence of the KH_2PO_2:H_3PO_2 ratio[a]

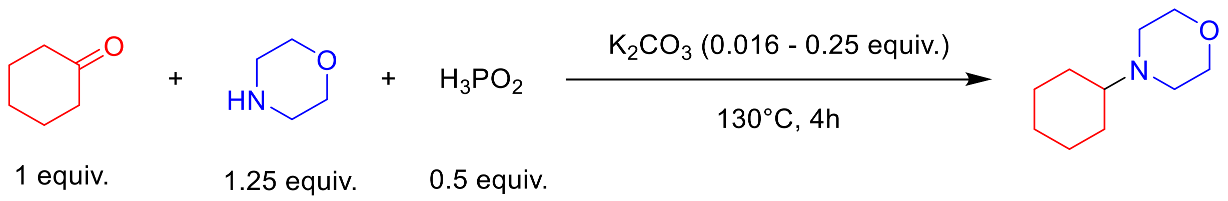

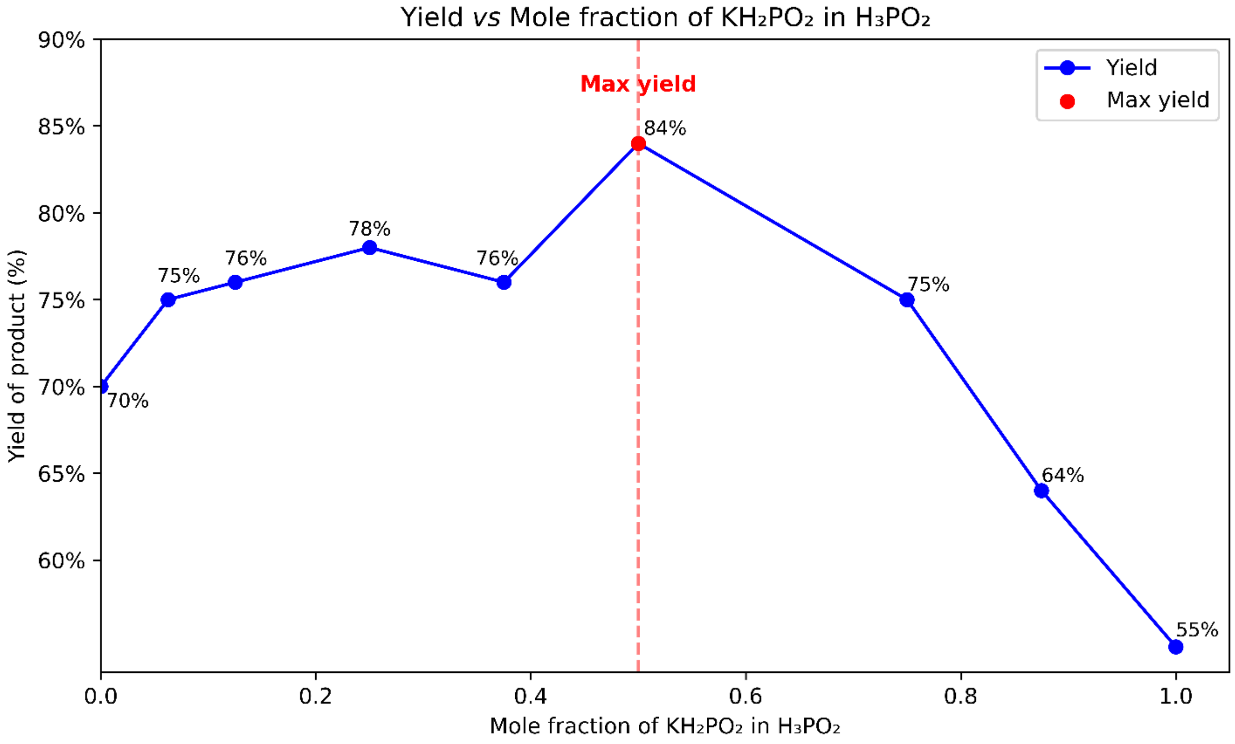

[a] Experimental data from Table S4, Entries 11-19.

Table S5. Reaction time optimization

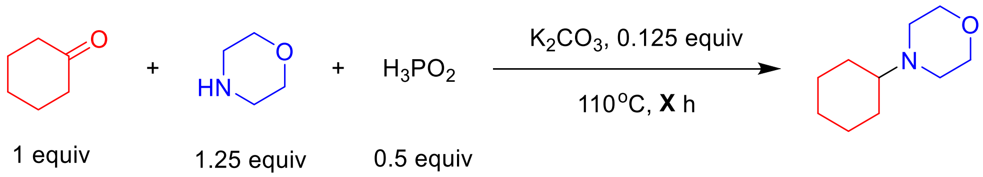

Entry[a]	Time, h	Yield,%
1	4	53
**2**	**19**	**78**

[a] 1 equiv, 1.45 mmol, 150 µL of cyclohexanone, 1.25 equiv, 1.81 mmol, 157 µL of morpholine, 0.5 equiv, 0.72 mmol, 78.5 µL of hypophosphorous acid, 0.125 equiv, 25 mg of potassium carbonate, 110 °C. Yields were determined by GC with external standard.

Table S6. pH measurements

Important notice: valid pH measurement can be conducted in aqueous or non-aqueous solutions for the middle concentrations (0.005–0.1M). We measured pH in water solution remaining the same ratio of reagents as in synthetic experiments. Although this approach did not truly reflect the real process in the reaction medium, it provided correlations for observed trends.

Entry		reagents				pH[a]		
		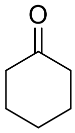	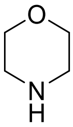	H_3PO_2	K_2CO_3	without reagents	with morpholine	with both reagents
**1**	Equivalents	1	1.25	0.5	0.125	1.64	9.18	9.15
	C in water, M (mol/L)	0.16	0.19	0.08	0.02			
**2**	Equivalents	1	1.25	0.5	0.25	3.16	9.55	9.50
	C in water, M (mol/L)	0.16	0.19	0.08	0.04			
**3**	Equivalents	1	1.25	0.5	0.5	7.77	9.76	9.73
	C in water, M (mol/L)	0.16	0.19	0.08	0.08			

[a] All measurements were taken at least three times until convergence was achieved and then averaged.

Mechanistic studies

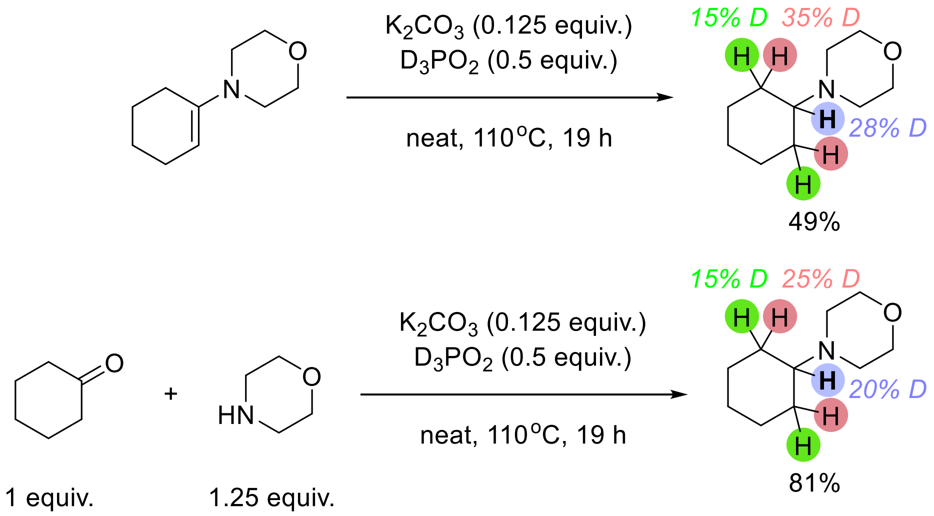

Deuterated hypophosphorous acid (D_3PO_2)

D_3PO_2 was prepared by deuterium-hydrogen exchange according to literature procedure.[1] An H_3PO_2 50 wt % solution (5 mL, 0.097 mol) was dried in vacuo at 80 °C and then stirred in D_2O (4 mL, 99.90%) overnight. This procedure was repeated four times to give a theoretical degree of deuteration of >99% (NMR: >99%).

The spectrum is in agreement with the literature data.[1]

Reduction of enamine. Experiment #1.

A 12 mL glass tube with a screw-cap wrapped by sealing tape was charged with stirring bar, dry potassium carbonate (0.125 equiv, 0.181 mmol, 25 mg) and 50% solution of D_3PO_2 in D_2O (0.5 equiv, 0.725 mmol, 72 µL). After release of CO_2 has ended the obtained homogeneous solution was stirred under reduced pressure (0.2 mmHg) at 80 °C for 25 min. Then 4-(cyclohex-1-en-1-yl)morpholine (1 equiv, 1.45 mmol, 243 mg) was added to the solution, the tube was sealed and placed into a preheated oil bath where stirring was continued at 110 °C. After 19 h, the reaction mixture was cooled down to the room temperature, washed with 5 mL of DCM and 1 ml of saturated potassium carbonate solution (ca. 50 wt % solution in water), placed into ultrasonic bath for 10 minutes until all solid chunks destroyed and then centrifugated (10 minutes, 8000 rpm). 49% yield by GC with an external standard. The residue was purified by column chromatography (eluent: hexane/ethyl acetate 1:3 Rf = 0.3) and analyzed by NMR.

Reductive amination of cyclohexanone with morpholine. Experiment #2.

A 12 mL glass tube with a screw-cap wrapped by sealing tape was charged with stirring bar, dry potassium carbonate (0.125 equiv, 0.181 mmol, 25 mg) and 50% solution of D_3PO_2 in D_2O (0.5 equiv, 0.725 mmol, 72 µL). After release of CO_2 has ended the obtained homogeneous solution was stirred under reduced pressure (0.2 mmHg) at 80 °C for 25 min. Then morpholine (1 equiv, 1.45 mmol, 126 µL) and

cyclohexanone (1 equiv, 1.45 mmol, 150 µL) were added to the solution, the tube was sealed and placed into a preheated oil bath where stirring was continued at 110 °C. After 19 h, the reaction mixture was cooled down to the room temperature, washed with 5 mL of DCM and 1 mL of saturated potassium carbonate solution (ca. 50 wt % solution in water), placed into ultrasonic bath for 10 minutes until all solid chunks destroyed and then centrifugated (10 minutes, 8000 rpm). 80% yield by GC with an external standard. The residue was purified by column chromatography (eluent: hexane:ethyl acetate 1:3 Rf = 0.3) and analyzed by NMR.

Reductive amination of cyclohexanone with morpholine. Experiment #3

A 12 mL glass tube with a screw-cap wrapped by sealing tape was charged with stirring bar, dry potassium carbonate (0.125 equiv, 0.181 mmol, 25 mg) and 50% solution of D_3PO_2 in D_2O (0.5 equiv, 0.725 mmol, 72 µL). After release of CO_2 has ended the obtained homogeneous solution was stirred under reduced pressure (0.2 mmHg) at 80 °C for 25 min. Then morpholine (1.25 equiv, 1.81 mmol, 157 µL) and cyclohexanone (1 equiv, 1.45 mmol, 150 µL) were added to the solution, the tube was sealed and placed into a preheated oil bath where stirring was continued at 110 °C. After 19 h, the reaction mixture was cooled down to the room temperature, washed with 5 mL of DCM and 1 ml of saturated potassium carbonate solution (ca. 50 wt % solution in water), placed into ultrasonic bath for 10 minutes until all solid chunks destroyed and then centrifugated (10 minutes, 8000 rpm). 81% yield by GC with an external standard. The residue was purified by column chromatography (eluent: hexane/ethyl acetate 1:3 Rf = 0.3) and analyzed by NMR.

NMR spectra

^{31}P NMR spectrum of D_3PO_2

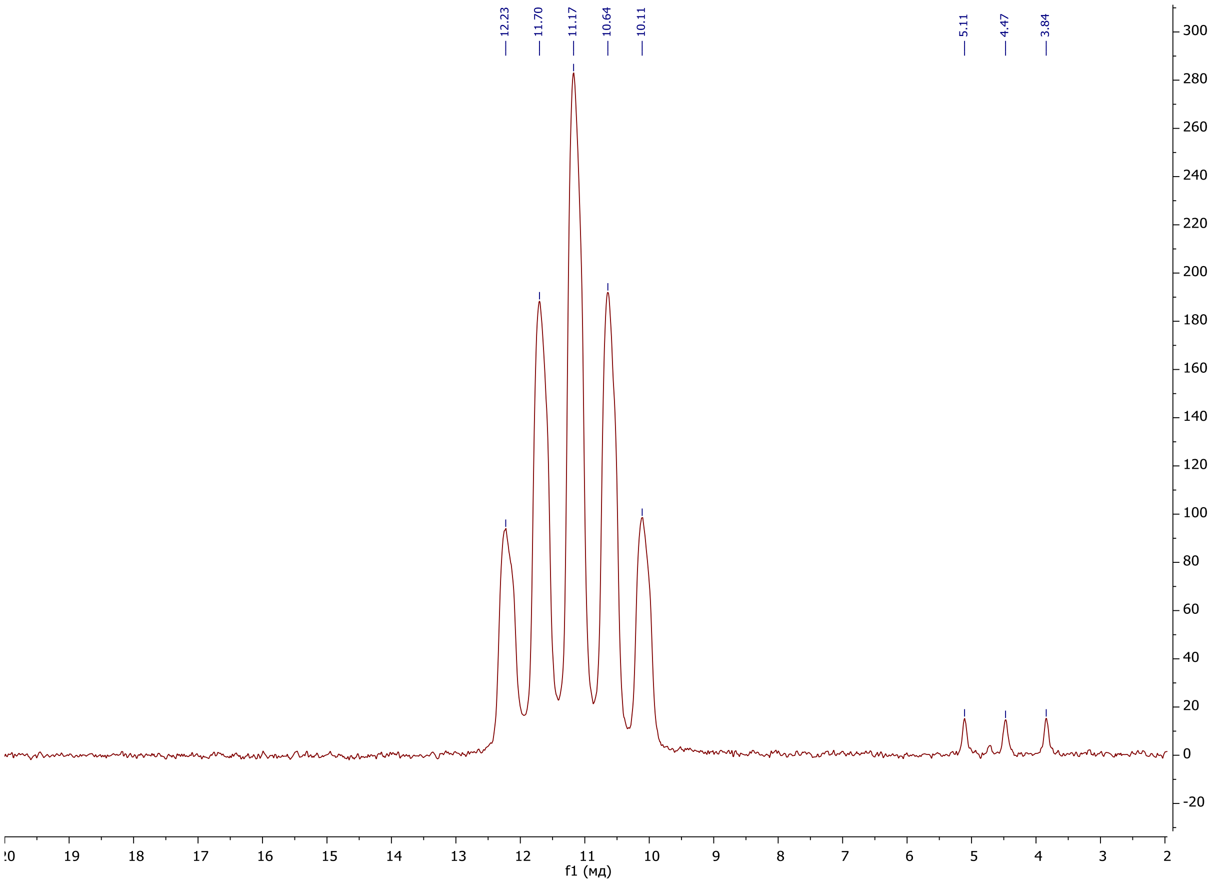

^{1}H NMR spectrum of the product of experiment #1

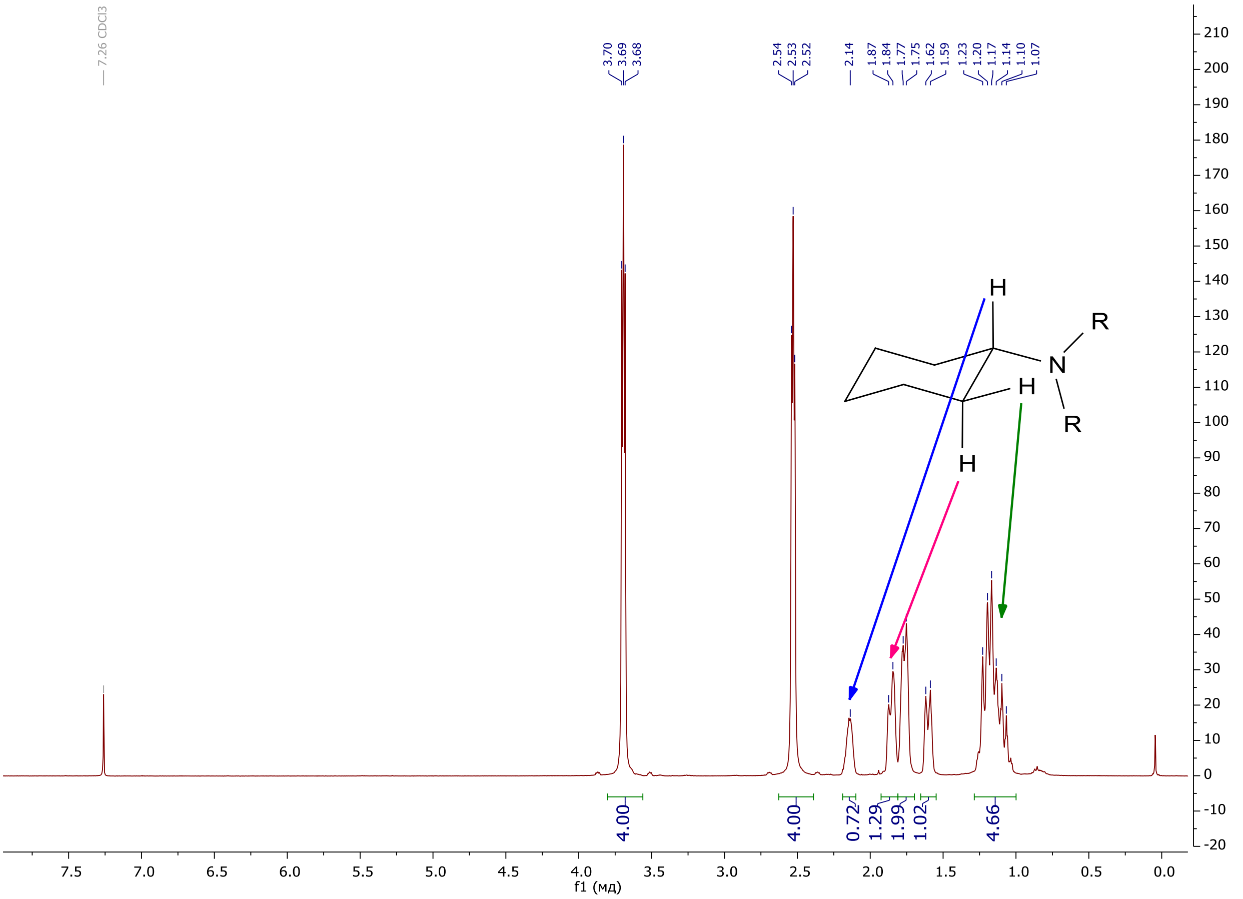

^{2}H NMR spectrum of the product of experiment #1.

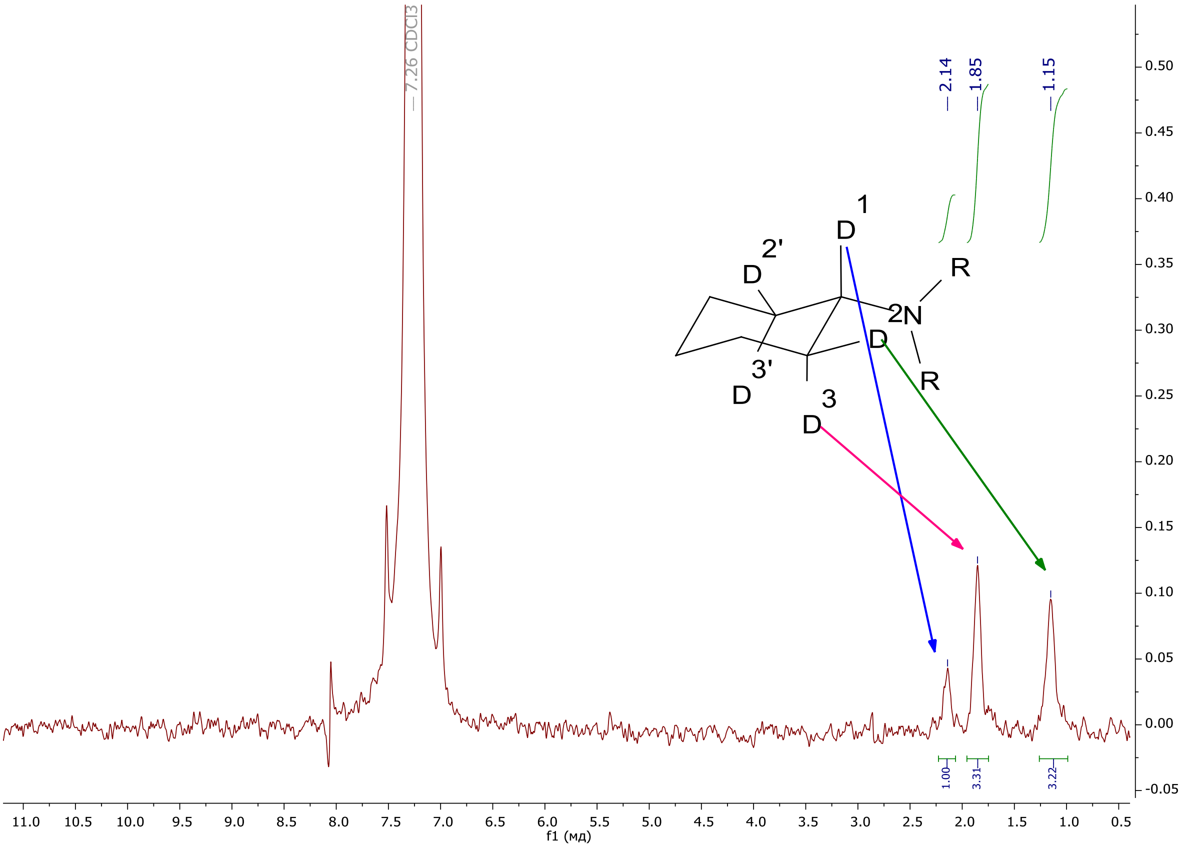

^{1}H NMR spectrum of the product of experiments #2-3.

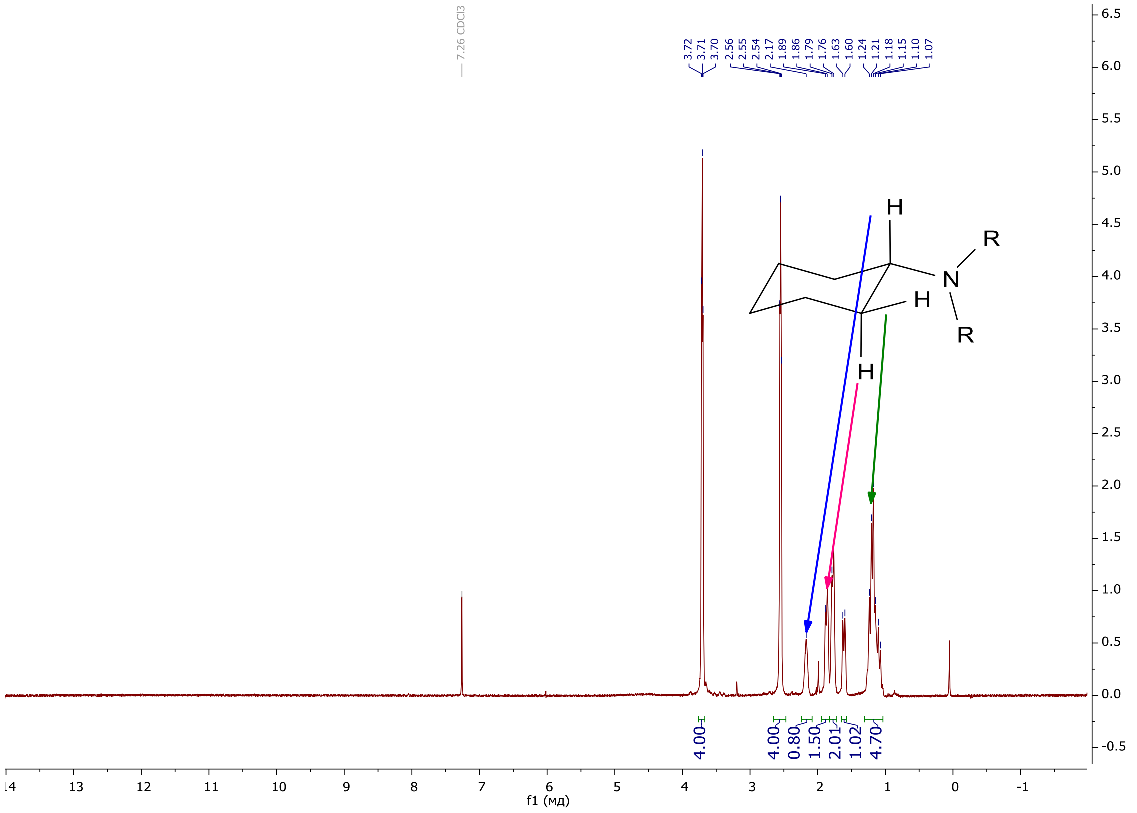

^{2}H NMR spectrum of the product of experiments #2-3.

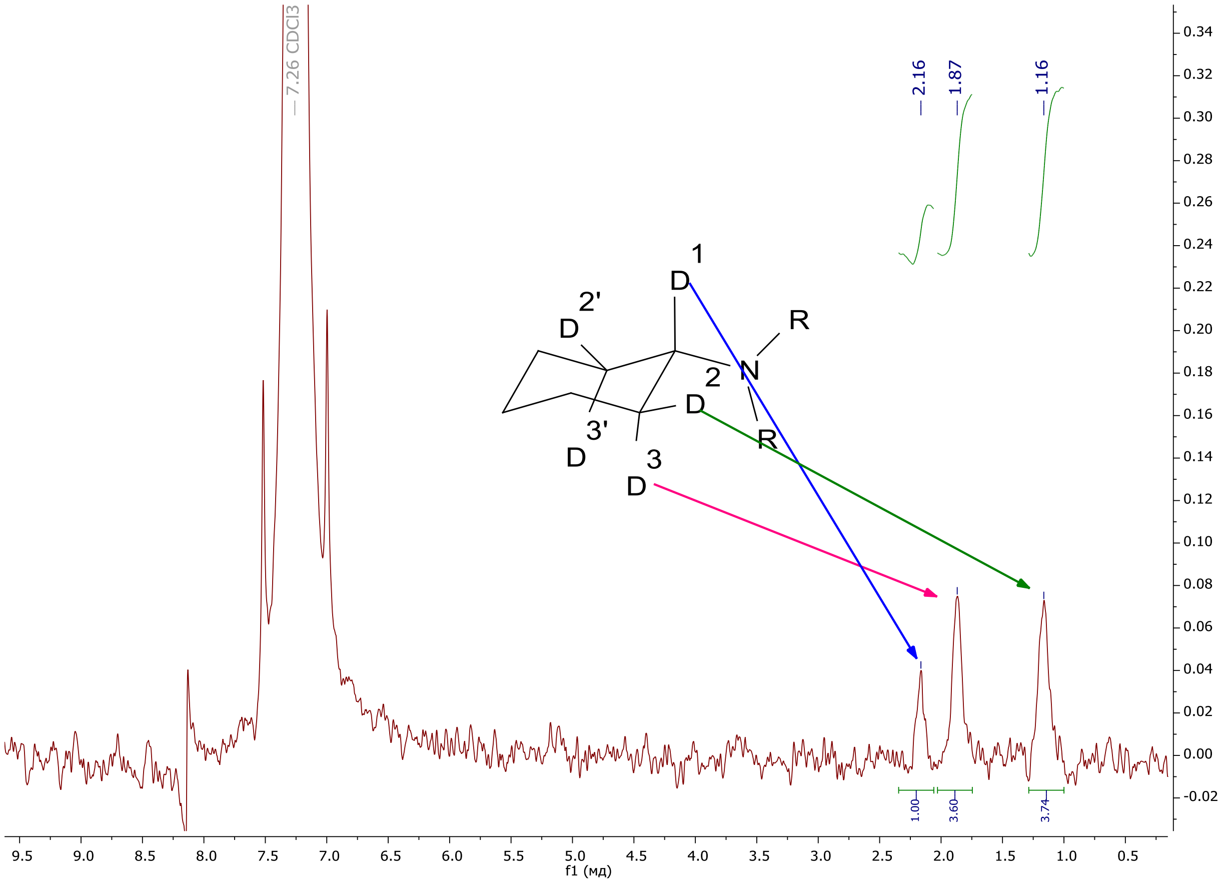

^{1}H-^{13}C HSQC spectrum (400 MHz, $CDCl_3$) of the product of experiments #2-3

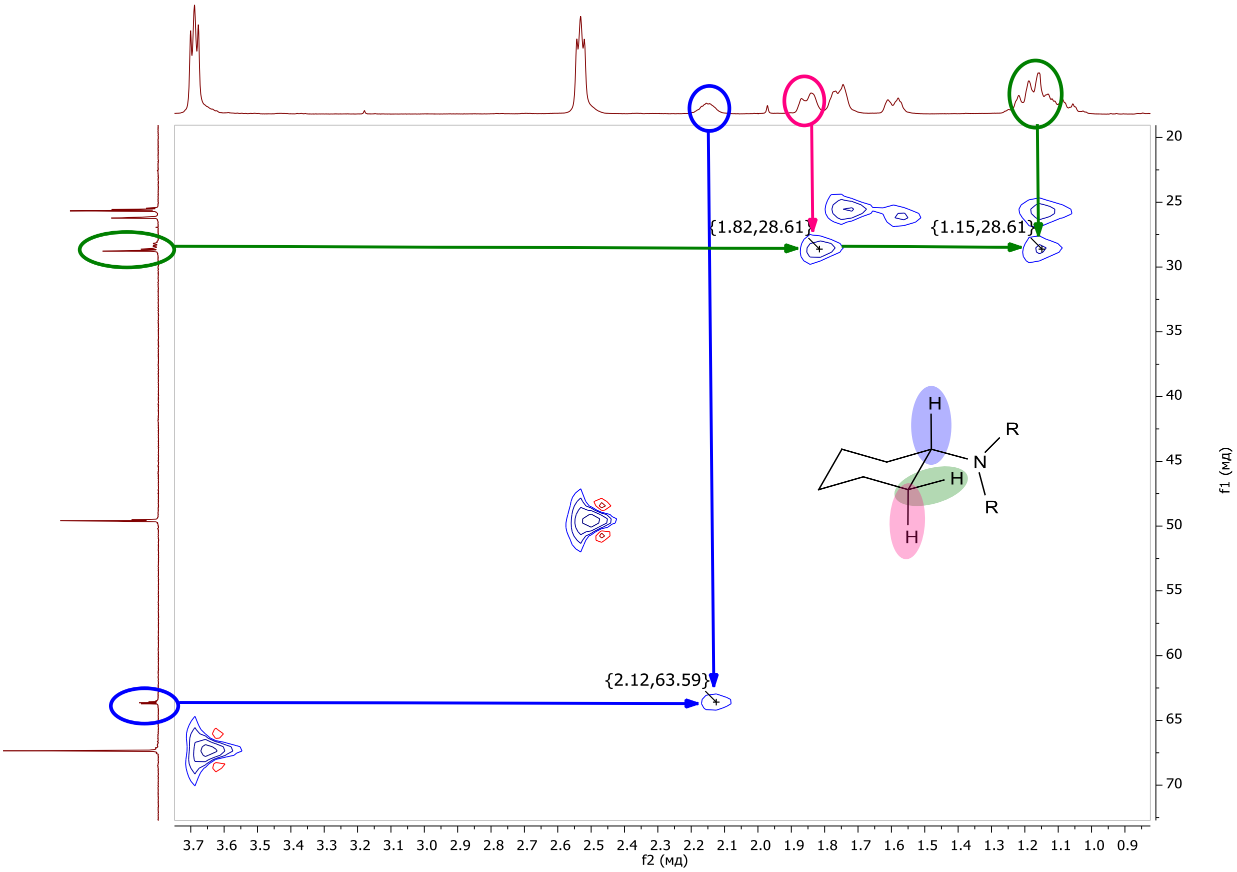

NMR ^{1}H and ^{13}C assignment

According to registered spectra we assigned following signals:

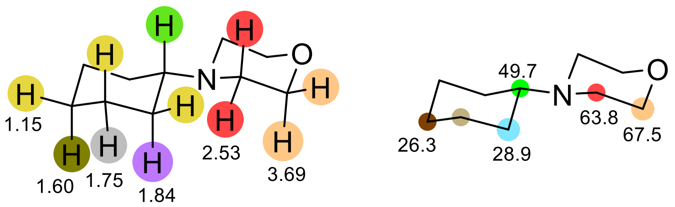

Computational studies

All DFT calculations were carried out with the ORCA 6.0 package[2] and the M062X functional[3] with the basis set 6-311+G(d,p)[4]. All calculations were performed at the standard state (298.15 K, 1 atm) and used the CPCM solvation model in DMSO[5]. All free energies are calculated in liquid phase under standard conditions. Thermodynamic properties were obtained at the same level of theory from a frequency calculation. Minima was characterized by the absence of imaginary frequencies. The Cartesian coordinates (in angstroem) for the calculated structures are given below.

Benzaldehyde

Final Gibbs free energy -345.44654868 Eh

Lowest frequency = 122.14 cm^{-1}

H	0.334000	0.934563	2.763374
C	0.838629	0.713073	1.806546
O	2.038910	0.557513	1.765366
C	-0.055611	0.626721	0.632039
C	-1.425293	0.821584	0.814847
C	0.455105	0.352612	-0.640467
C	-2.288781	0.742989	-0.273445
C	-0.407391	0.274675	-1.724206
C	-1.778228	0.469886	-1.539669
H	-1.808765	1.033034	1.808233
H	1.521682	0.204325	-0.765303
H	-3.352766	0.893244	-0.135722
H	-0.019267	0.063343	-2.713585
H	-2.448812	0.409214	-2.389292

Dimethylamine

Final Gibbs free energy -135.06809929 Eh

Lowest frequency = 226.88 cm^{-1}

N	-1.504227	2.667982	2.163955

C	-1.051795	3.634458	3.160786
H	-1.785547	3.689527	3.968994
H	-0.080597	3.368799	3.603502
H	-0.969741	4.623771	2.708318
C	-1.522773	1.311518	2.704863
H	-0.554972	1.007176	3.129947
H	-2.271851	1.250034	3.498327
H	-1.797590	0.603393	1.921868
H	-0.867558	2.694070	1.374140

H_2O

Final Gibbs free energy -76.42861746 Eh

Lowest frequency = 1588.23 cm^{-1}

O	0.000000	-0.000000	0.119011
H	0.000000	0.759801	-0.471398
H	0.000000	-0.759801	-0.471398

H_3PO_2

Final Gibbs free energy -493.61172483 Eh

Lowest frequency = 308.01 cm^{-1}

H	1.589746	-1.015768	0.824670
H	-1.059524	-0.248558	-0.743208
P	0.136184	0.257507	-0.227770
O	0.767028	-1.116247	0.326392
O	0.972042	1.028326	-1.192131
H	-0.194387	0.985952	0.921858

Dimethylammonium cation

Final Gibbs free energy -135.51637068 Eh

Lowest frequency = 192.17 cm^{-1}

N	-1.475280	2.658644	2.182489
C	-1.056883	3.667167	3.196900
H	-1.785171	3.654593	4.004074
H	-0.073443	3.383464	3.564332
H	-1.023469	4.643973	2.721528
C	-1.539728	1.272411	2.726465
H	-0.546119	1.002228	3.075977
H	-2.249192	1.267262	3.550617
H	-1.864464	0.603701	1.933715
H	-0.826872	2.683350	1.391866
H	-2.388091	2.916733	1.799623

Hypophosphite anion

Final Gibbs free energy -493.16766841 Eh

Lowest frequency = 437.06 cm^{-1}

O	-0.105261	-1.188311	-0.097942
P	-0.533910	0.189046	0.375540
O	-0.058370	1.421090	-0.373364
H	-0.173990	0.326112	1.740055
H	-1.949753	0.220606	0.440467

Benzaldehyde protonated

Final Gibbs free energy -345.84118264 Eh

Lowest frequency = 126.26 cm^{-1}

O	-0.105261	-1.188311	-0.097942
P	-0.533910	0.189046	0.375540
O	-0.058370	1.421090	-0.373364
H	-0.173990	0.326112	1.740055
H	-1.949753	0.220606	0.440467

$TS_{2\rightarrow3}$'

Final Gibbs free energy -480.94446904 Eh

Lowest frequency = -86.06 cm^{-1}

C	0.046978	1.858906	0.958437
O	0.306761	2.614616	0.037509
H	-0.707258	1.096122	0.838883
C	0.787539	2.005838	2.300651
C	0.474458	1.146429	3.354386
C	1.763292	2.993551	2.455898
C	1.149193	1.264871	4.565031
C	2.433421	3.108317	3.665168
C	2.128847	2.242769	4.716631
H	-0.351617	0.445889	3.257049
H	1.900630	3.742960	1.688742
H	0.842103	0.669533	5.414384
H	3.190858	3.868132	3.792647
H	2.654591	2.328487	5.655732
N	1.135863	0.607979	-0.633601
C	1.103234	-0.493937	-1.681397
H	1.724601	-0.158525	-2.522308
H	1.491176	-1.387700	-1.218529
H	0.065865	-0.634480	-1.978367
C	2.554939	0.762246	-0.173222
H	2.645220	1.603596	0.483020
H	2.819303	-0.155304	0.331553
H	3.177060	0.920680	-1.054895
H	1.450336	1.698423	-1.047140

H	0.575962	0.462815	0.187351

TS$_{2\rightarrow3}$''

Final Gibbs free energy -480.46340221 Eh

Lowest frequency = -1592.32 cm^{-1}

N	2.148176	0.954102	-3.588054
H	3.208930	1.440340	-3.159714
C	2.274841	-1.045020	-1.992006
C	2.463960	-0.852997	-0.625599
C	1.395740	-2.036407	-2.431435
C	1.773863	-1.639226	0.294491
C	0.700169	-2.818440	-1.514007
C	0.888967	-2.620283	-0.147314
H	3.156340	-0.088355	-0.293172
H	1.257867	-2.194695	-3.497442
H	1.927833	-1.487369	1.356854
H	0.017033	-3.583848	-1.863936
H	0.352452	-3.231727	0.569227
C	2.999801	-0.193185	-3.007797
O	4.016474	0.612314	-2.540793
H	3.282354	-0.822863	-3.863178
C	0.946862	1.330596	-2.831953
H	0.166347	0.574142	-2.949997
H	1.200373	1.431402	-1.777165
H	0.587998	2.287017	-3.211875
C	1.902132	0.871572	-5.033274
H	1.504353	1.825022	-5.380334
H	2.841363	0.661522	-5.544522

H	1.180315	0.078581	-5.247620

Hemiaminal N-protonated

Final Gibbs free energy -480.94877494 Eh

Lowest frequency = 56.09 cm^{-1}

C	-0.032359	-0.989964	-0.693547
O	0.197488	-1.621529	0.511405
H	-1.096698	-0.856940	-0.898396
C	0.646352	-1.703286	-1.836115
C	0.261462	-1.401645	-3.142134
C	1.649162	-2.641585	-1.601544
C	0.886865	-2.028522	-4.214994
C	2.271286	-3.268948	-2.677069
C	1.893592	-2.962163	-3.981938
H	-0.532874	-0.682224	-3.320439
H	1.935164	-2.881491	-0.584600
H	0.583376	-1.794378	-5.228378
H	3.050011	-4.000647	-2.495914
H	2.379211	-3.455100	-4.815968
H	-0.567528	-1.528251	1.091002
N	0.511507	0.449074	-0.596621
C	1.977133	0.490727	-0.320144
H	2.155814	-0.023746	0.621468
H	2.506242	0.003686	-1.135273
H	2.271927	1.535100	-0.247659
C	-0.246890	1.236824	0.417593
H	-1.308389	1.175035	0.184739
H	-0.039517	0.817679	1.399998

H	0.094652	2.268109	0.370402
H	0.348739	0.882302	-1.511261

Hemiaminal

Final Gibbs free energy -480.50933895 Eh

Lowest frequency = 31.76 cm^{-1}

C	0.313118	-0.691093	-0.738966
O	0.685060	-1.377905	0.454360
H	-0.780691	-0.675347	-0.806503
C	0.859445	-1.465462	-1.924443
C	0.583076	-0.999309	-3.211992
C	1.616112	-2.624046	-1.768836
C	1.060868	-1.678071	-4.327143
C	2.099840	-3.303669	-2.887372
C	1.825202	-2.833770	-4.167199
H	-0.005566	-0.096382	-3.333375
H	1.827976	-2.994107	-0.773854
H	0.838105	-1.306833	-5.321101
H	2.691329	-4.202418	-2.754678
H	2.199613	-3.363659	-5.035634
H	0.124774	-1.066274	1.172493
N	0.744121	0.691679	-0.779450
C	2.192430	0.854100	-0.679973
H	2.583478	0.523884	0.292435
H	2.689059	0.286767	-1.468671
H	2.436207	1.910160	-0.808453
C	0.060236	1.508260	0.218353
H	-1.019218	1.366123	0.132670

H	0.367902	1.268224	1.247671
H	0.287567	2.560528	0.038224

TS$_{3\rightarrow4}$

Final Gibbs free energy -480.88869644 Eh

Lowest frequency = -1385.92 cm^{-1}

N	1.826811	-2.156600	-5.933544
H	0.890958	-1.156184	-5.442074
H	1.111655	-0.226948	-3.907948
C	2.989389	-1.805276	-6.766386
C	1.983441	-3.198613	-3.604304
C	3.156601	-3.931472	-3.423764
C	0.793440	-3.618867	-3.013889
C	3.135568	-5.095020	-2.662082
C	0.780387	-4.778133	-2.244296
C	1.947441	-5.518110	-2.071221
H	4.082787	-3.588597	-3.874453
H	-0.114798	-3.044088	-3.150399
H	4.047213	-5.663184	-2.522085
H	-0.143282	-5.102924	-1.779968
H	1.932690	-6.419944	-1.470135
C	2.058162	-1.990502	-4.482604
O	0.891704	-1.090497	-4.302067
H	2.967195	-1.415480	-4.317113
H	3.779933	-2.552862	-6.652894
H	3.361234	-0.824147	-6.473655
H	2.667275	-1.774139	-7.805756
C	1.215006	-3.431547	-6.346712

H	0.947536	-3.341567	-7.398260
H	1.918288	-4.259086	-6.215882
H	0.314379	-3.619364	-5.764242

N-Methyl-N-(phenylmethylene)methanaminium cation

Final Gibbs free energy -404.52858624 Eh

Lowest frequency = 61.74 cm^{-1}

N	-1.614034	2.073878	0.983285
C	-1.529001	1.652821	-0.423230
H	-1.586480	0.564673	-0.453357
H	-2.377159	2.078625	-0.958679
H	-0.591071	1.995428	-0.851140
C	-2.823850	1.617770	1.681119
H	-3.097561	2.332081	2.453207
H	-3.617210	1.547285	0.940253
H	-2.644482	0.632407	2.112618
C	-0.690222	2.800727	1.505484
H	0.091023	3.123510	0.822074
C	-0.577633	3.263864	2.881713
C	0.103784	4.476674	3.063507
C	-1.038829	2.549906	3.997342
C	0.264005	5.001828	4.337377
C	-0.847280	3.068064	5.270292
C	-0.213194	4.297651	5.440444
H	0.494226	5.006043	2.201184
H	-1.503153	1.579429	3.885158
H	0.771035	5.949433	4.470809
H	-1.188481	2.508448	6.132581

H	-0.077019	4.700262	6.437499

$TS_{4\rightarrow5}$

Final Gibbs free energy -974.08437721 Eh

Lowest frequency = -95.49 cm^{-1}

O	-1.204656	0.381314	0.458356
P	2.185036	2.176252	-0.077154
H	2.608238	2.225142	1.447427
C	2.715136	1.786060	2.671091
N	2.731600	0.437350	2.689879
H	-0.270977	0.496418	0.205140
O	3.426557	2.460230	-0.846918
H	3.705347	2.227082	2.795273
C	1.545308	-0.393061	2.877073
H	1.009732	-0.537374	1.935768
H	1.879382	-1.360599	3.251980
H	0.882000	0.057031	3.612430
C	3.876889	-0.233113	2.085660
H	4.091336	-1.149978	2.635253
H	3.657466	-0.485264	1.042537
H	4.747174	0.421778	2.120209
O	1.455985	0.867997	-0.100832
H	1.303561	3.273427	-0.037554
H	-1.348443	-0.569445	0.463041
C	1.617025	2.557285	3.328609
C	0.289879	2.463737	2.900489
C	1.952270	3.419235	4.373112
C	-0.694545	3.217513	3.528935

C	0.961806	4.164324	5.006704
C	-0.360820	4.064083	4.584776
H	0.021643	1.806386	2.078836
H	2.985785	3.501541	4.691723
H	-1.722158	3.144827	3.192827
H	1.225262	4.824191	5.824823
H	-1.131804	4.648042	5.074005

N,N-Dimethylbenzylamine

Final Gibbs free energy -405.72706697 Eh

Lowest frequency = 64.12 cm^{-1}

N	-1.503141	2.155138	0.601075
C	-0.702499	1.830636	-0.615354
H	-1.173461	0.992010	-1.122842
H	-0.698271	2.711551	-1.255826
H	0.308831	1.575119	-0.307304
C	-2.915900	2.470582	0.242624
H	-2.914155	3.387419	-0.346329
H	-3.311211	1.641016	-0.339108
H	-3.491027	2.602806	1.155461
C	-0.846185	3.271060	1.377669
H	-0.825667	4.131163	0.706987
C	-1.573461	3.583166	2.656012
C	-2.365100	4.727286	2.753944
C	-1.457981	2.729644	3.754895
C	-3.039977	5.014330	3.937326
C	-2.137999	3.011907	4.935353
C	-2.928931	4.155343	5.027480

H	-2.450561	5.393877	1.901783
H	-0.830205	1.845900	3.688447
H	-3.650733	5.906880	4.005894
H	-2.044118	2.345107	5.784434
H	-3.454190	4.378192	5.948890
H	0.174301	2.938962	1.570297
H	-1.509617	1.324000	1.200852

Phosphite anion

Final Gibbs free energy -568.44206718 Eh

Lowest frequency = 279.17 cm^{-1}

O	-0.249133	-0.747643	0.109152
P	0.136643	0.634752	0.558644
O	1.734626	0.794519	0.200130
H	-0.440894	1.583610	-0.306443
O	-0.090453	1.070497	1.982859
H	2.135669	1.553240	0.640286

Characterization of products

General procedure

A 12 mL glass tube with a screw-cap wrapped by sealing tape was charged with stirring bar, dry potassium carbonate (0.125 equiv, 0.181 mmol, 25 mg) and 50% aqueous solution of hypophosphorous acid H_3PO_2 (0.5 equiv, 0.725 mmol, 78.5 μL). After release of CO_2 has ended the obtained homogeneous solution was stirred under reduced pressure (0.2 mmHg) at 80 °C for 25 min to remove about 40 mg of H_2O to yield the mixture of $KH_2PO_2/H_3PO_2/H_2O$ = 1:1:2. Then amine (1.25 equiv, 1.81 mmol) and carbonyl compound (1 equiv, 1.45 mmol) were added to the solution, the tube was sealed and placed into a preheated oil bath where stirring was continued at an indicated temperature. After the indicated time, the reaction mixture was cooled down to the room temperature, washed with 5 mL of DCM and 1 mL of saturated potassium carbonate solution (ca. 50 wt % solution in water), placed into ultrasonic bath for 10 minutes until all solid chunks destroyed and then centrifugated (10 minutes, 8000 rpm). A sample of the resulting solution was analyzed by GC with an external standard or NMR with an internal standard.

4-Cyclohexylmorpholine (1)

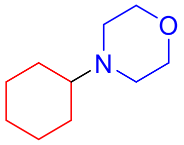

Prepared according to the general procedure. 78% yield by NMR with nitromethane as an internal standard. The residue was purified by column chromatography (eluent: hexane:ethyl acetate 3:1 Rf = 0.3) to afford 172 mg (70%) of the product **1** as a colorless oil.

^{1}H NMR (400 MHz, $CDCl_3$) δ 3.71 (m appears as t, 4H, J = 4.5 Hz), 2.55 (t, 4H, J = 4.5 Hz), 2.20 – 2.07 (m, 1H), 1.89 – 1.82 (m, 2H), 1.80 – 1.72 (m, 2H), 1.64 – 1.59 (m, 1H), 1.26 – 1.04 (m, 5H).

^{13}C NMR (101 MHz, $CDCl_3$) δ 67.5, 63.8, 49.7, 28.9, 26.3, 25.8.

NMR spectra are in agreement with the literature data.[6]

4-Benzyl-1-(4-methoxybenzyl)piperidine (2)

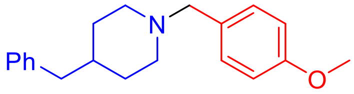

A 12 mL glass tube with a screw-cap wrapped by sealing tape was charged with stirring bar, dry potassium carbonate (0.125 equiv, 0.181 mmol, 25 mg) and 50% aqueous solution of hypophosphorous acid (0.5 equiv, 0.725 mmol, 78.5 μL). After release of CO_2 has ended the obtained homogeneous solution was stirred under reduced pressure (0.2 mmHg) at 80 °C for 25 min to remove about 40 mg of H_2O to yield the mixture of $KH_2PO_2/H_3PO_2/H_2O$ = 1:1:2. Then 4-benzylpiperidine (1.25 equiv, 1.81 mmol, 318 μL) and 4-methoxybenzaldehyde (1 equiv, 1.45 mmol, 197 mg) were added to the solution, the tube was sealed and

placed into a preheated oil bath where stirring was continued at 110 °C for 48 h. After the indicated time, the reaction mixture was cooled down to the room temperature, washed with 5 mL of DCM and 1 mL of saturated potassium carbonate solution (ca. 50 wt % solution in water), placed into ultrasonic bath for 10 minutes until all solid chunks destroyed and then centrifugated (10 minutes, 8000 rpm). 67% yield by NMR with nitromethane as an internal standard. The residue was purified by column chromatography (eluent: hexane:ethyl acetate:triethylamine 1:1:0.05 Rf = 0.24) to afford 218 mg (60%) of the product **2** as an off-white solid.

^{1}H NMR (400 MHz, $CDCl_3$) δ 7.32 – 7.08 (m, 7H), 6.85 (d, J = 8.5 Hz, 2H), 3.80 (s, 3H), 3.42 (s, 2H), 2.86 (dt, J = 11.8, 3.3 Hz, 2H), 2.53 (d, J = 7.0 Hz, 2H), 1.93 – 1.82 (m, 2H), 1.61 (d, J = 12.6 Hz, 2H), 1.58 – 1.44 (m, 1H), 1.31 (qd, J = 11.8, 4.0 Hz, 2H).

^{13}C NMR (101 MHz, $CDCl_3$) δ 158.7, 140.9, 130.7, 130.5, 129.2, 128.2, 125.8, 113.6, 62.9, 55.4, 53.8, 43.4, 38.1, 32.3.

HRMS (ESI+) of $C_{20}H_{25}NO$, *m*/*z*: calcd for $[M+H]^+$ 296.2009, found: 296.2009

Ethyl 1-(4-methoxybenzyl)piperidine-4-carboxylate (3)

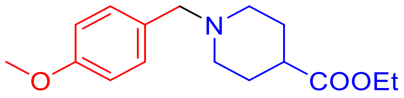

Prepared according to the general procedure. 80% yield by NMR with nitromethane as an internal standard. The residue was purified by flash chromatograph InterChim PuriFlash (eluent: hexane:ethyl acetate 2:1 Rf = 0.3) to afford 261 mg (65% yield) of the product **3** as a yellowish oil.

^{1}H NMR (400 MHz, $CDCl_3$) δ 7.21 (d, J = 8.6 Hz, 2H), 6.84 (d, J = 8.6 Hz, 2H), 4.12 (q, J = 7.1 Hz, 2H), 3.79 (s, 3H), 3.42 (s, 2H), 2.83 (d, J = 11.2 Hz, 2H), 2.26 (tt, J = 11.2, 4.1 Hz, 1H), 2.05 – 1.93 (m, 2H), 1.90 – 1.67 (m, 4H), 1.23 (t, J = 7.1 Hz, 3H).

^{13}C NMR (101 MHz, $CDCl_3$) δ 175.4, 158.7, 130.5, 130.3, 113.6, 62.7, 60.3, 55.3, 52.9, 41.4, 28.4, 14.3.

HRMS (ESI+) of $C_{16}H_{23}NO_3$, *m*/*z*: calcd for $[M+H]^+$ 278.1751, found: 278.1750

***N*-Butyl-*N*-(4-methoxybenzyl)butan-1-amine (4)**

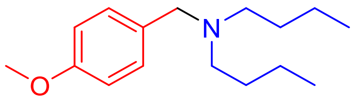

A 12 mL glass tube with a screw-cap wrapped by sealing tape was charged with stirring bar, dry potassium carbonate (0.125 equiv, 0.181 mmol, 25 mg) and 50% aqueous solution of hypophosphorous acid (0.5 equiv, 0.725 mmol, 78.5 μL). After release of CO_2 has ended the obtained homogeneous solution was stirred under reduced pressure (0.2 mmHg) at 80 °C for 25 min to remove about 40 mg of H_2O to yield

the mixture of $KH_2PO_2/H_3PO_2/H_2O$ = 1:1:2. Then dibutylamine (2.5 equiv, 3.62 mmol, 611 μL) and 4-methoxybenzaldehyde (1 equiv, 1.45 mmol, 197 mg) were added to the solution, the tube was sealed and placed into a preheated oil bath where stirring was continued at 110 °C for 19 h. After the indicated time, the reaction mixture was cooled down to the room temperature, washed with 5 mL of DCM and 1 ml of saturated potassium carbonate solution (ca. 50 wt % solution in water), placed into ultrasonic bath for 10 minutes until all solid chunks destroyed and then centrifugated (10 minutes, 8000 rpm). 89% yield by NMR with nitromethane as an internal standard. The residue was purified by column chromatography (eluent: hexane:ethyl acetate 4:1 Rf = 0.45) to afford 167 mg (68%) of the product **4** as a colorless oil.

^{1}H NMR (400 MHz, $CDCl_3$) δ 7.23 (d, J = 8.4 Hz, 2H), δ 6.85 (d, J = 8.4 Hz, 2H), δ 3.80 (s, 3H), 3.48(s, 2H), 2.38 (t, J = 7.5 Hz, 4H), 1.44 (p, J = 7.3 Hz, 4H), 1.29 (h, J = 7.3 Hz, 4H), 0.88 (t, J = 7.3 Hz, 6H).

^{13}C NMR (101 MHz, $CDCl_3$) δ 158.5, 132.4, 130.0, 113.5, 58.0, 55.4, 53.5, 29.4, 20.8, 14.2.

NMR spectra are in agreement with the literature data.[7]

N-Allyl-*N*-(4-methoxybenzyl)prop-2-en-1-amine (5)

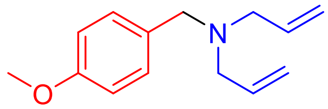

Prepared according to the general procedure. 73% yield by NMR with nitromethane as an internal standard. The residue was purified by column chromatography (eluent: hexane:ethyl acetate 6:1 Rf = 0.45) to afford 173 mg (57% yield) of the product **5** as a yellow oil.

^{1}H NMR (400 MHz, $CDCl_3$) δ 7.22 (d, J = 8.4 Hz, 2H), 6.83 (d, J = 8.4 Hz, 2H), 5.95 – 5.71 (m, 2H), 5.25 – 5.06 (m, 4H), 3.78 (s, 3H), 3.50 (s, 2H), 3.05 (d, J = 6.3 Hz, 4H).

^{13}C NMR (101 MHz, $CDCl_3$) δ 158.6, 136.1, 131.4, 130.2, 117.5, 113.6, 56.9, 56.4, 55.3.

HRMS (ESI+) of $C_{16}H_{23}NO_3$, *m/z*: calcd for $[M+H]^+$ 218.1540, found: 218.1542

*N*1-Ethyl-*N*1-(4-methoxybenzyl)-*N*2,*N*2-dimethylethane-1,2-diamine (6)

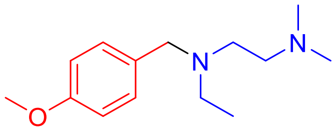

Prepared according to the general procedure. 80% yield by NMR with nitromethane as an internal standard. The residue was purified by column chromatography (eluent: ethyl acetate:methanol:triethylamine 3:1:0.05 Rf = 0.3) to afford 226 mg (66% yield) of the product **6** as a brown oil.

^{1}H NMR (400 MHz, $CDCl_3$) δ 7.22 (d, J = 8.5 Hz, 2H), 6.83 (d, J = 8.5 Hz, 2H), 3.78 (s, 3H), 3.52 (s, 2H), 2.63 – 2.44 (m, 4H), 2.39 (dd, J = 8.8, 5.8 Hz, 2H), 2.19 (s, 6H), 1.03 (t, J = 7.1 Hz, 3H).

^{13}C NMR (101 MHz, $CDCl_3$) δ 158.6, 131.6, 130.1, 113.6, 57.9, 57.6, 55.3, 51.1, 47.6, 46.0, 11.6.

HRMS (ESI+) of $C_{14}H_{24}N_2O$, *m*/*z*: calcd for $[M+H]^+$ 237.1962, found: 237.1966.

4-(4-Methoxybenzyl)morpholine (7)

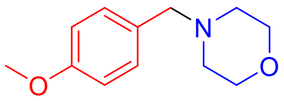

Prepared according to the general procedure. 85% yield by NMR with nitromethane as an internal standard. The residue was purified by column chromatography (eluent: hexane:ethyl acetate:triethylamine 2:1:0.05 Rf = 0.3) to afford 231 mg (77% yield) of the product **7** as a yellow oil.

^{1}H NMR (400 MHz, $CDCl_3$) δ 7.23 (d, J = 8.6 Hz, 2H), 6.85 (d, J = 8.6 Hz, 2H), 3.80 (s, 3H), 3.69 (t, J = 4.7 Hz, 4H), 3.44 (s, 2H), 2.42 (t, J = 4.7 Hz, 4H).

^{13}C NMR (101 MHz, $CDCl_3$) δ 158.9, 130.5, 129.8, 113.7, 67.1, 62.9, 55.3, 53.6.

NMR spectra are in agreement with the literature data.[8]

2-(4-(4-Methoxybenzyl)piperazin-1-yl)pyrimidine (8)

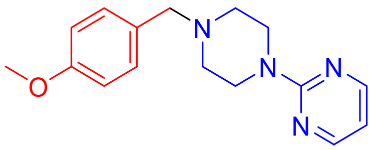

Prepared according to the general procedure. 61% yield by NMR with nitromethane as an internal standard. The residue was purified by column chromatography (eluent: hexane/ethyl acetate 1:4 Rf = 0.35) to afford 215 mg (50%) of the product **8** as an off-white solid.

^{1}H NMR (400 MHz, $CDCl_3$) δ 8.29 (d, J = 4.7 Hz, 2H), 7.25 (d, J = 8.5 Hz, 2H), 6.87 (d, J = 8.5 Hz, 2H), 6.46 (t, J = 4.7 Hz, 1H), 3.86 – 3.75 (m, 7H), 3.48 (s, 2H), 2.48 (t, J = 4.9 Hz, 4H).

^{13}C NMR (101 MHz, $CDCl_3$) δ 161.79, 158.89, 157.78, 130.46, 130.06, 113.75, 109.79, 62.64, 55.34, 52.97, 43.80.

HRMS (ESI+) of $C_{16}H_{20}N_4O$, *m*/*z*: calcd for $[M+H]^+$ 285.1710, found: 285.1708.

2-(4-(Benzo[*d*][1,3]dioxol-5-ylmethyl)piperazin-1-yl)pyrimidine (9)

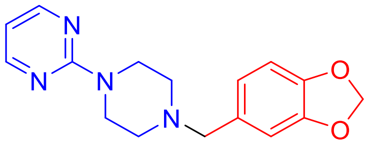

A 12 mL glass tube with a screw-cap wrapped by sealing tape was charged with stirring bar, dry potassium carbonate (0.125 equiv, 0.062 mmol, 9 mg) and 50% aqueous solution of hypophosphoric acid (0.5 equiv, 0.362 mmol, 39 µL). After release of CO_2 has ended the obtained homogeneous solution was stirred under reduced pressure (0.2 mmHg) at 80 °C for 25 min to remove about 13 mg of H_2O to yield the mixture of $KH_2PO_2/H_3PO_2/H_2O$ = 1:1:2 Then 1-(2-pyrimidyl)piperazine (1.25 equiv, 0.625 mmol, 89 µL) and piperonal (1 equiv, 0.5 mmol, 75 mg) were added to the solution, the tube was sealed and placed into a preheated oil bath where stirring was continued at 110 °C for 19 h. After the indicated time, the reaction mixture was cooled down to the room temperature, washed with 5 mL of DCM and 1 mL of saturated potassium carbonate solution (ca. 50 wt % solution in water), placed into ultrasonic bath for 10 minutes until all solid chunks destroyed and then centrifugated (10 minutes, 8000 rpm). 80% yield by NMR. The residue was purified by column chromatography (eluent: ethyl acetate:hexane 7:2 Rf = 0.3) to afford 122 mg (80%) of the product **9** as a yellow solid.

^{1}H NMR (400 MHz, $CDCl_3$) δ 8.29 (d, J = 4.8 Hz, 2H), 6.88 (s, 1H), 6.75 (s, 2H), 6.46 (t, J = 4.8 Hz, 1H), 5.94 (s, 2H), 3.81 (t, J = 5.1 Hz, 4H), 3.44 (s, 2H), 2.47 (t, J = 5.1 Hz, 4H).

^{13}C NMR (101 MHz, $CDCl_3$) δ 161.8, 157.8, 147.8, 146.8, 132.0, 122.3, 109.8, 109.6, 108.0, 101.0, 63.0, 53.0, 43.8.

NMR spectra are in agreement with the literature data.[9]

4-(3-Methoxybenzyl)morpholine (10)

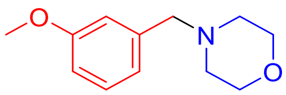

Prepared according to the general procedure. 89% yield by NMR with nitromethane as an internal standard. The residue was purified by column chromatography (eluent: hexane/ethyl acetate/triethylamine 2:1:0.05 Rf = 0.3) to afford 215 mg (71%) of the product **10** as a yellow oil.

^{1}H NMR (400 MHz, $CDCl_3$) δ 7.23 (t, J = 8.0 Hz, 1H, 6.91 (d, J = 4.9 Hz, 2H), 6.80 (d, J = 8.0 Hz, 1H), 3.81 (s, 3H), 3.69 (t, J = 4.5 Hz, 4H), 3.47 (s, 2H), 2.44 (t, J = 4.5 Hz, 4H).

^{13}C NMR (101 MHz, $CDCl_3$) δ 159.6, 139.5, 129.2, 121.4, 114.6, 112.5, 67.0, 63.4, 55.2, 53.6.

NMR spectra are in agreement with the literature data.[6]

***N*-Butyl-*N*-(3-(4-isopropylphenyl)-2-methylpropyl)butan-1-amine (11)**

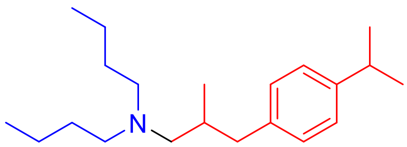

Prepared according to the general procedure. 53% yield by NMR with nitromethane as an internal standard. The residue was purified by column chromatography (eluent: hexane/ethyl acetate/triethylamine 19:1:0.2 Rf = 0.3) to afford 209 mg (50% yield) of the product **11** as a yellow oil.

^{1}H NMR (400 MHz, $CDCl_3$) δ 7.15 (d, J = 8.0 Hz, 2H), 7.09 (d, J = 8.0 Hz, 2H), 2.96 – 2.82 (m, 2H), 2.46 – 2.33 (m, 4H), 2.25 (dd, J = 12.6, 7.3 Hz, 1H), 2.20 – 2.11 (m, 2H), 1.93 – 1.79 (m, 1H), 1.45 – 1.29 (m, 8H), 1.26 (d, J = 7.1 Hz, 6H), 0.92 (t, J = 7.2 Hz, 6H), 0.83 (d, J = 6.5 Hz, 3H).

^{13}C NMR (101 MHz, $CDCl_3$) δ 146.1, 139.3, 129.2, 126.2, 61.8, 54.7, 41.3, 34.3, 33.8, 29.6, 24.2, 20.9, 18.3, 14.3.

HRMS (ESI+) of $C_{19}H_{37}N$, *m*/*z*: calcd for $[M+H]^+$ 304.2999, found: 304.2998

N-Allyl-*N*-(3-(4-isopropylphenyl)-2-methylpropyl)prop-2-en-1-amine (12)

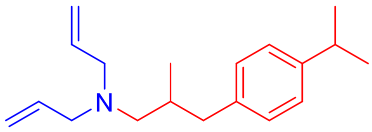

Prepared according to the general procedure. 71% yield by NMR with nitromethane as an internal standard. The residue was purified by flash chromatograph InterChim PuriFlash (eluent: hexane/ethyl acetate/triethylamine 19:1:0.2 Rf = 0.3) to afford 160 mg (43% yield) of the product **12** as a yellow oil.

^{1}H NMR (300 MHz, $CDCl_3$) δ 7.14 (d, J = 8.0 Hz, 2H), 7.07 (d, J = 8.0 Hz, 2H), 5.94 – 5.76 (m, 2H), 5.27 – 5.06 (m, 4H), 3.08 (d, J = 6.4 Hz, 4H), 2.95 – 2.77 (m, 2H), 2.35 – 2.13 (m, 3H), 1.99 – 1.79 (m, 1H), 1.25 (d, J = 6.9 Hz, 6H), 0.84 (d, J = 6.5 Hz, 3H).

^{13}C NMR (101 MHz, $CDCl_3$) δ 146.2, 139.0, 136.4, 129.2, 126.2, 117.1, 60.3, 57.5, 41.2, 33.8 (2C), 24.2, 18.2.

HRMS (ESI+) of $C_{19}H_{29}N$, *m*/*z*: calcd for $[M+H]^+$ 272.2373, found: 272.2373

4-(2-Methoxybenzyl)morpholine (13)

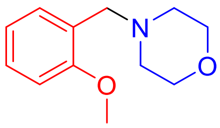

Prepared according to the general procedure. 87 % yield by NMR with nitromethane as an internal standard. The residue was purified by column chromatography (eluent: hexane/ethyl acetate/triethylamine 2:1:0.05 Rf = 0.3) to afford 200 mg (66%) of the product **13** as a yellow oil.

^{1}H NMR (400 MHz, $CDCl_3$) δ 7.36 (d, J = 7.4 Hz, 1H), 7.24 (t, J = 8.2 Hz, 1H), 6.94 (t, J = 7.4 Hz, 1H), 6.87 (d, J = 8.2 Hz, 1H), 3.82 (s, 3H), 3.76 – 3.69 (m, 4H), 3.56 (s, 2H), 2.55 – 2.44 (m, 4H).

^{13}C NMR (101 MHz, $CDCl_3$) δ 157.9, 130.6, 128.2, 125.9, 120.4, 110.6, 67.2, 56.5, 55.5, 53.7.

NMR spectra are in agreement with the literature data.[6]

N,N-dibenzyl-1-cyclohexylmethanamine (14)

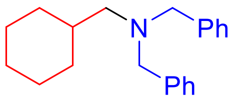

Prepared according to the general procedure. 67% yield by NMR with nitromethane as an internal standard. The residue was purified by purified by column chromatography (eluent: hexane:ethyl acetate:triethylamine 20:1:0.2 Rf = 0.6) to afford 278 mg (65% yield) of the product **14** as a white solid.

^{1}H NMR (300 MHz, $CDCl_3$) δ 7.48 – 7.21 (m, 10H), 3.55 (s, 4H), 2.22 (d, J = 7.2 Hz, 2H), 1.88 (dd, J = 12.2, 3.7 Hz, 2H), 1.76 – 1.53 (m, 4H), 1.32 – 1.05 (m, 3H), 0.87 – 0.69 (m, 2H).

^{13}C NMR (101 MHz, $CDCl_3$) δ 140.3, 128.9, 128.2, 126.8, 60.9, 58.9, 35.8, 31.8, 27.0, 26.3.

NMR spectra are in agreement with the literature data.[10]

N-(1-Phenylethyl)cyclohexanamine (15)

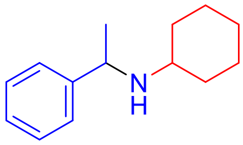

Prepared according to the general procedure. 60% yield by NMR with dimethylformamide as an internal standard (78% in 48 hours). The residue was purified by column chromatography (eluent: hexane/ethyl acetate/triethylamine 2:1:0.03 Rf = 0.3) to afford 124 mg (42% yield) of the product **15** as a yellow oil.

^{1}H NMR (400 MHz, $CDCl_3$) δ 7.35 – 7.27 (m, 4H), 7.26 – 7.20 (m, 1H), 3.96 (q, J = 6.6 Hz, 1H), 2.26 (tt, J = 10.1, 3.7 Hz, 1H), 2.04 – 1.93 (m, 1H), 1.75 – 1.60 (m, 3H), 1.60 – 1.50 (m, 1H), 1.33 (d, J = 6.6 Hz, 3H), 1.17 – 0.96 (m, 5H).

^{13}C NMR (101 MHz, $CDCl_3$) δ 146.5, 128.5, 126.8, 126.6, 54.5, 53.7, 34.7, 33.3, 26.3, 25.4, 25.2, 25.1.

NMR spectra are in agreement with the literature data.[11]

4-(3-Nitrobenzyl)morpholine (16)

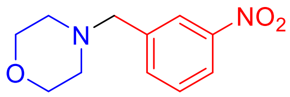

Prepared according to the general procedure, but with prolongated reaction time – 48 hours. 78% yield by NMR with nitromethane as an internal standard.

Prepared according to the general procedure. 59% yield by NMR with nitromethane as an internal standard. The residue was purified by column chromatography (eluent: hexane/ethyl acetate 1:1 Rf = 0.25) to afford 150 mg (47% yield) of the product **16** as a red crystals.

^{1}H NMR (400 MHz, $CDCl_3$) δ 8.21 (t, J = 2,2 Hz, 1H), 8.11 (dd, J = 7.9, 2.2 Hz, 1H), 7.67 (d, J = 7.9 Hz, 1H), 7.48 (t, J = 7.9 Hz, 1H), 3.71 (m appears as t, J = 4.6 Hz, 4H), 3.58 (s, 2H), 2.45 (m appears as t, J = 4.6 Hz, 4H).

^{13}C NMR (101 MHz, $CDCl_3$) δ 148.5, 140.5, 135.1, 129.3, 123.9, 122.4, 67.0, 62.5, 53.7.

NMR spectra are in agreement with the literature data.[6]

4-(4-Chlorobenzyl)morpholine (17)

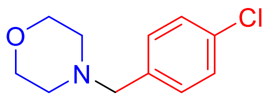

Prepared according to the general procedure, but with prolongated reaction time – 48 hours. 78% yield by NMR with nitromethane as an internal standard.

Prepared according to the general procedure. 59% yield by NMR with nitromethane as an internal standard. The residue was purified by column chromatography (eluent: hexane/ethyl acetate 1:1 Rf = 0.3) to afford 132 mg (43% yield) of the product **17** as a yellow oil.

^{1}H NMR (400 MHz, $CDCl_3$) δ 7.33 – 7.16 (m, 4H), 3.68 (m appears as t, J = 4.1 Hz, 4H), 3.44 (s, 2H), 2.41 (m appears as t, J = 4.1 Hz, 4H).

^{13}C NMR (101 MHz, $CDCl_3$) δ 136.5, 132.9, 130.5, 128.5, 67.1, 62.7, 53.7.

NMR spectra are in agreement with the literature data.[12]

4-(3-(Trifluoromethyl)benzyl)morpholine (18)

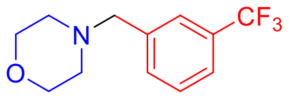

Prepared according to the general procedure, but with prolongated reaction time – 48 hours. 80% yield by NMR with nitromethane as an internal standard.

Prepared according to the general procedure. 63% yield by NMR with nitromethane as an internal standard. The residue was purified by column chromatography (eluent: hexane/ethyl acetate 3:2 Rf = 0.3) to afford 162 mg (46% yield) of the product **18** as an orange oil.

^{1}**H NMR** (400 MHz, $CDCl_3$) δ 7.60 (s, 1H), 7.56 – 7.47 (m, 2H), 7.48 – 7.39 (m, 1H), 3.72 (m appears as t, J = 4.6 Hz, 4H), 3.54 (s, 2H), 2.44 (m appears as t, J = 4.6 Hz, 4H).

^{13}**C NMR** (101 MHz, $CDCl_3$) δ 139.2, 132.5 (d, J = 1.5 Hz), 130.8 (q appears as d, J = 32.2 Hz), 128.8, 125.8 (q, J = 3.8 Hz), 124.4 (q, J = 272.7 Hz), 124.2 (q, J = 3.8 Hz), 67.1, 63.0, 53.7.

^{19}**F NMR** (376 MHz, $CDCl_3$) δ -62.54.

NMR spectra are in agreement with the literature data. [13]

4-(Morpholinomethyl)benzonitrile (19)

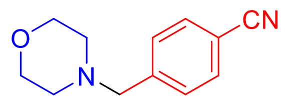

Prepared according to the general procedure, but with prolongated reaction time – 48 hours. 58% yield by NMR with nitromethane as an internal standard.

Prepared according to the general procedure. 54% yield by NMR with nitromethane as an internal standard. The residue was purified by column chromatography (eluent: hexane/ethyl acetate 3:2 Rf = 0.3) to afford 110 mg (38% yield) of the product **19** as a red crystals.

^{1}**H NMR** (400 MHz, $CDCl_3$) δ 7.60 (d, J = 8.0 Hz, 2H), 7.45 (d, J = 7.9 Hz, 2H), 3.70 (m appears as t, J = 4.7 Hz, 4H), 3.53 (s, 2H), 2.43 (m appears as t, J = 4.6 Hz, 4H).

^{13}**C NMR** (101 MHz, $CDCl_3$) δ 143.9, 132.2, 129.6, 119.0, 111.1, 67.0, 62.9, 53.7.

NMR spectra are in agreement with the literature data.[14]

^{1}H and ^{13}C NMR spectra of the products

^{1}H spectrum of 4-cyclohexylmorpholine (1) ($CDCl_3$, 400 MHz)

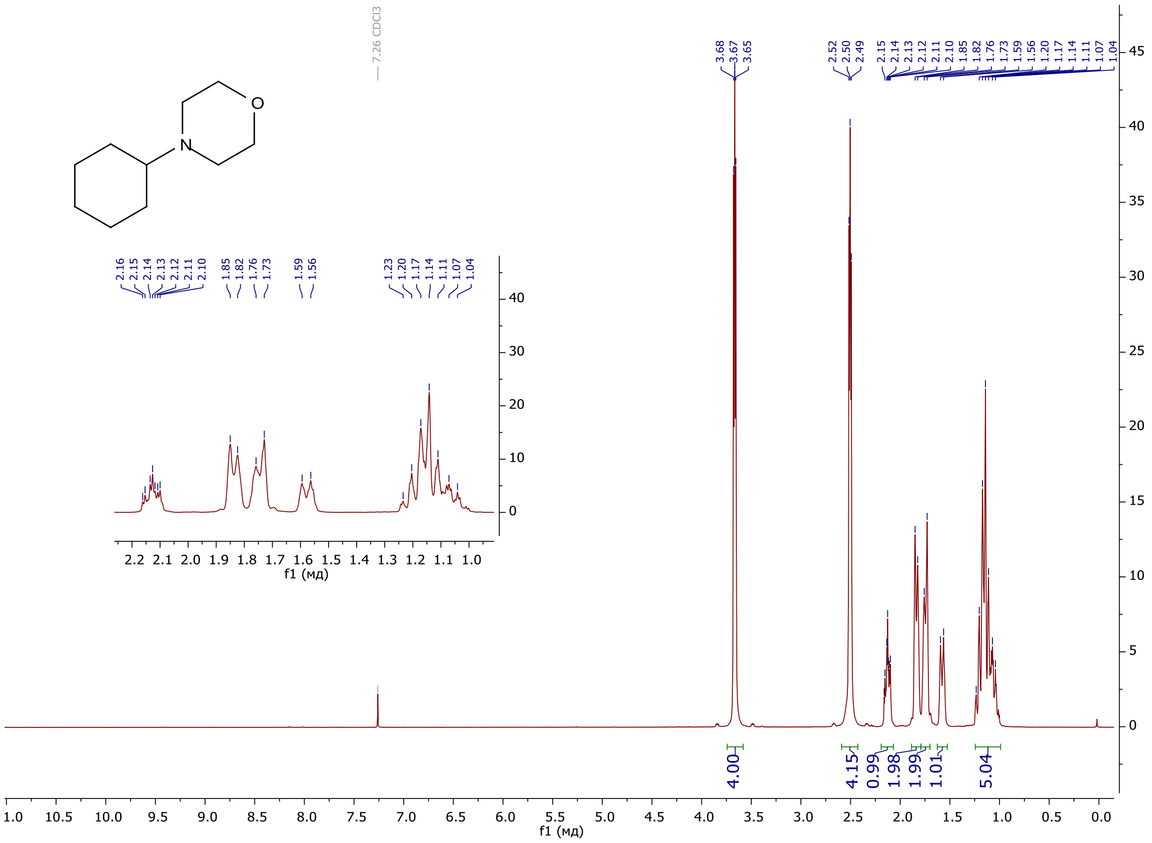

^{13}C spectrum of 4-cyclohexylmorpholine (1) ($CDCl_3$, 101 MHz)

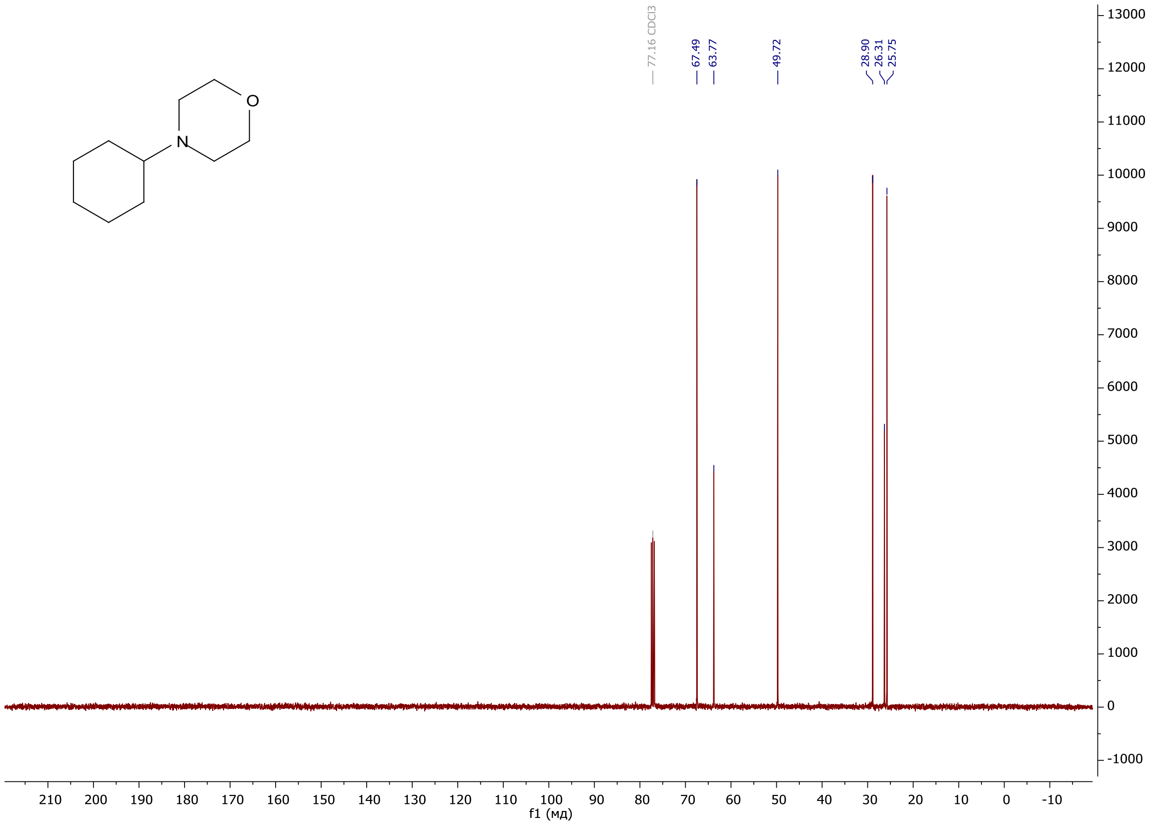

^{1}H spectrum of 4-benzyl-1-(4-methoxybenzyl)piperidine (2) ($CDCl_3$, 400 MHz)

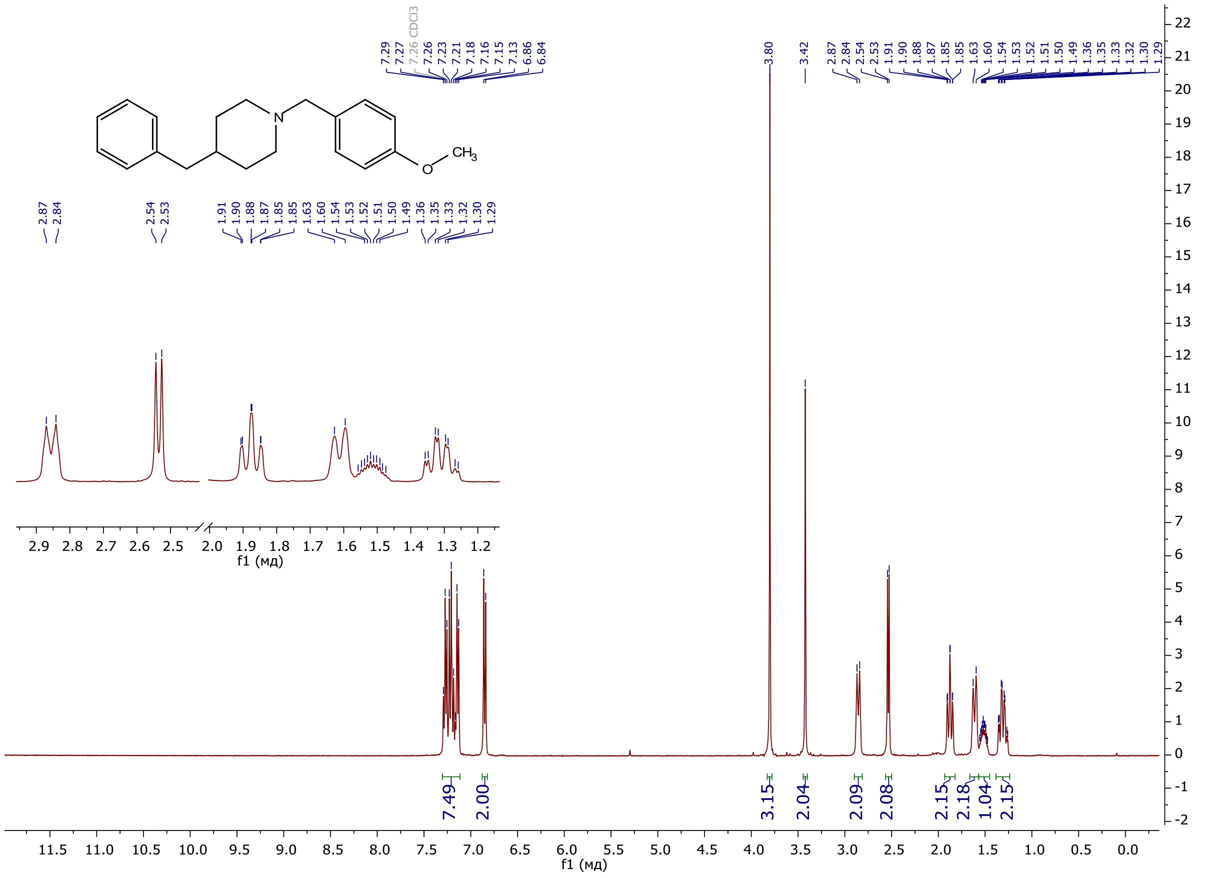

13C spectrum of 4-benzyl-1-(4-methoxybenzyl)piperidine (2) (CDCl3, 101 MHz)

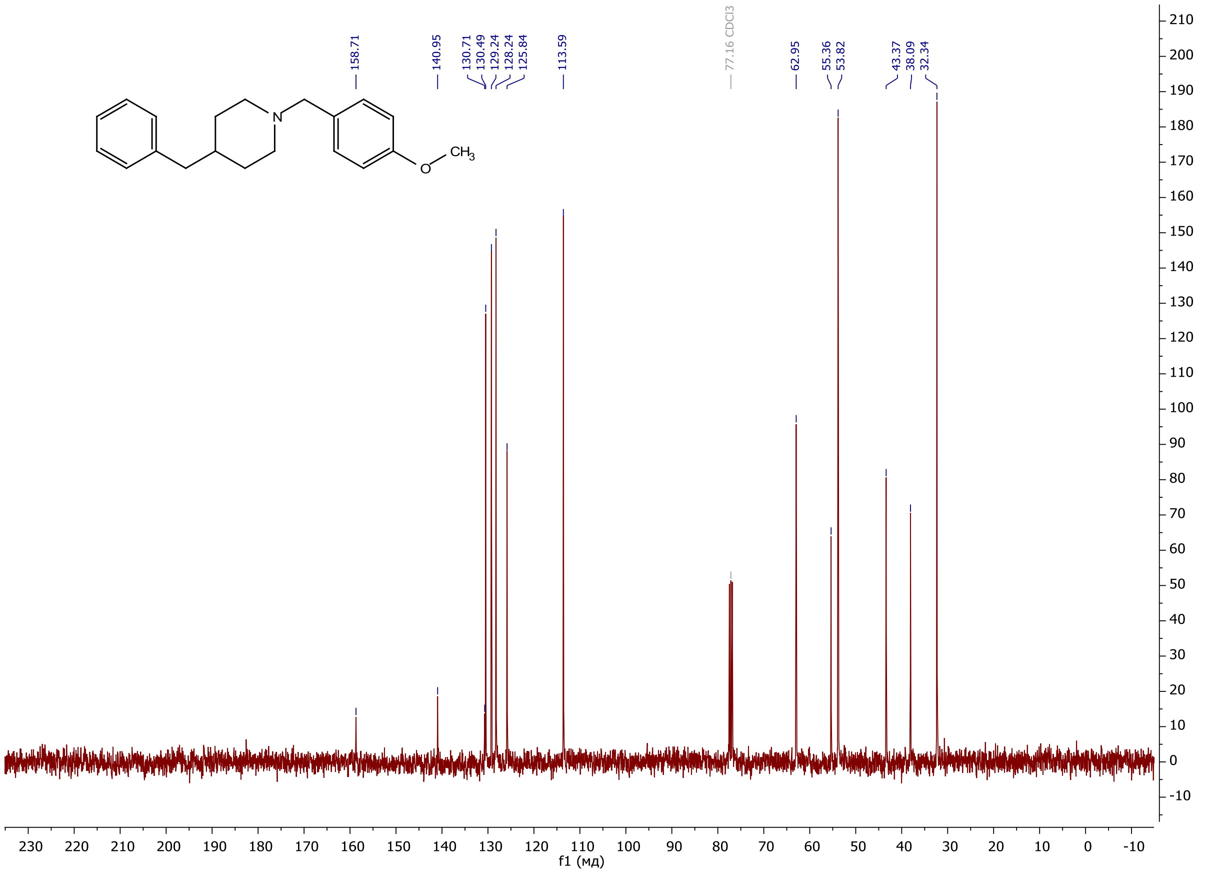

^{1}H spectrum of ethyl 1-(4-methoxybenzyl)piperidine-4-carboxylate (3) ($CDCl_3$, 400 MHz)

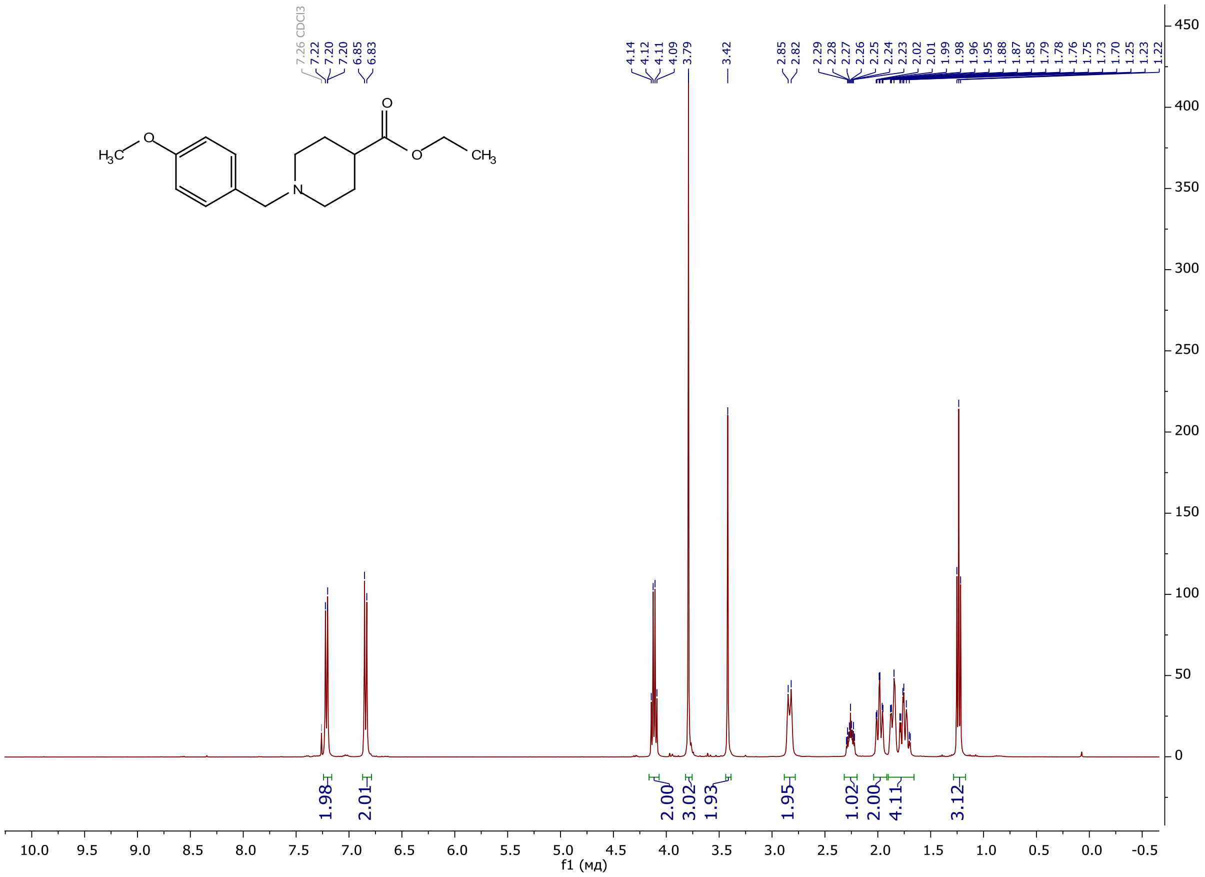

^{13}C spectrum of ethyl 1-(4-methoxybenzyl)piperidine-4-carboxylate (3) ($CDCl_3$, 101 MHz)

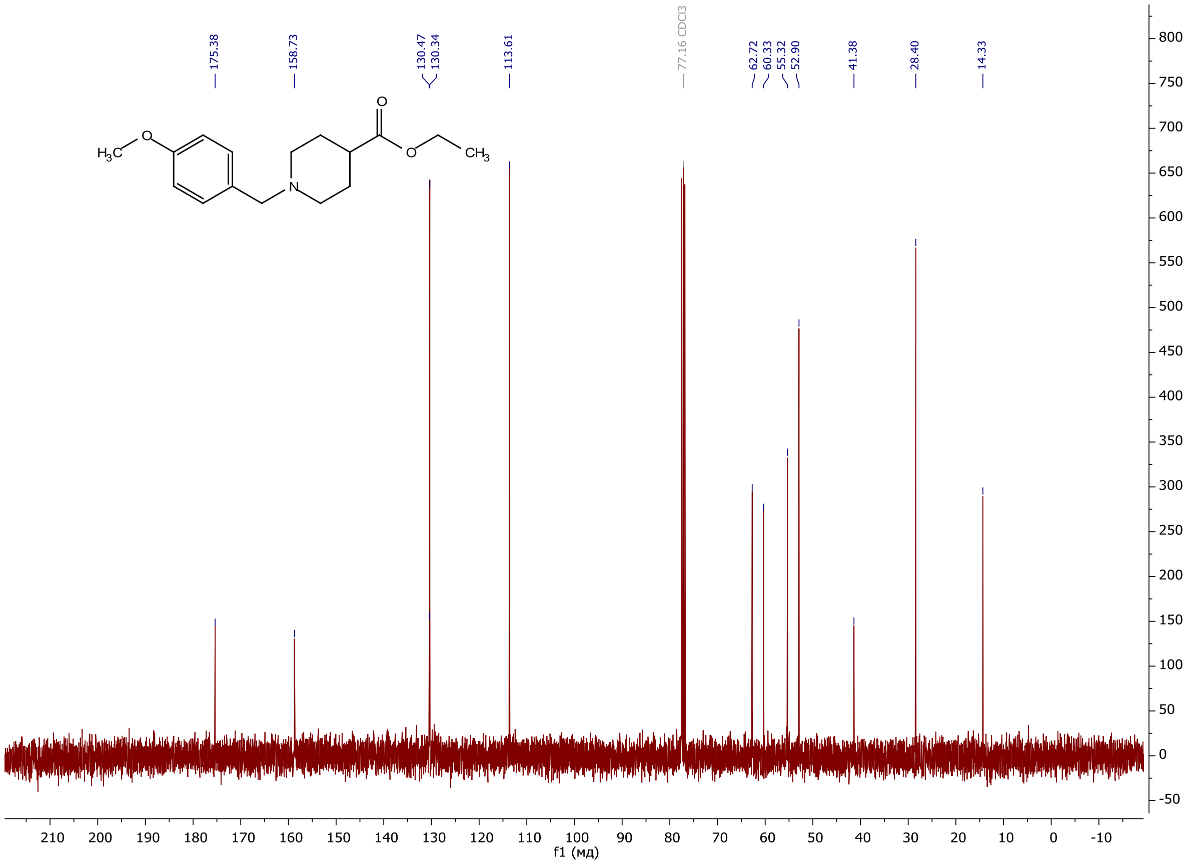

^{1}H spectrum of *N*-butyl-*N*-(4-methoxybenzyl)butan-1-amine (4) ($CDCl_3$, 400 MHz)

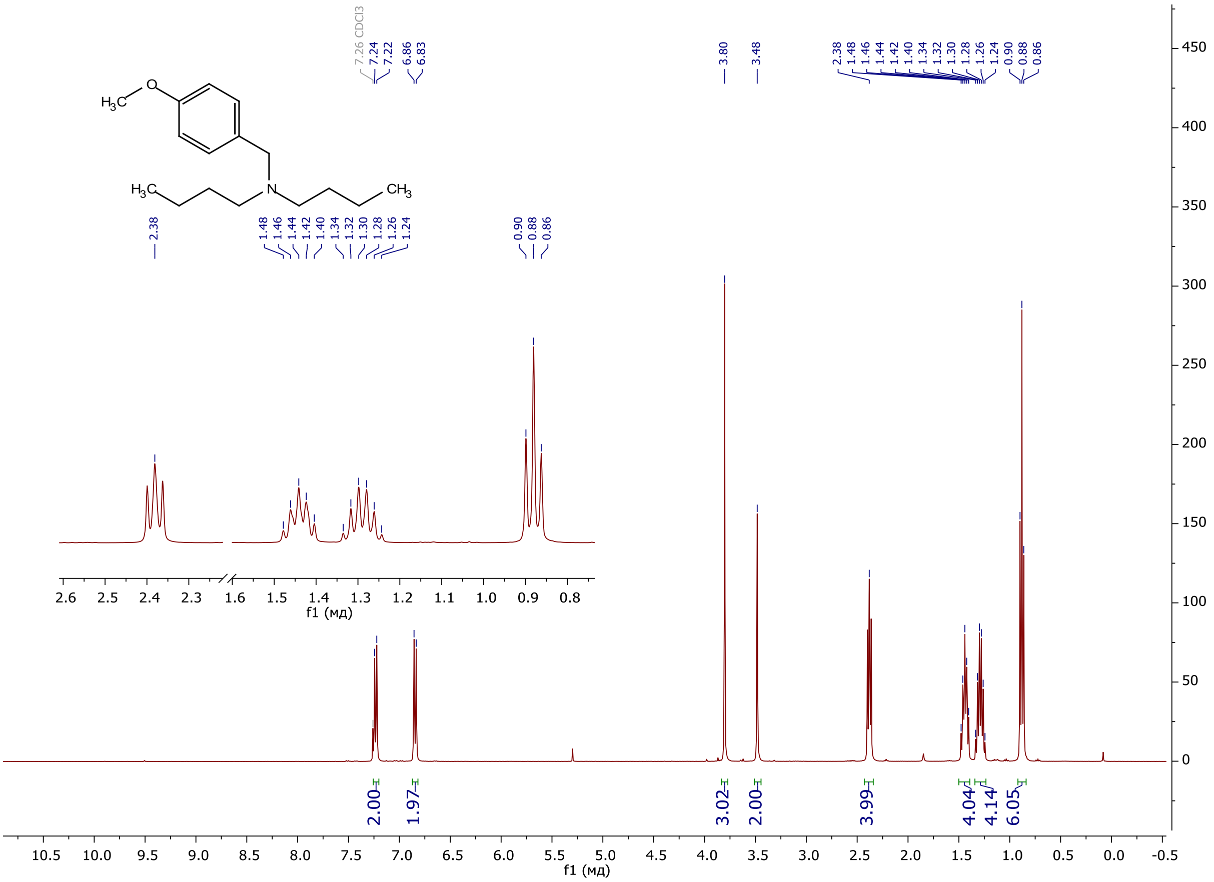

^{13}C spectrum of *N*-butyl-*N*-(4-methoxybenzyl)butan-1-amine (4) ($CDCl_3$, 101 MHz)

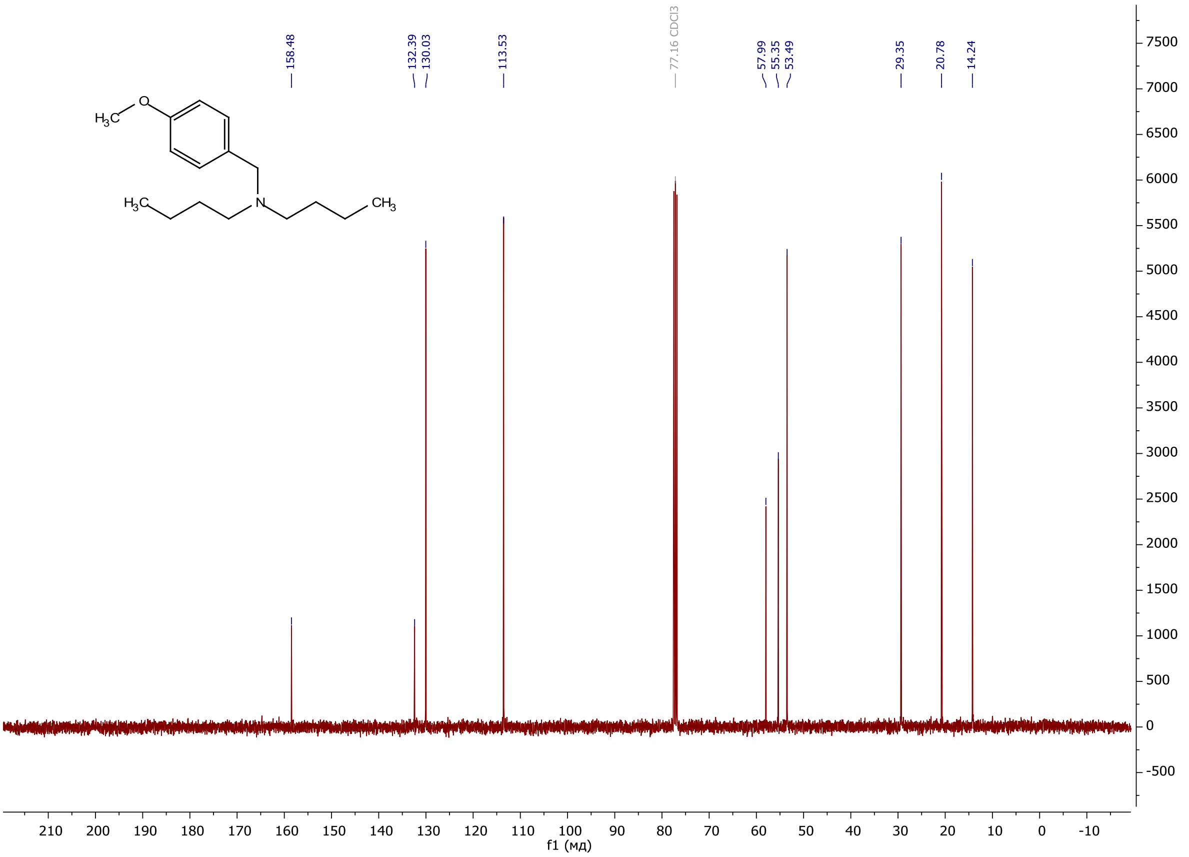

^{1}H spectrum of *N*-allyl-*N*-(4-methoxybenzyl)prop-2-en-1-amine (5) ($CDCl_3$, 400 MHz)

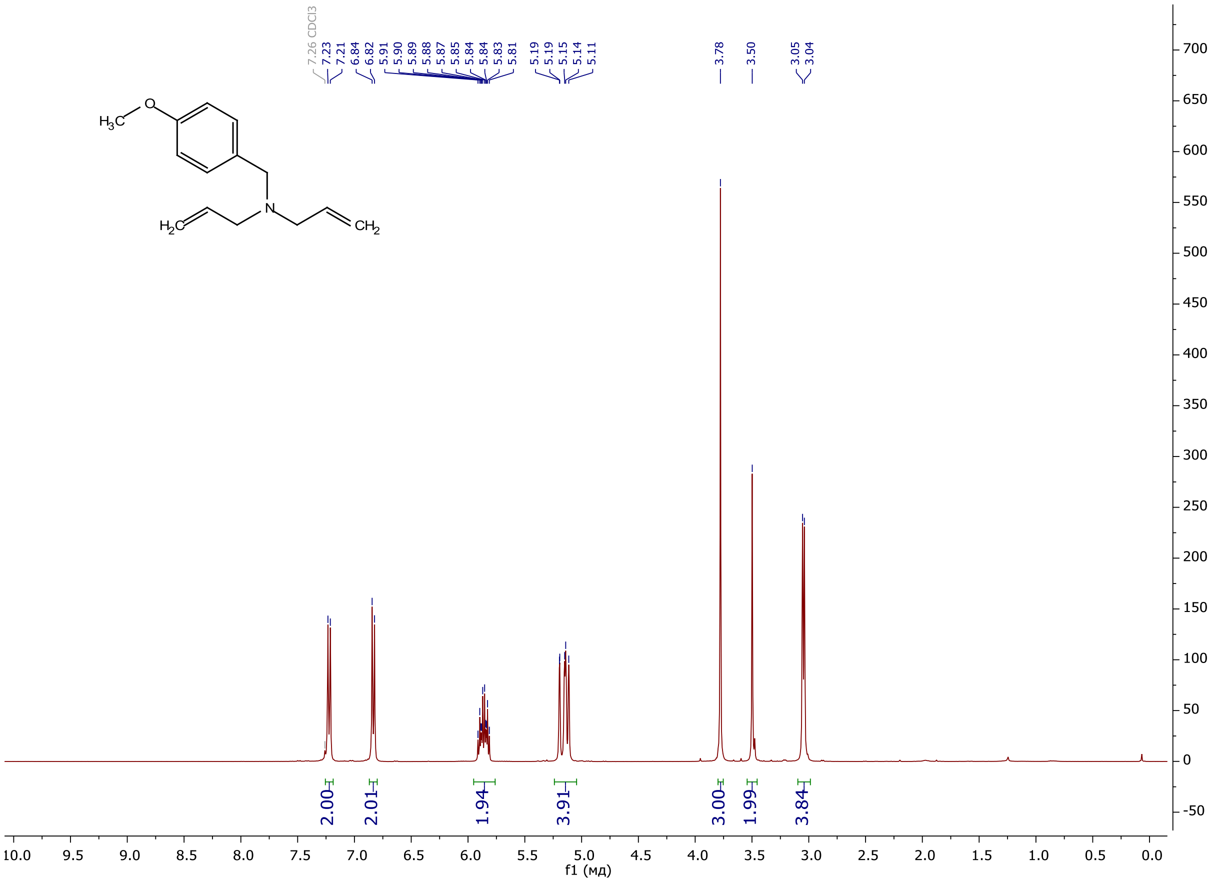

^{13}C spectrum of *N*-allyl-*N*-(4-methoxybenzyl)prop-2-en-1-amine (5) ($CDCl_3$, 101 MHz)

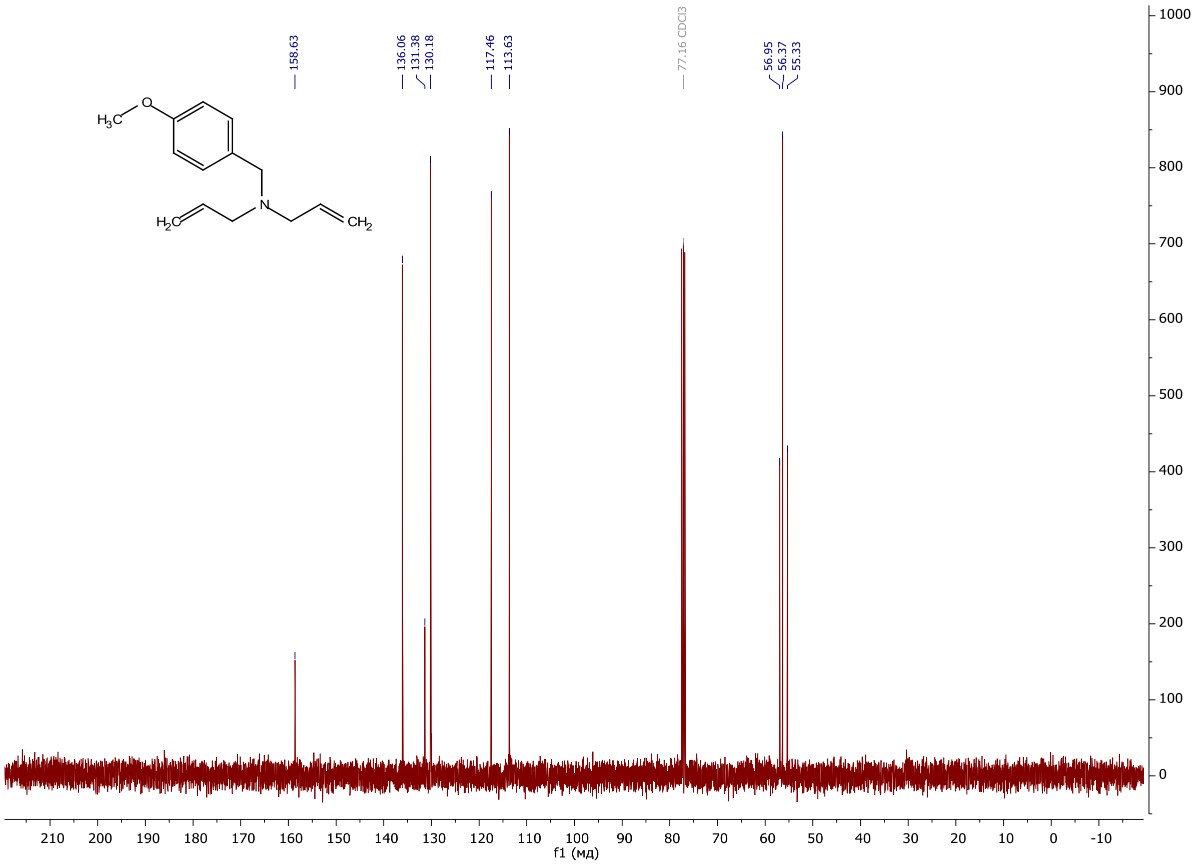

^{1}H spectrum of *N*1-ethyl-*N*1-(4-methoxybenzyl)-*N*2,*N*2-dimethylethane-1,2-diamine (6) ($CDCl_3$, 400 MHz)

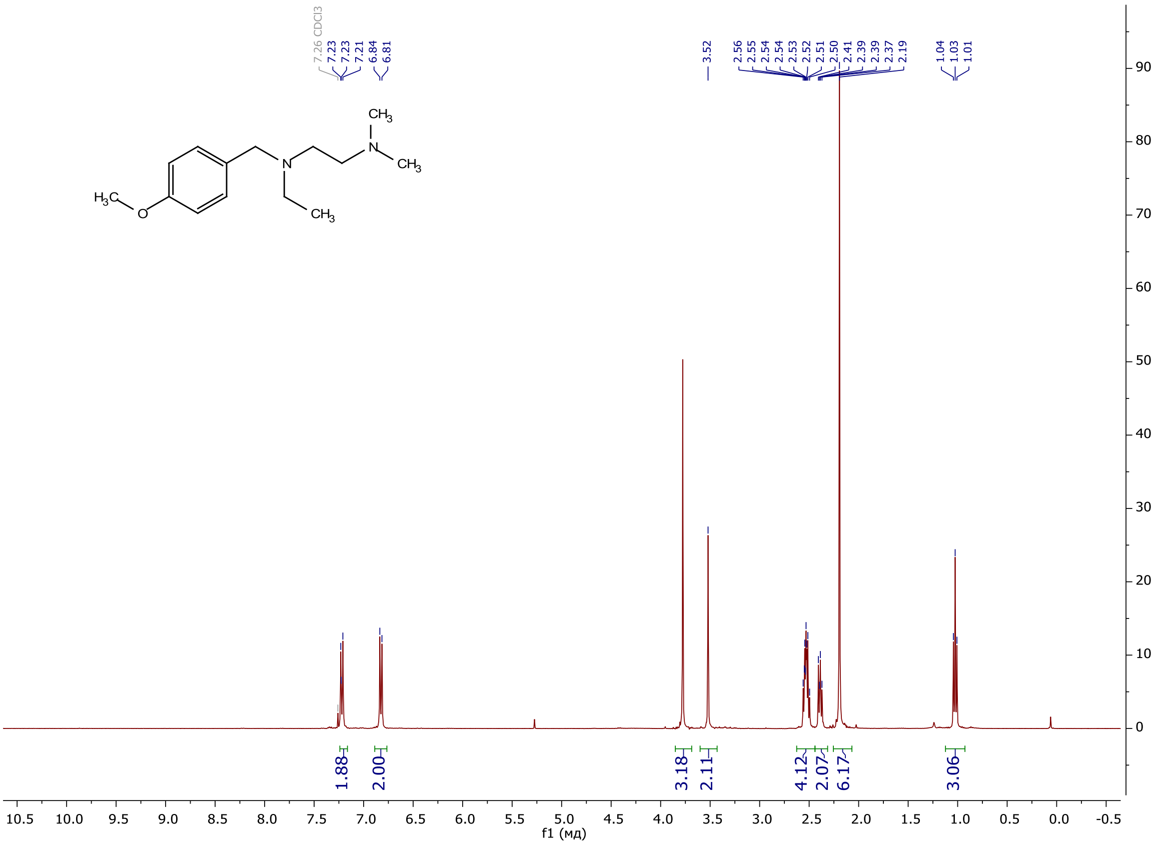

^{13}C spectrum of *N*1-ethyl-*N*1-(4-methoxybenzyl)-*N*2,*N*2-dimethylethane-1,2-diamine (6) ($CDCl_3$, 101 MHz)

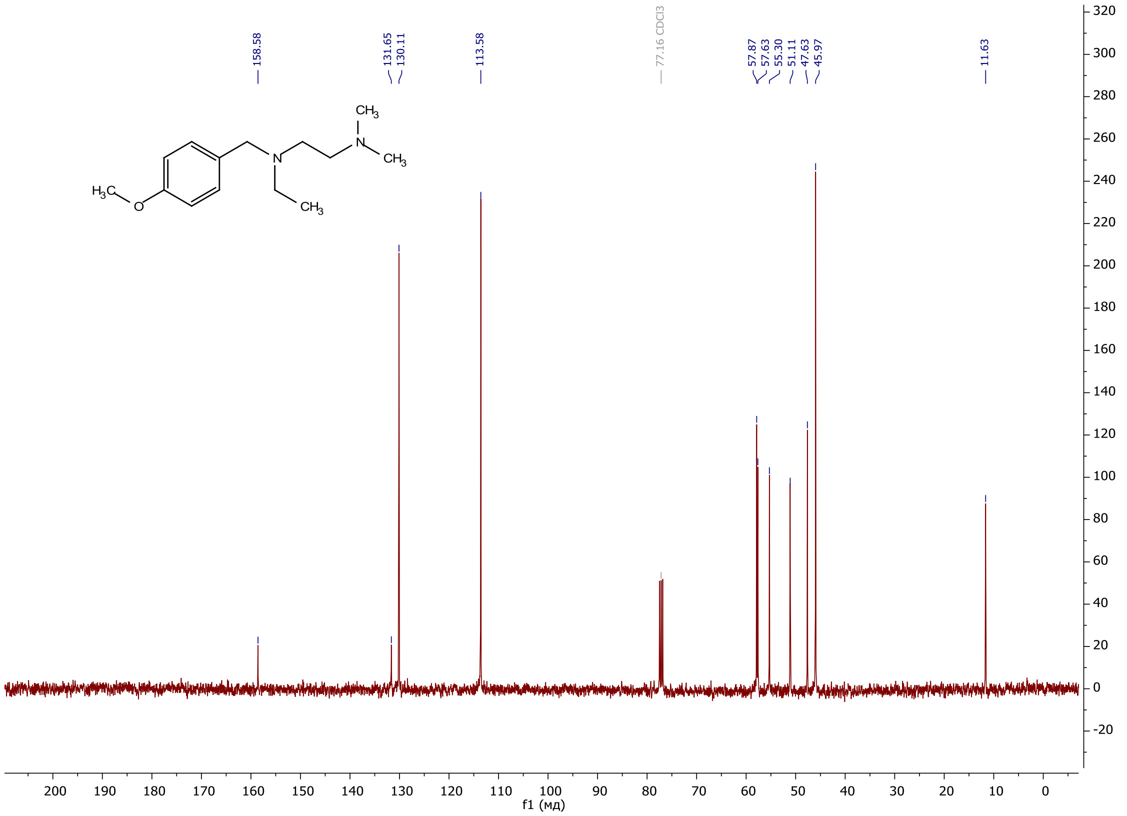

^{1}H spectrum of 4-(4-methoxybenzyl)morpholine (7) ($CDCl_3$, 400 MHz)

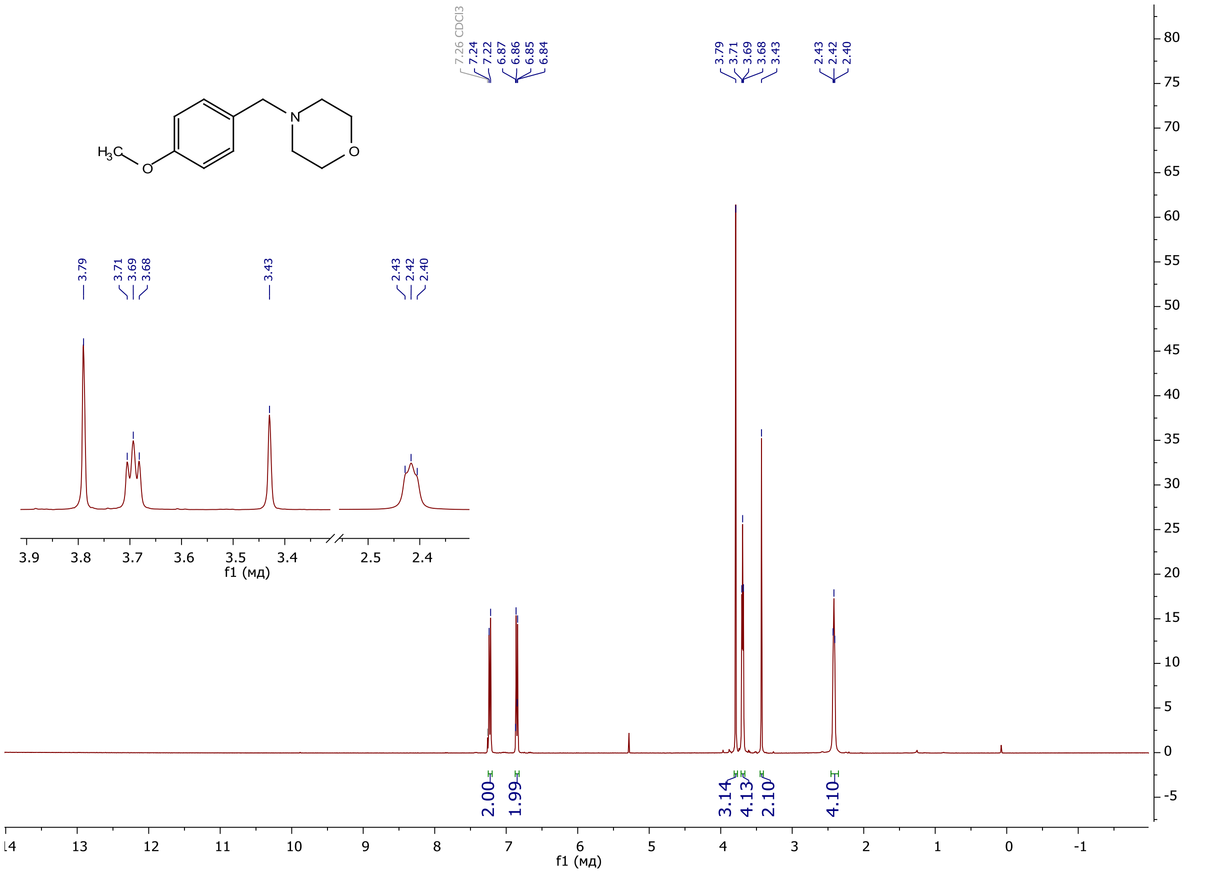

^{13}C spectrum of 4-(4-methoxybenzyl)morpholine (7) ($CDCl_3$, 101 MHz)

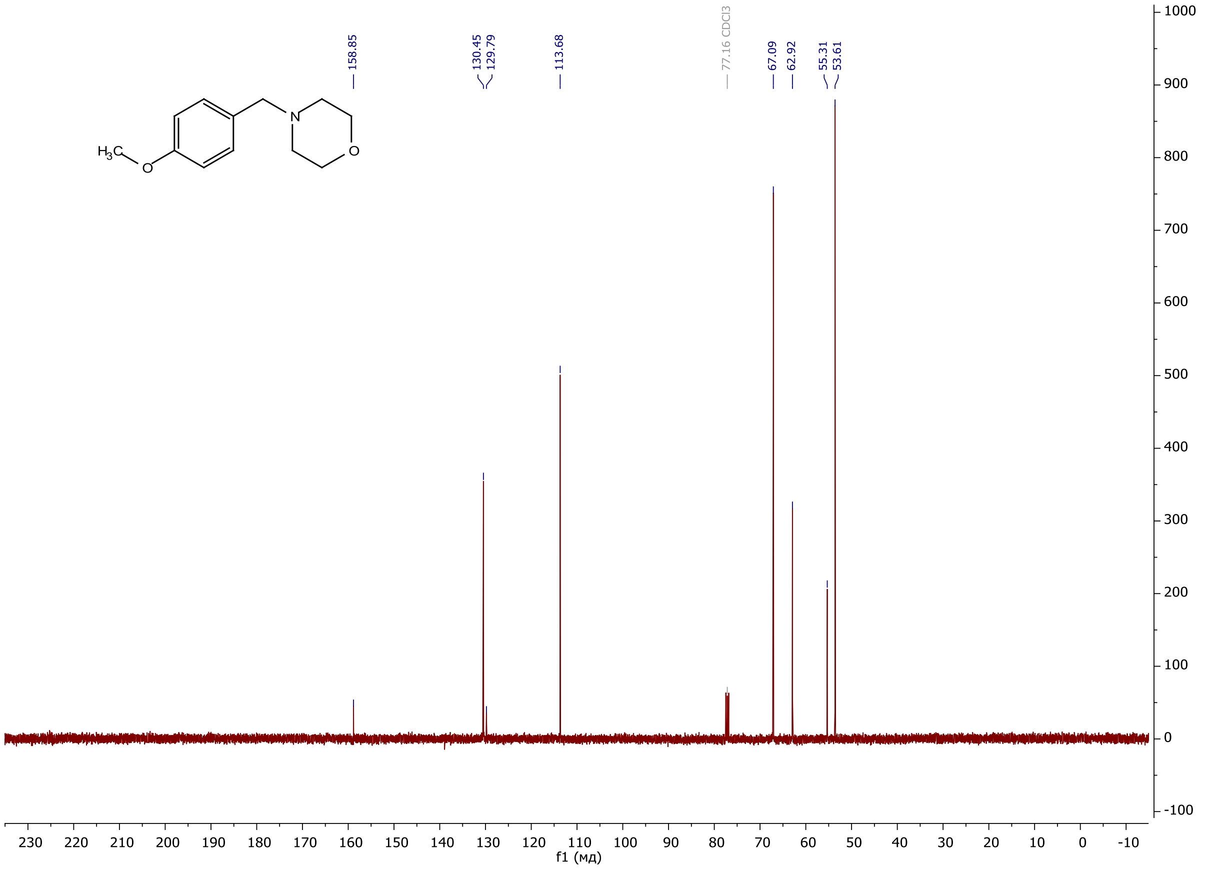

^{1}H spectrum of 2-(4-(4-methoxybenzyl)piperazin-1-yl)pyrimidine (8) ($CDCl_3$, 400 MHz)

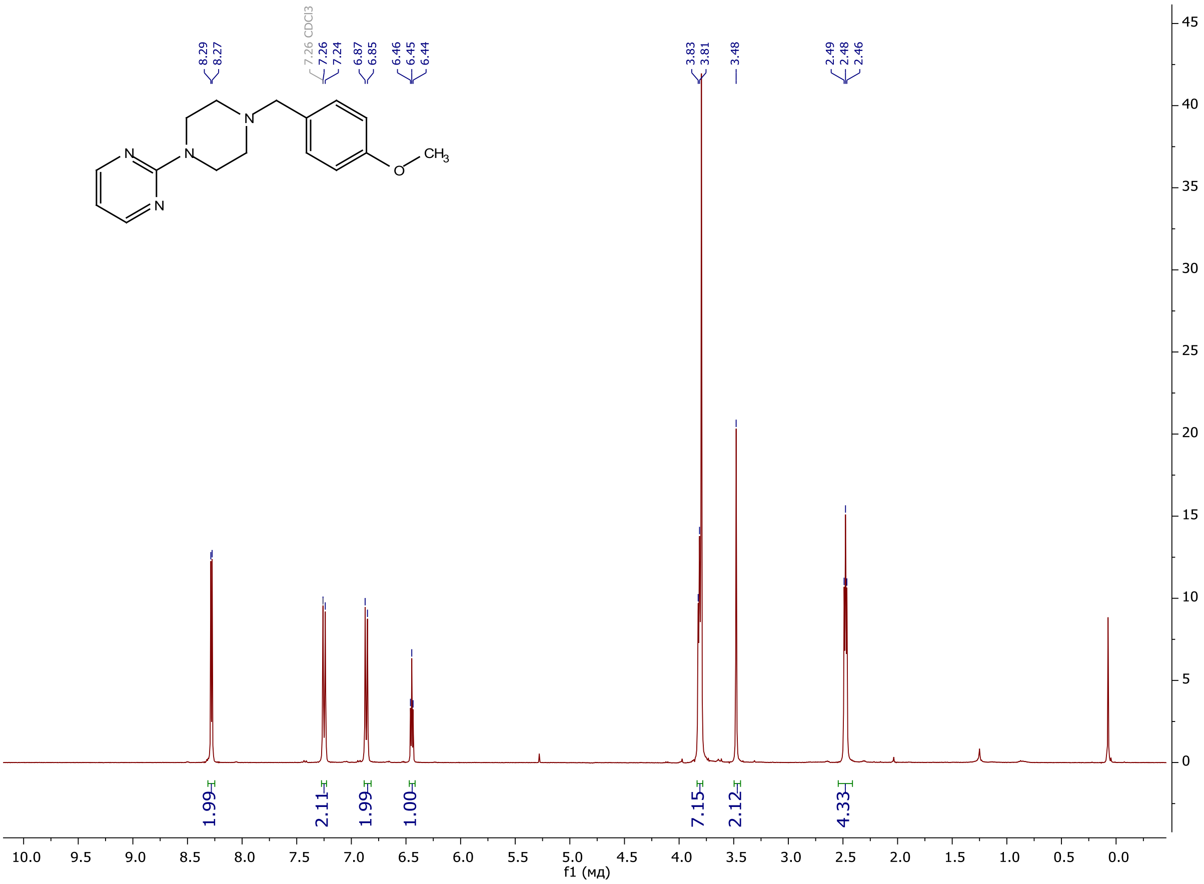

^{13}C spectrum of 2-(4-(4-methoxybenzyl)piperazin-1-yl)pyrimidine (8) ($CDCl_3$, 101 MHz)

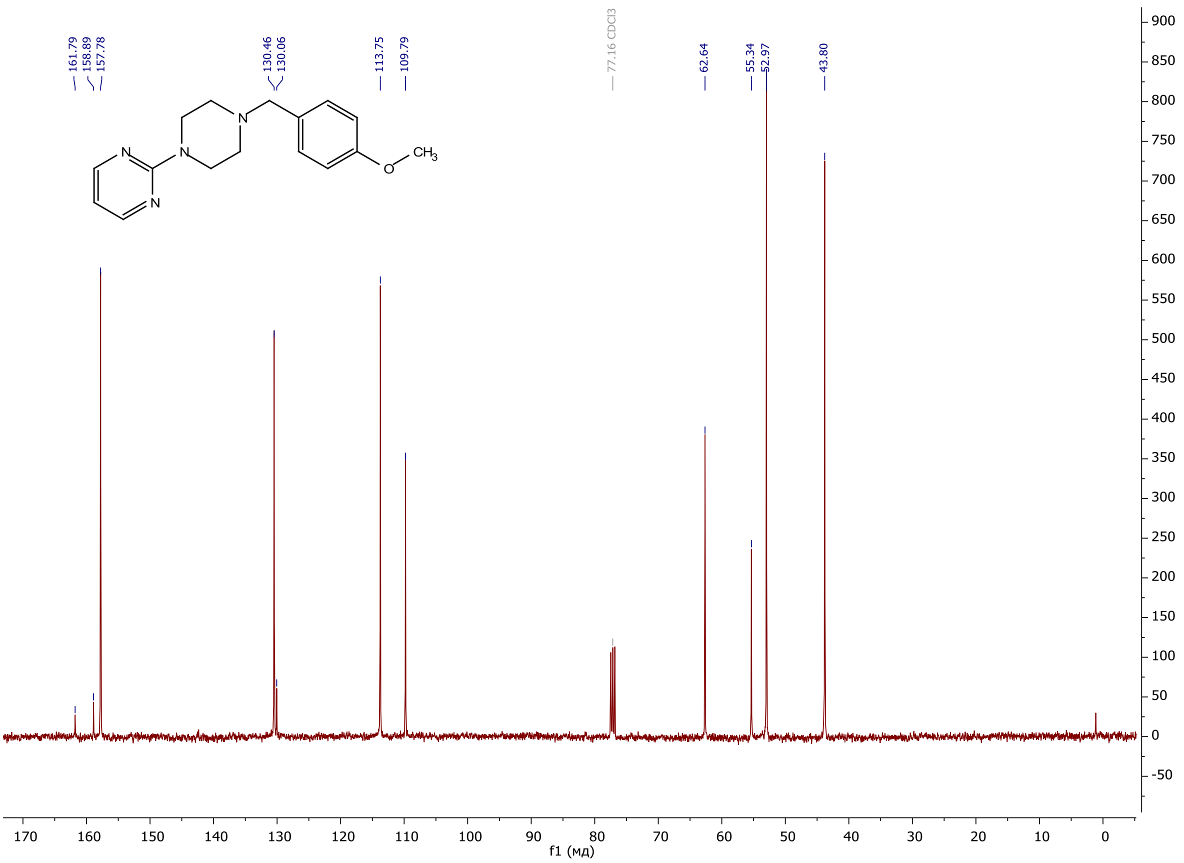

^{1}H spectrum of 2-(4-(benzo[*d*][1,3]dioxol-5-ylmethyl)piperazin-1-yl)pyrimidine (9) ($CDCl_3$, 400 MHz)

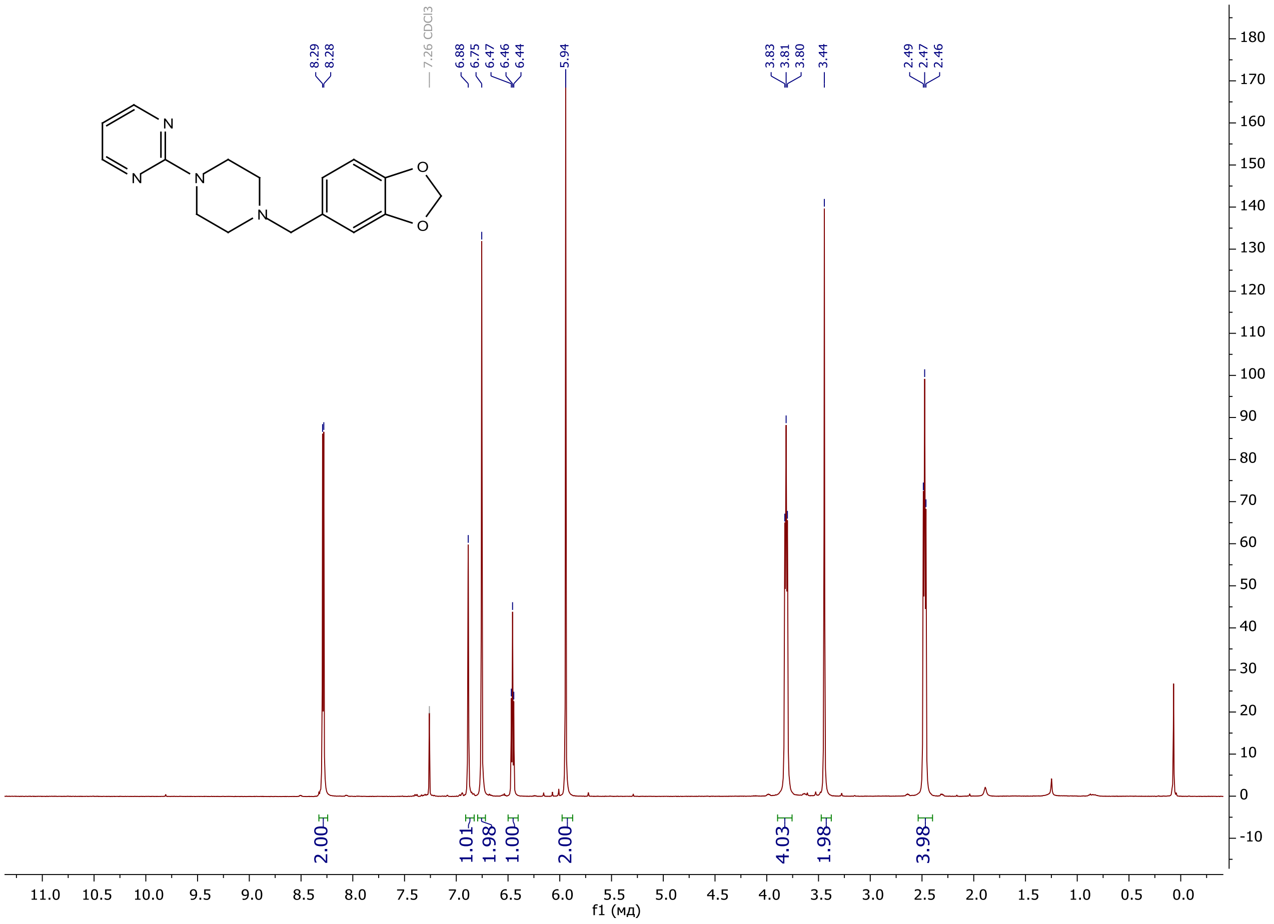

^{13}C spectrum of 2-(4-(benzo[*d*][1,3]dioxol-5-ylmethyl)piperazin-1-yl)pyrimidine (9) ($CDCl_3$, 101 MHz)

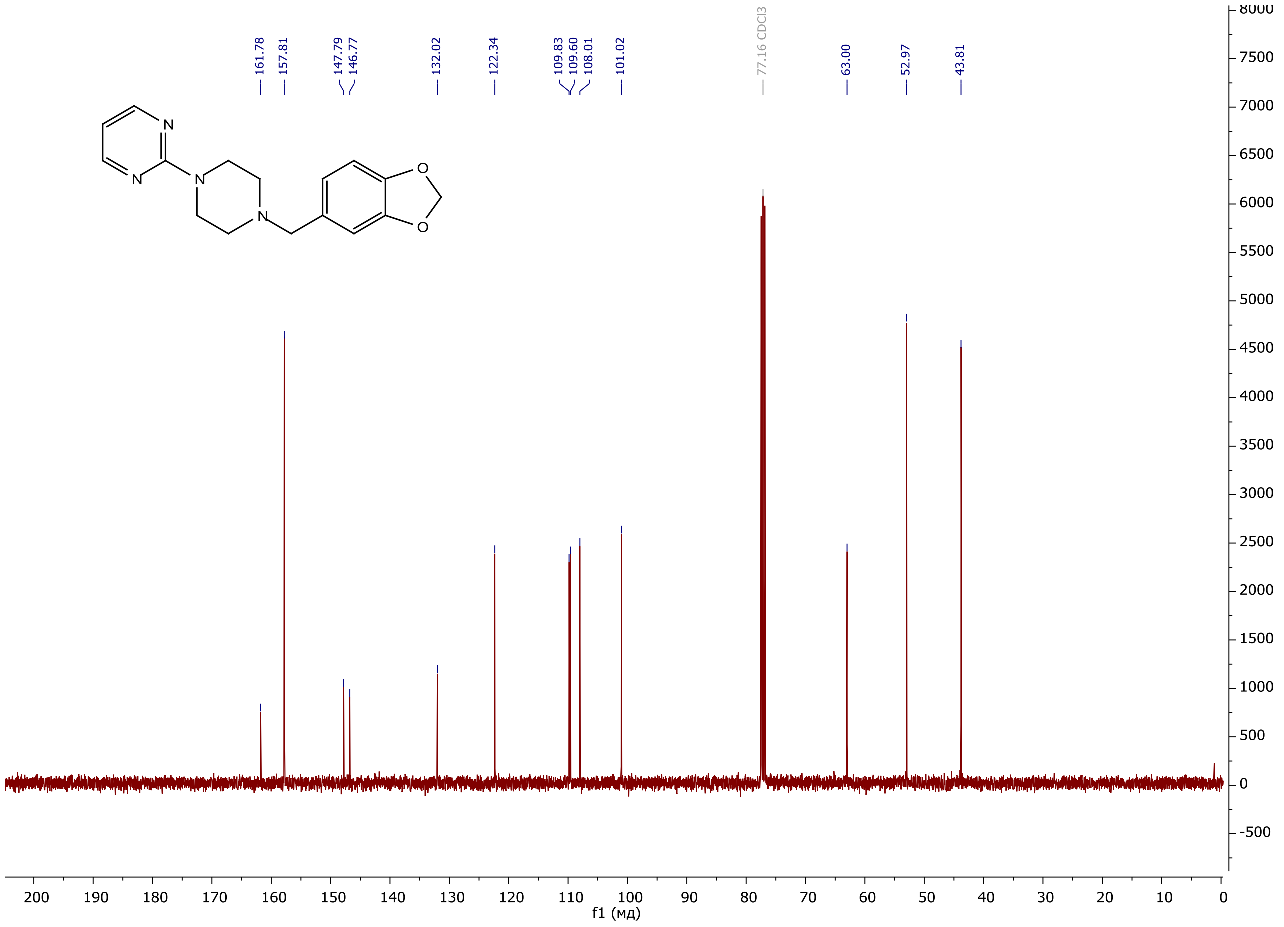

^{1}H spectrum of 4-(3-methoxybenzyl)morpholine (10) ($CDCl_3$, 400 MHz)

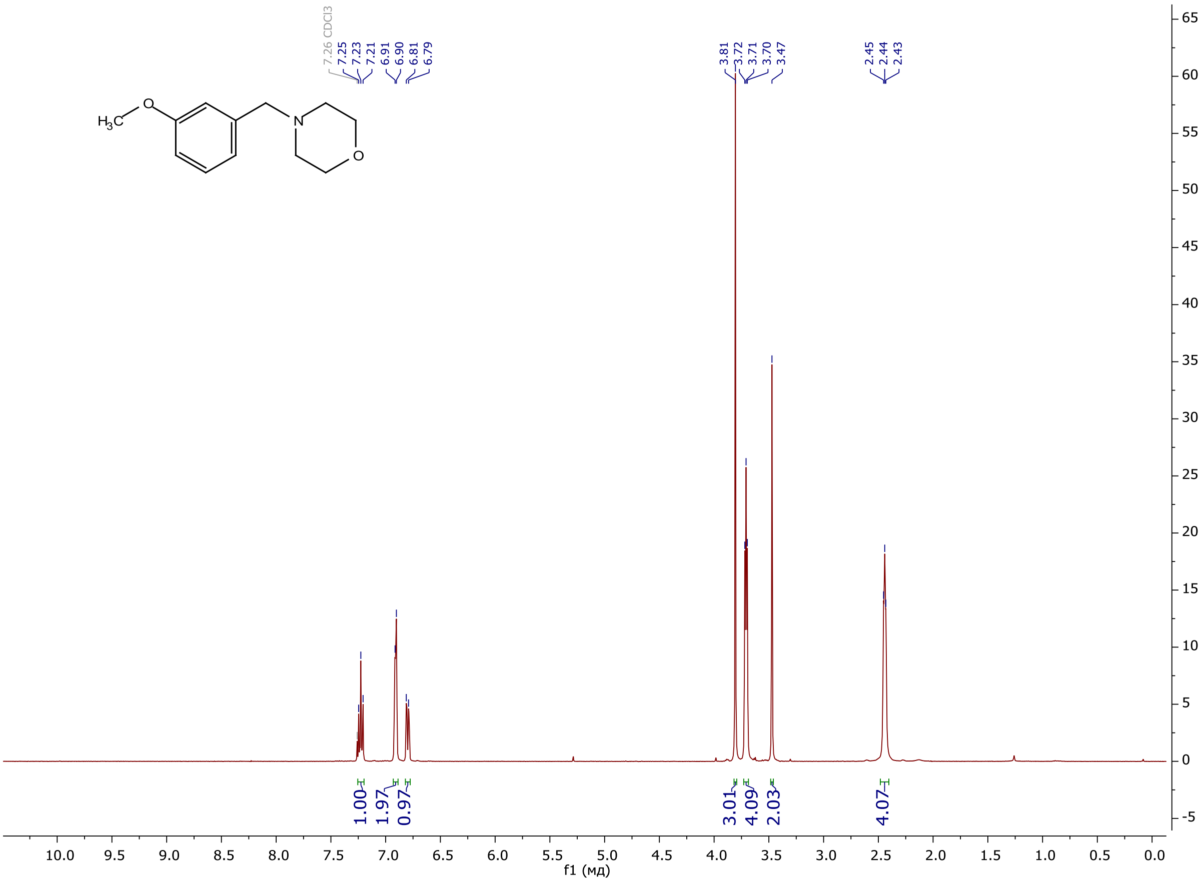

^{13}C spectrum of 4-(3-methoxybenzyl)morpholine (10) ($CDCl_3$, 101 MHz)

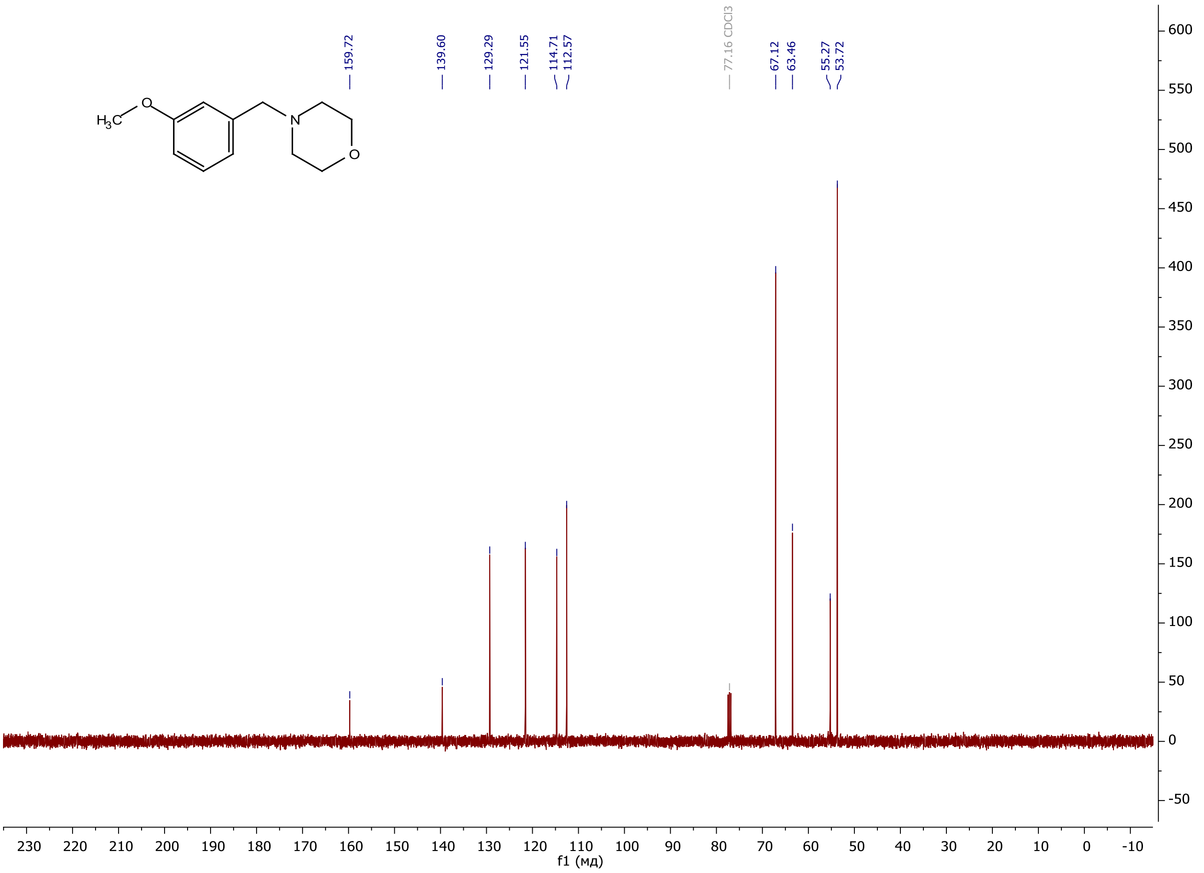

^{1}H spectrum of *N*-butyl-*N*-(3-(4-isopropylphenyl)-2-methylpropyl)butan-1-amine (11) ($CDCl_3$, 400 MHz)

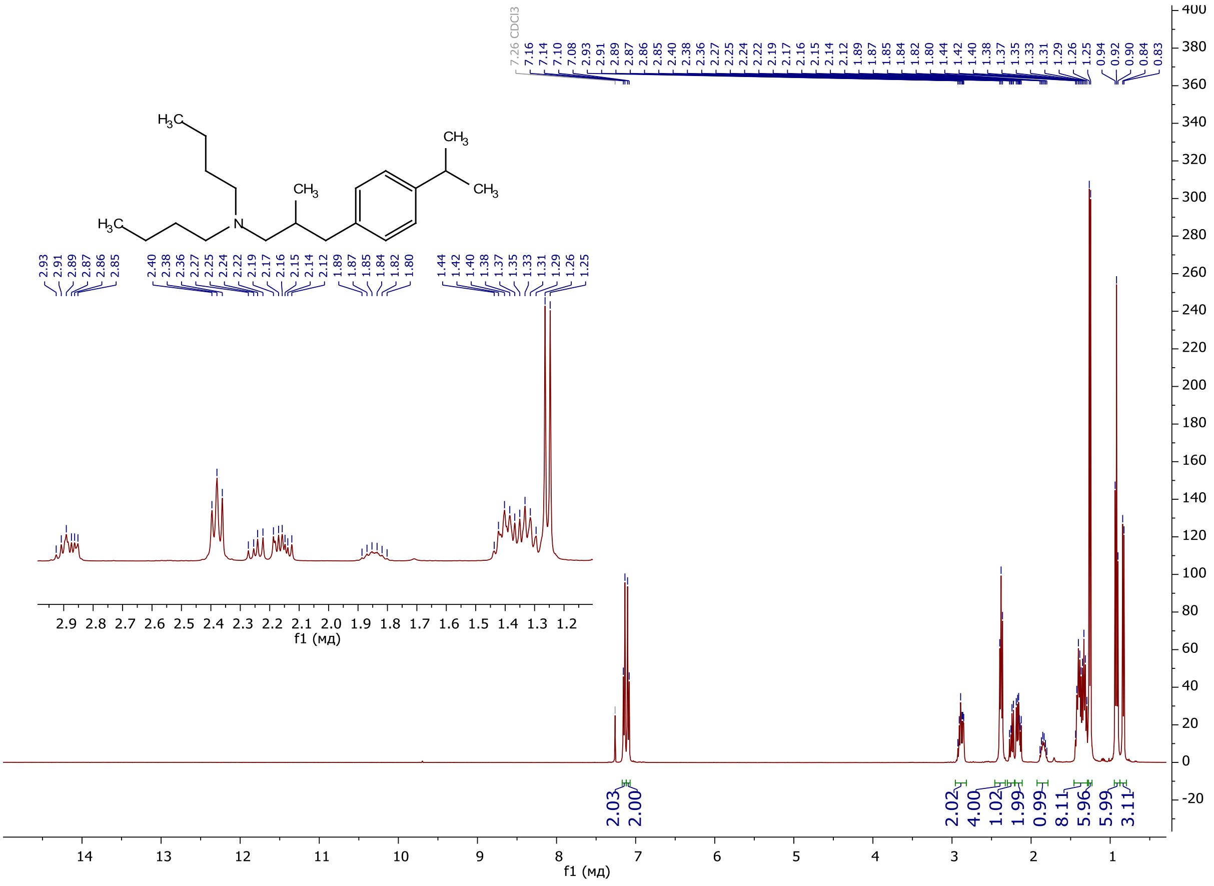

^{13}C spectrum of *N*-butyl-*N*-(3-(4-isopropylphenyl)-2-methylpropyl)butan-1-amine (11) ($CDCl_3$, 101 MHz)

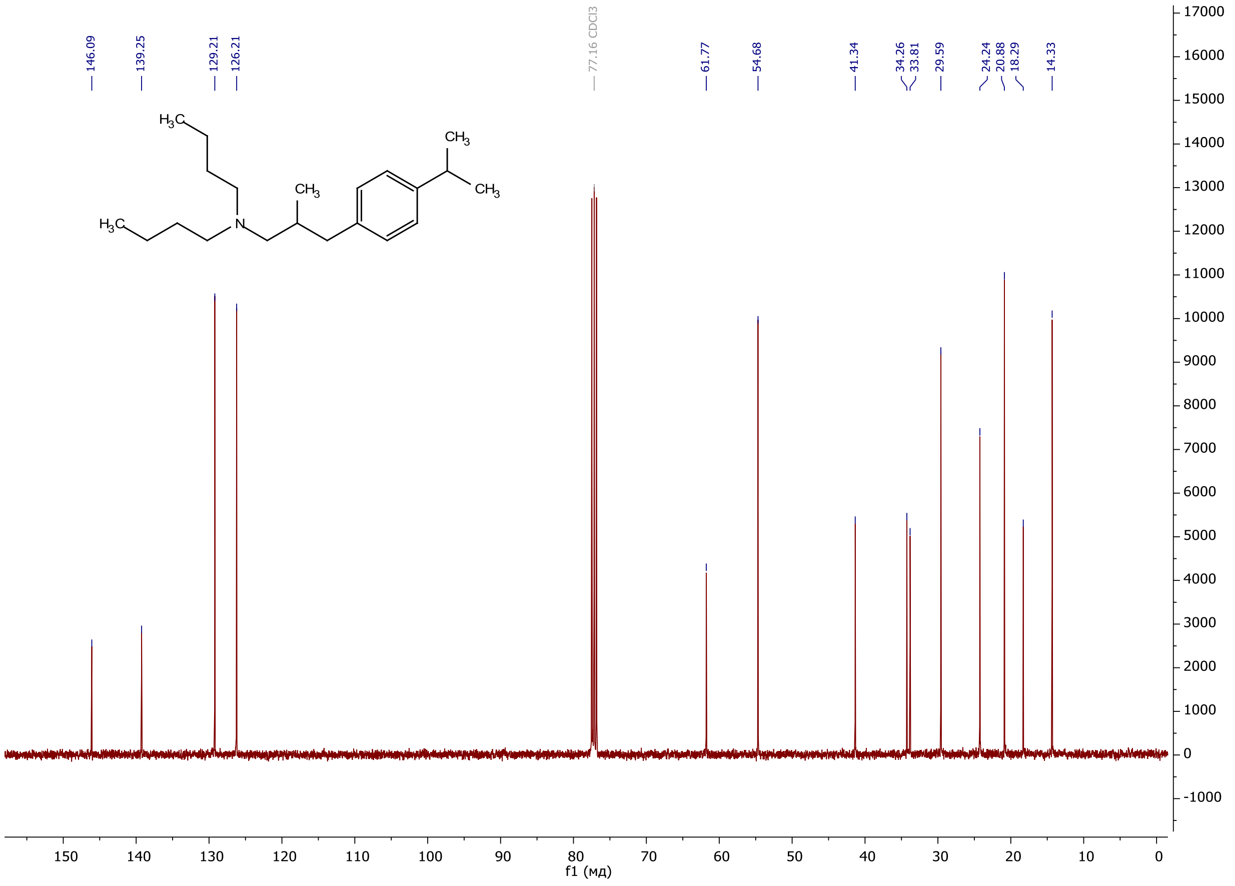

^{1}H spectrum of *N*-allyl-*N*-(2-(4-(tert-butyl)phenyl)propyl)prop-2-en-1-amine (12) ($CDCl_3$, 300 MHz)

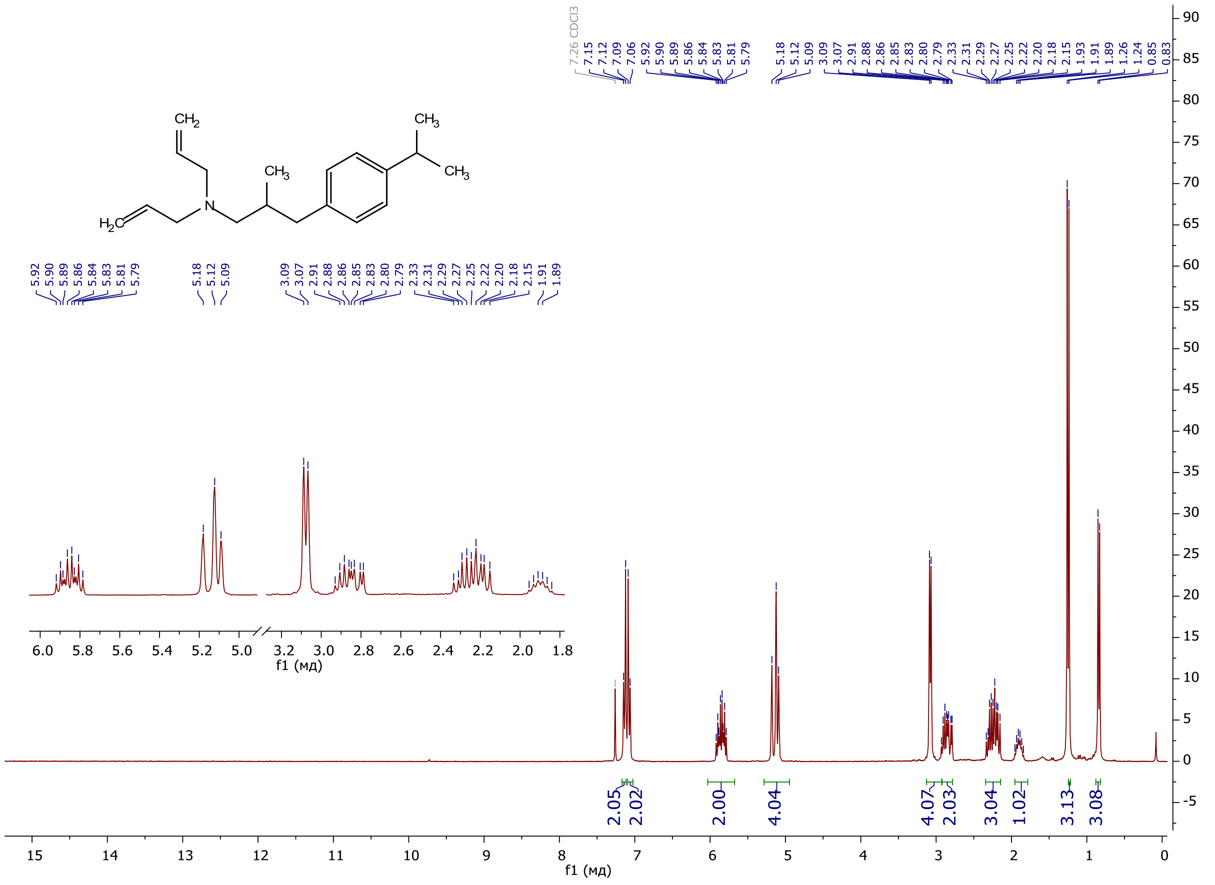

^{13}C spectrum of *N*-allyl-*N*-(2-(4-(tert-butyl)phenyl)propyl)prop-2-en-1-amine (12) ($CDCl_3$, 101 MHz)

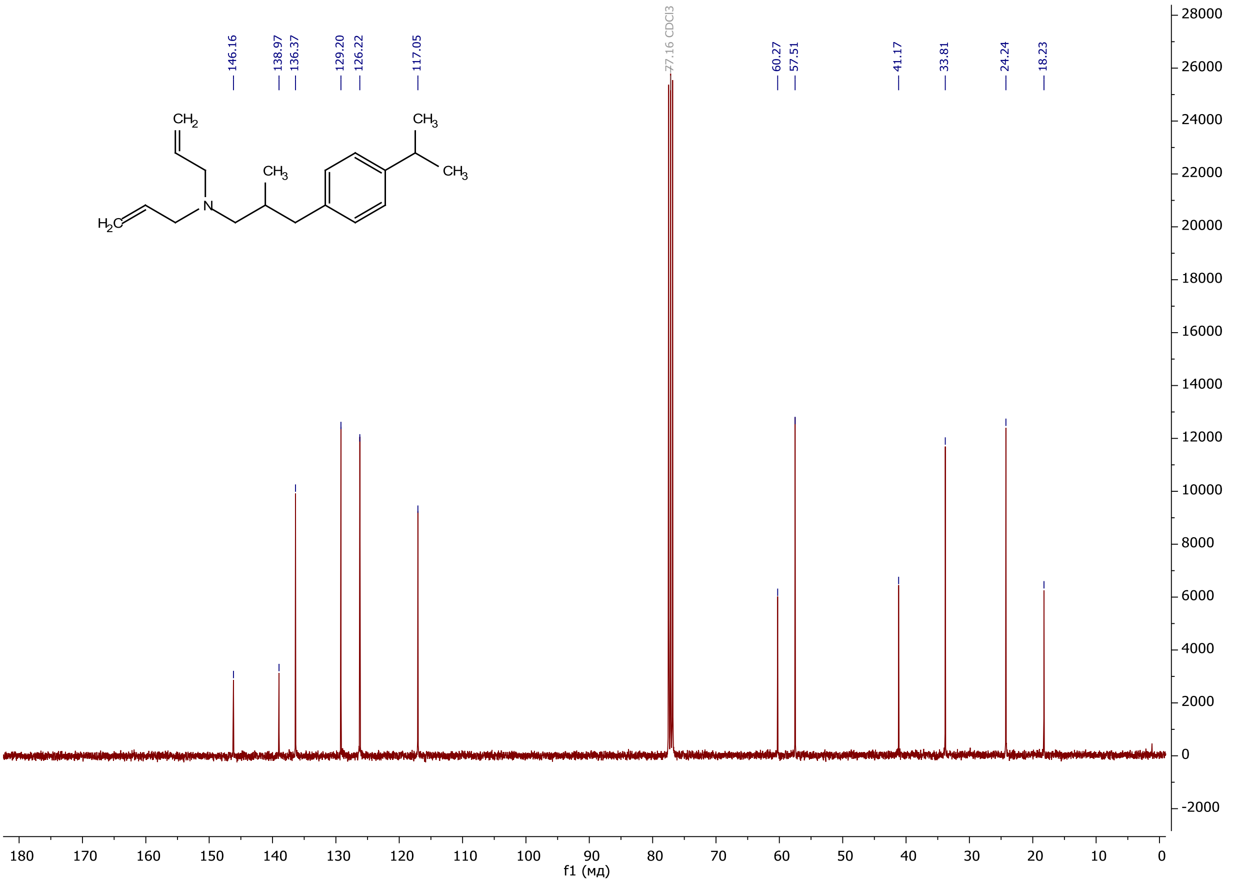

^{1}H spectrum of 4-(2-methoxybenzyl)morpholine (13) ($CDCl_3$, 400 MHz)

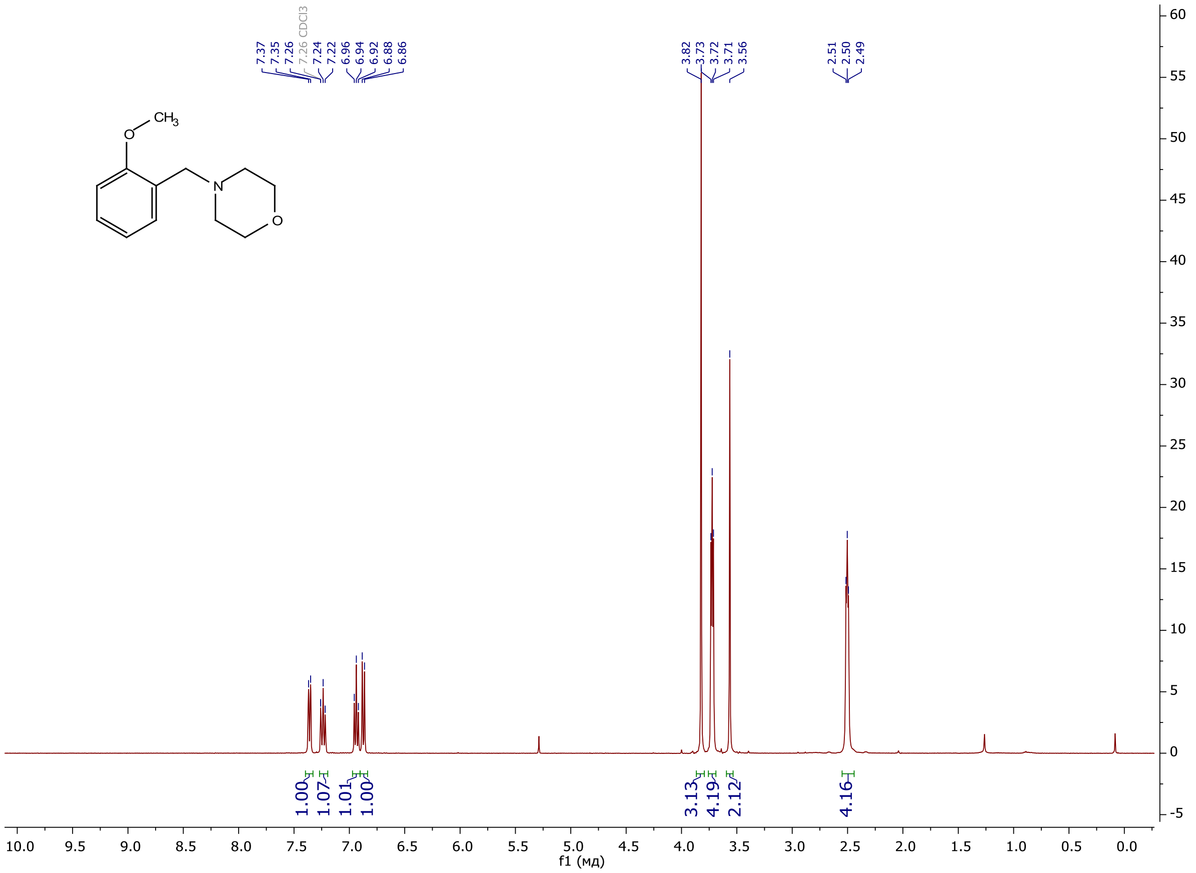

^{13}C spectrum of 4-(2-methoxybenzyl)morpholine (13) ($CDCl_3$, 101 MHz)

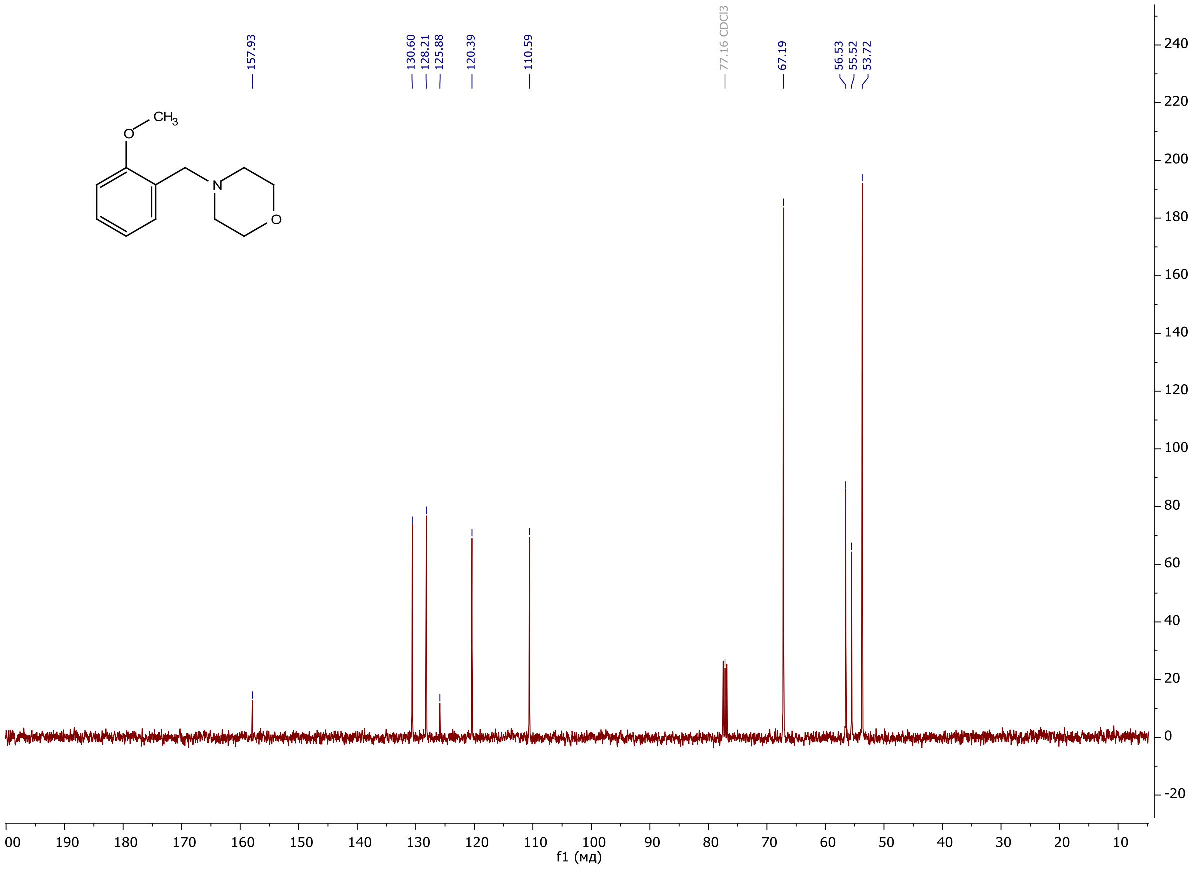

^{1}H spectrum of *N*,*N*-dibenzyl-1-cyclohexylmethanamine (14) ($CDCl_3$, 300 MHz)

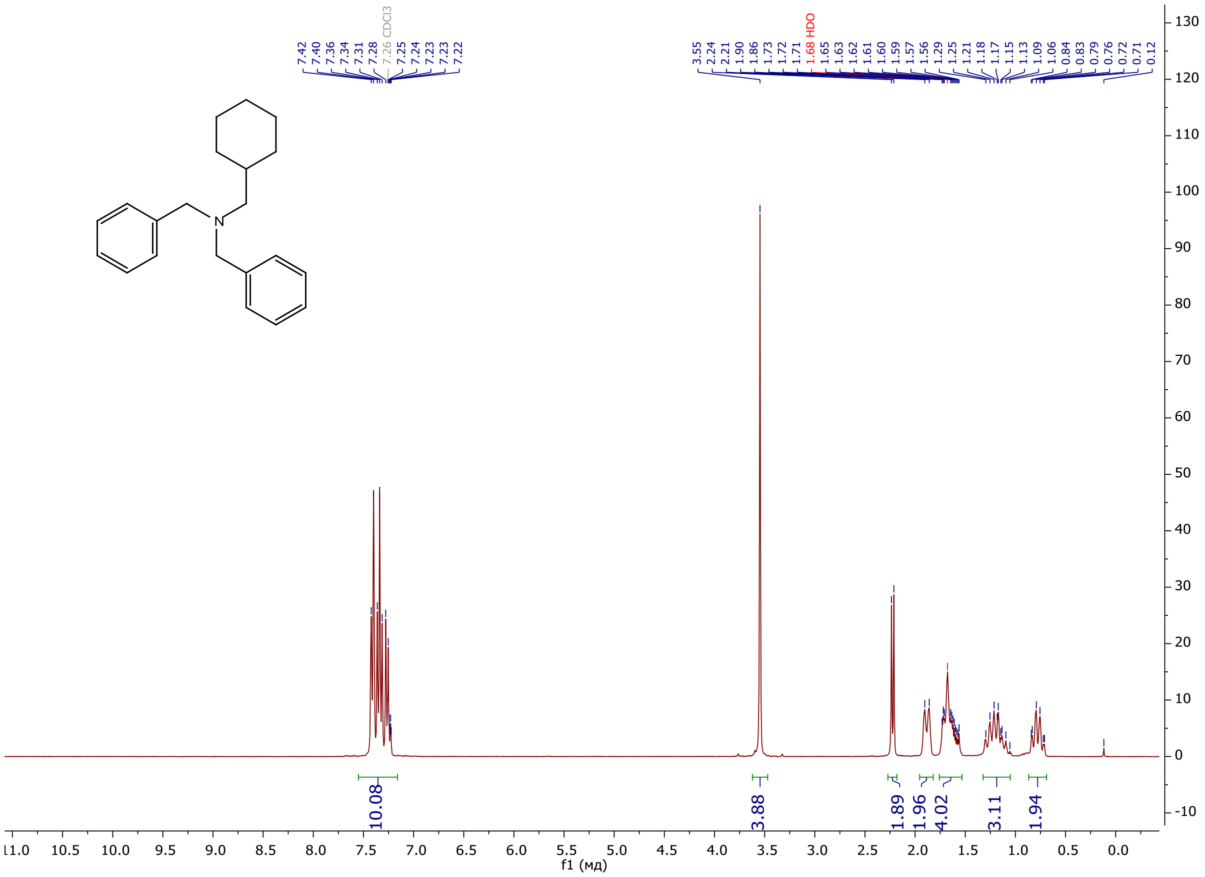

^{13}C spectrum of *N*,*N*-dibenzyl-1-cyclohexylmethanamine (14) ($CDCl_3$, 101 MHz)

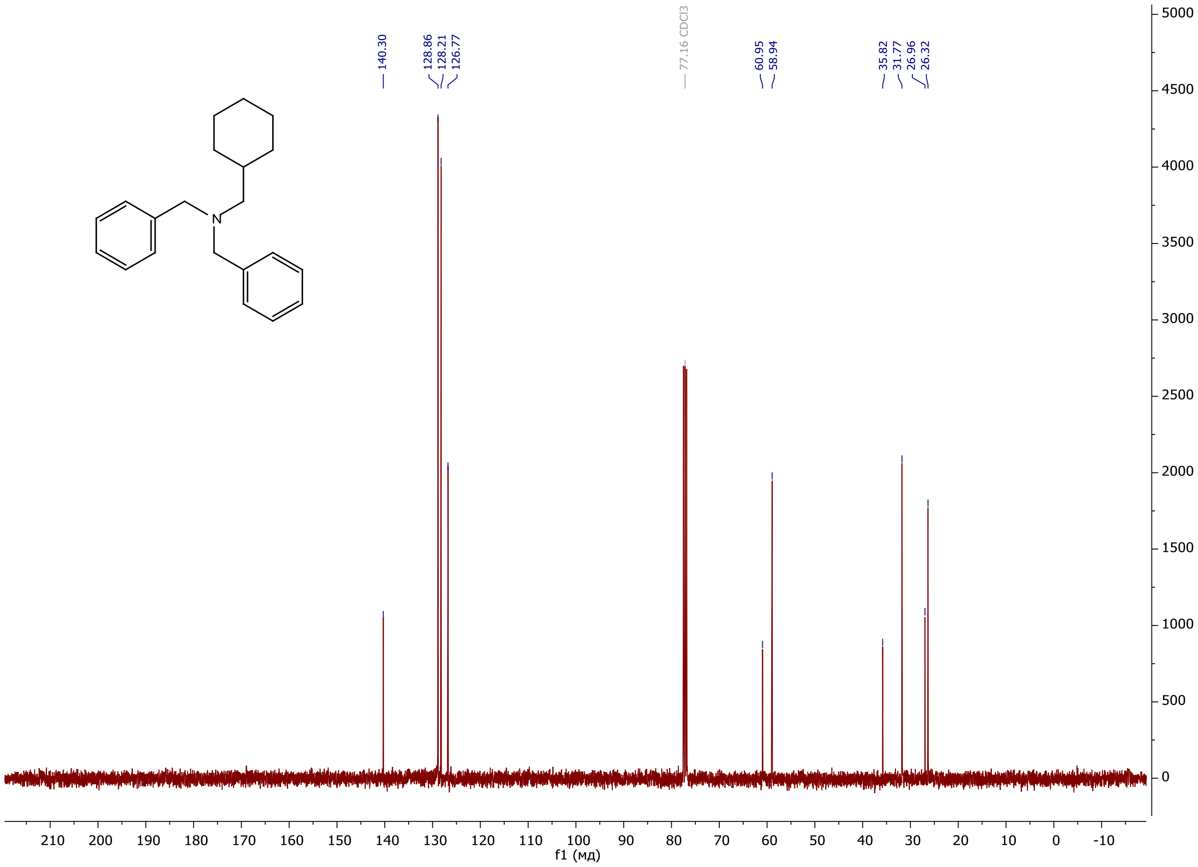

^{1}H spectrum of *N*-(1-phenylethyl)cyclohexanamine (15) ($CDCl_3$, 400 MHz)

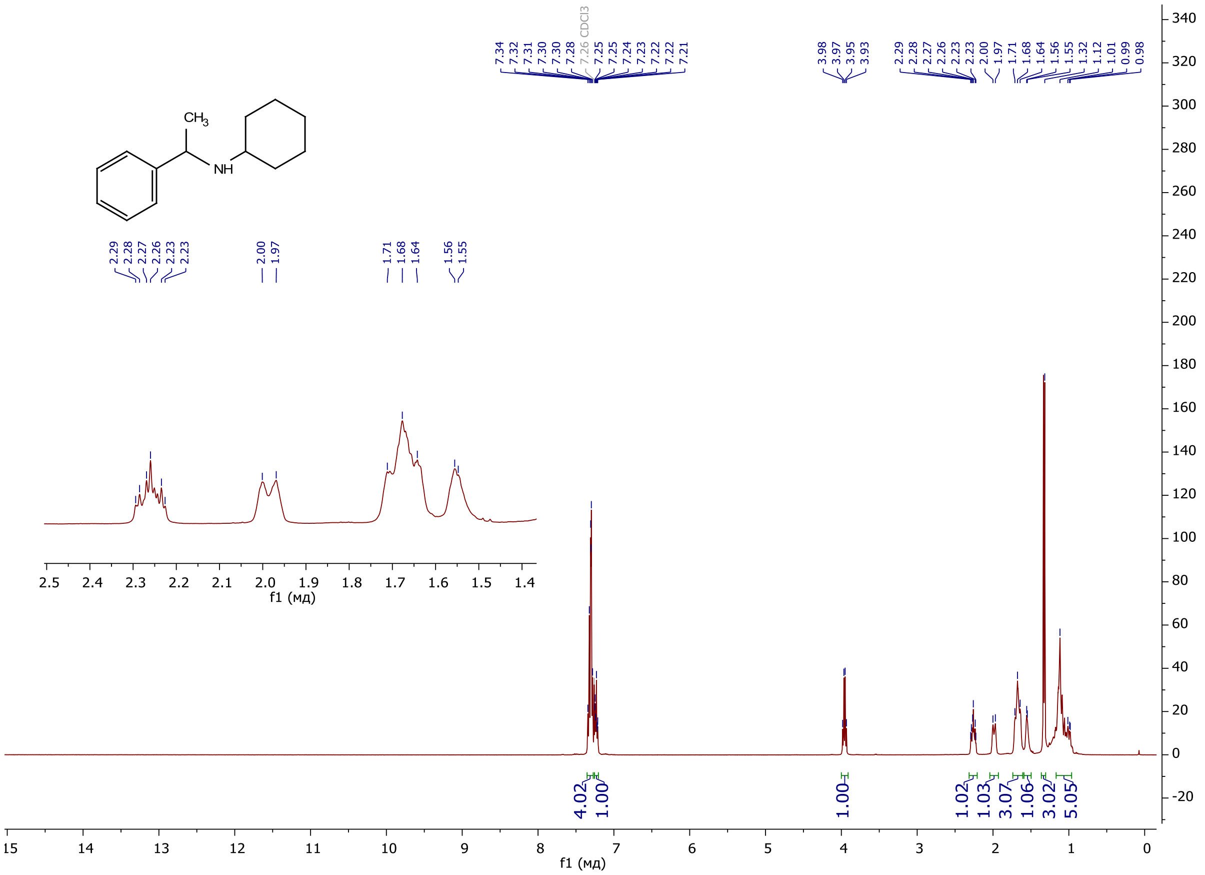

^{13}C spectrum of *N*-(1-phenylethyl)cyclohexanamine (15) ($CDCl_3$, 101 MHz)

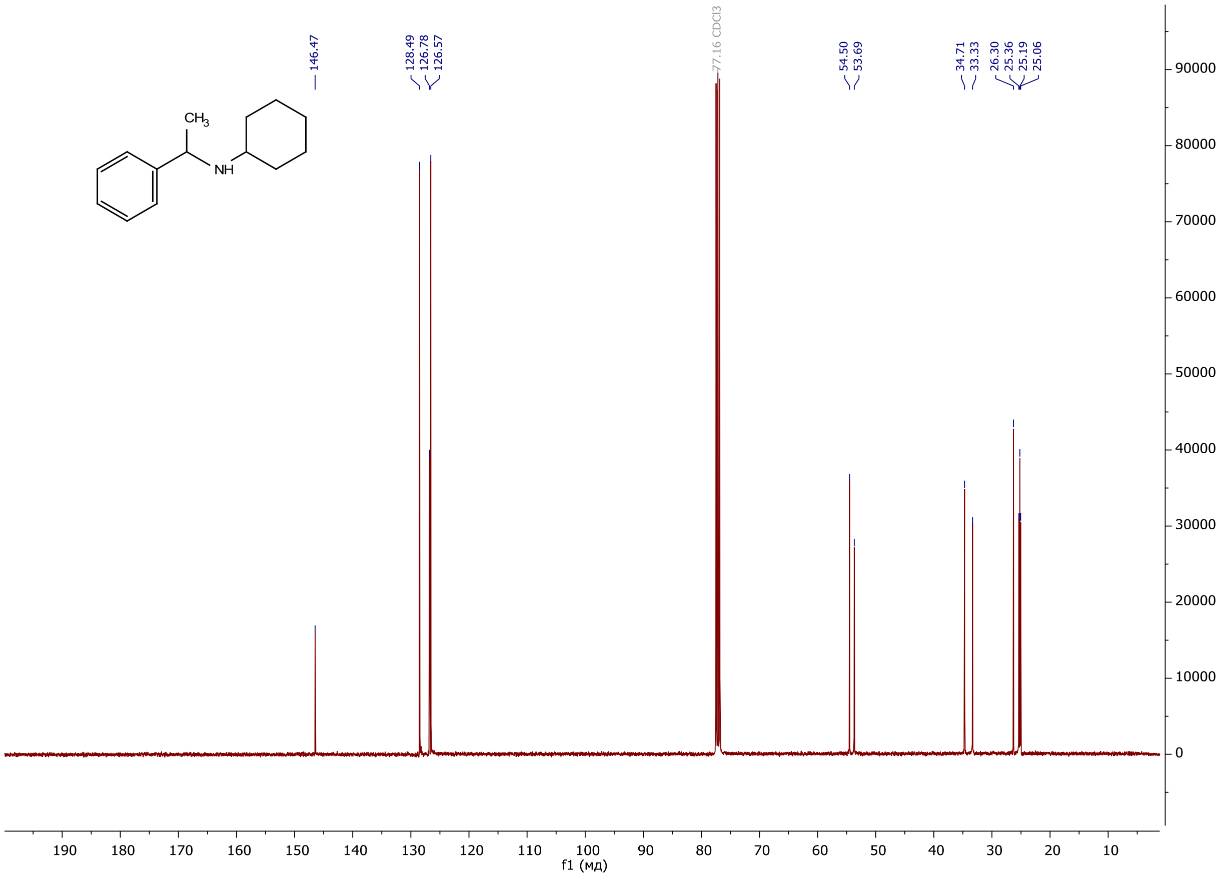

^{1}H spectrum of 4-(3-nitrobenzyl)morpholine (16) ($CDCl_3$, 400 MHz)

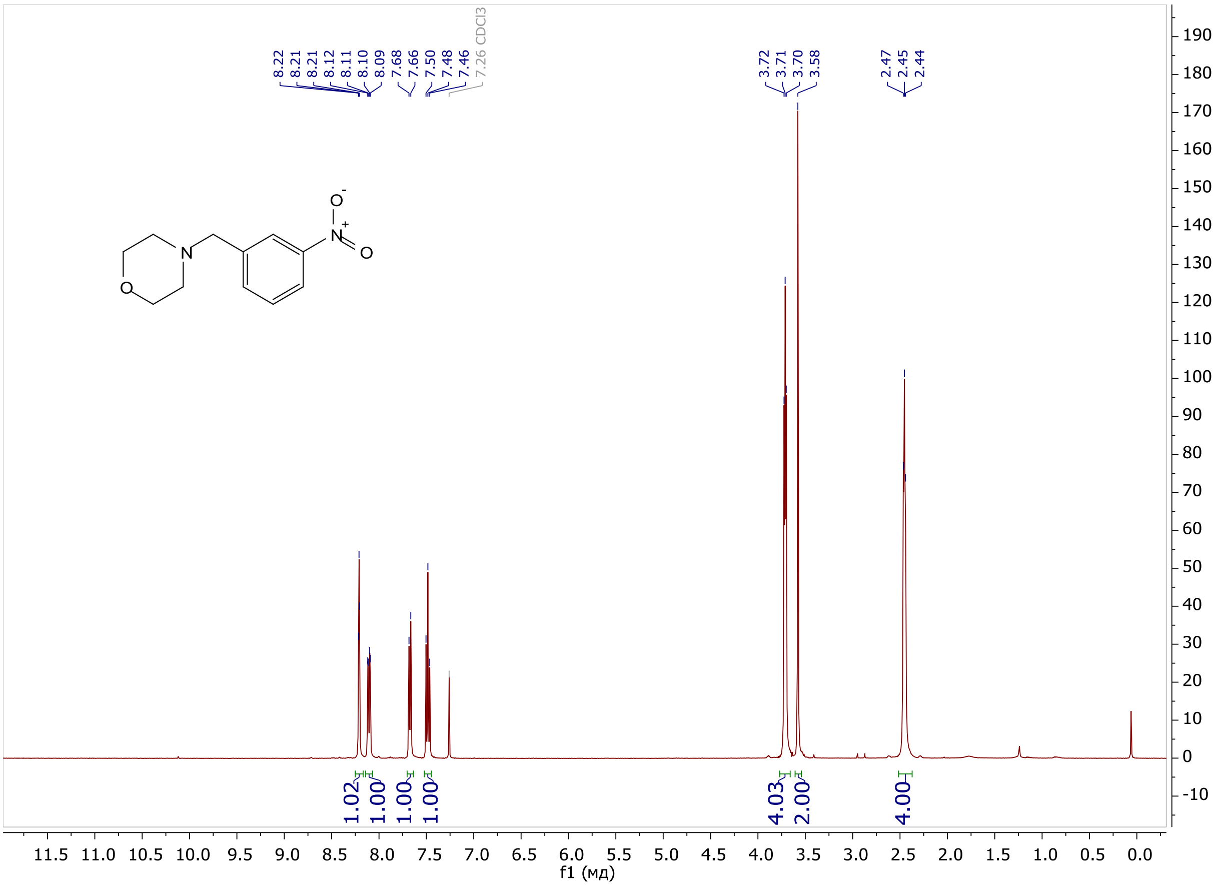

^{13}C spectrum of 4-(3-nitrobenzyl)morpholine (16) ($CDCl_3$, 101 MHz)

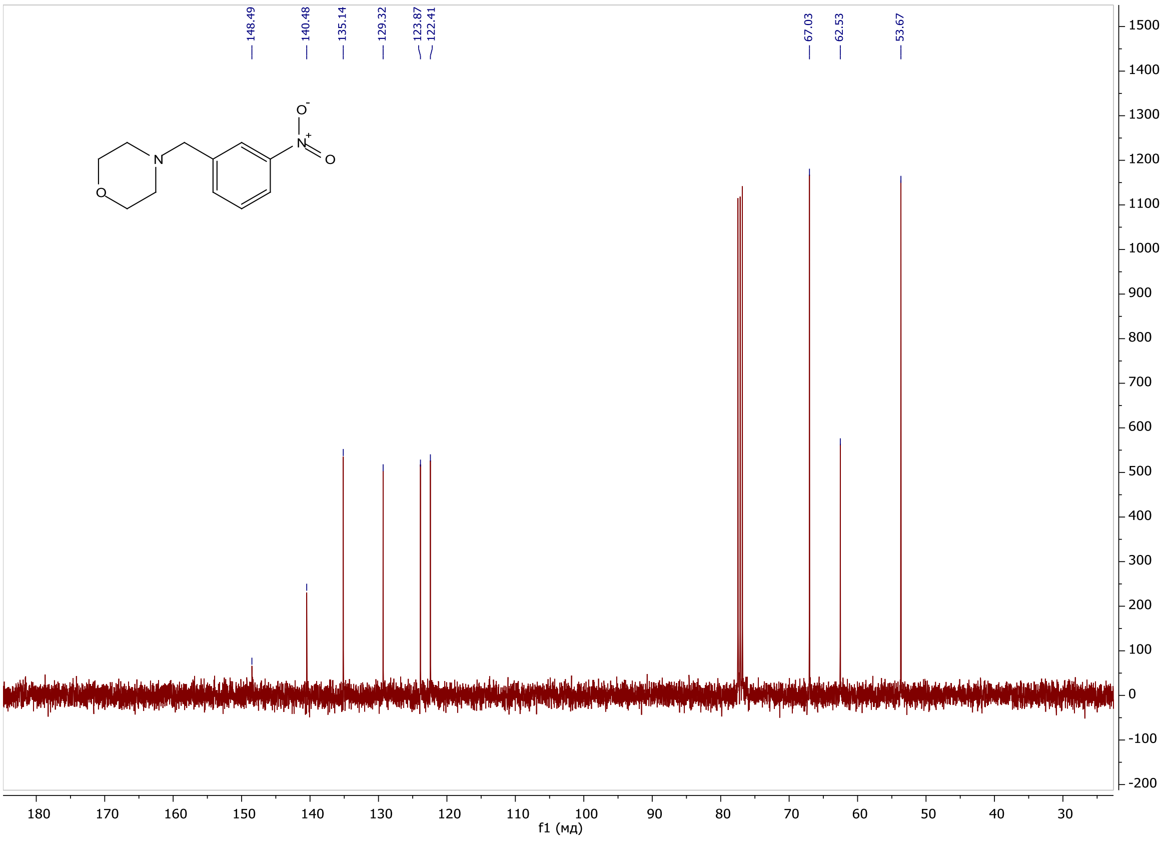

^{1}H spectrum of 4-(4-chlorobenzyl)morpholine (17) ($CDCl_3$, 400 MHz)

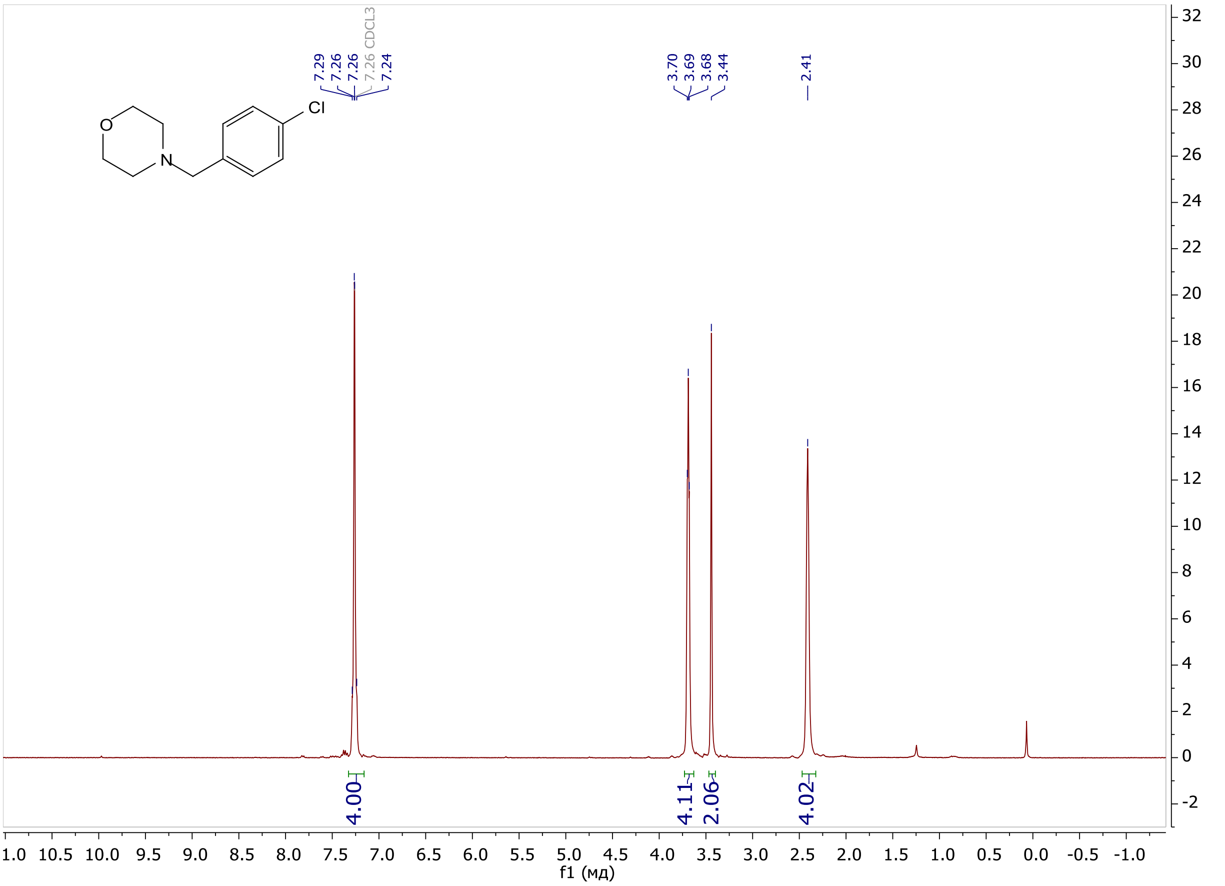

^{13}C spectrum of 4-(4-chlorobenzyl)morpholine (17) ($CDCl_3$, 101 MHz)

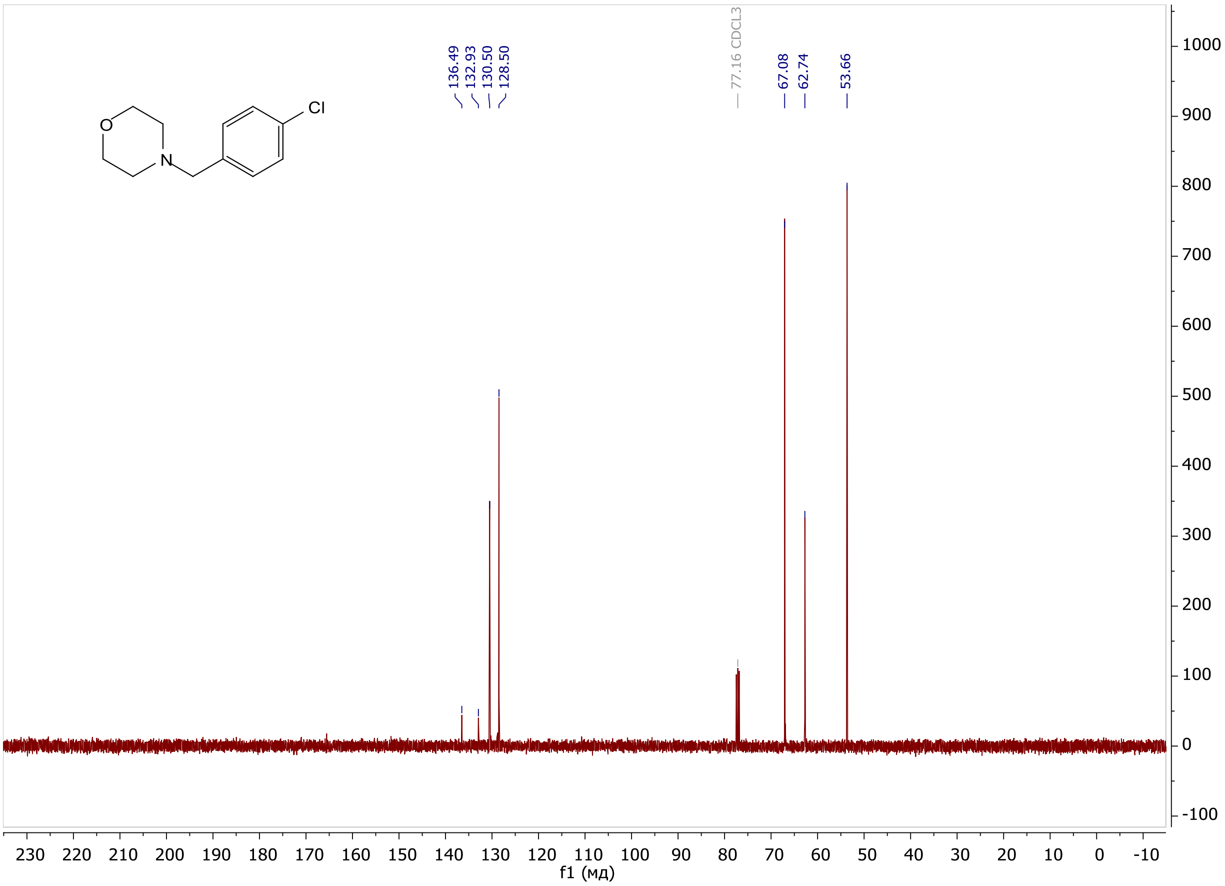

^{1}H spectrum of 4-(3-(trifluoromethyl)benzyl)morpholine (18) ($CDCl_3$, 400 MHz)

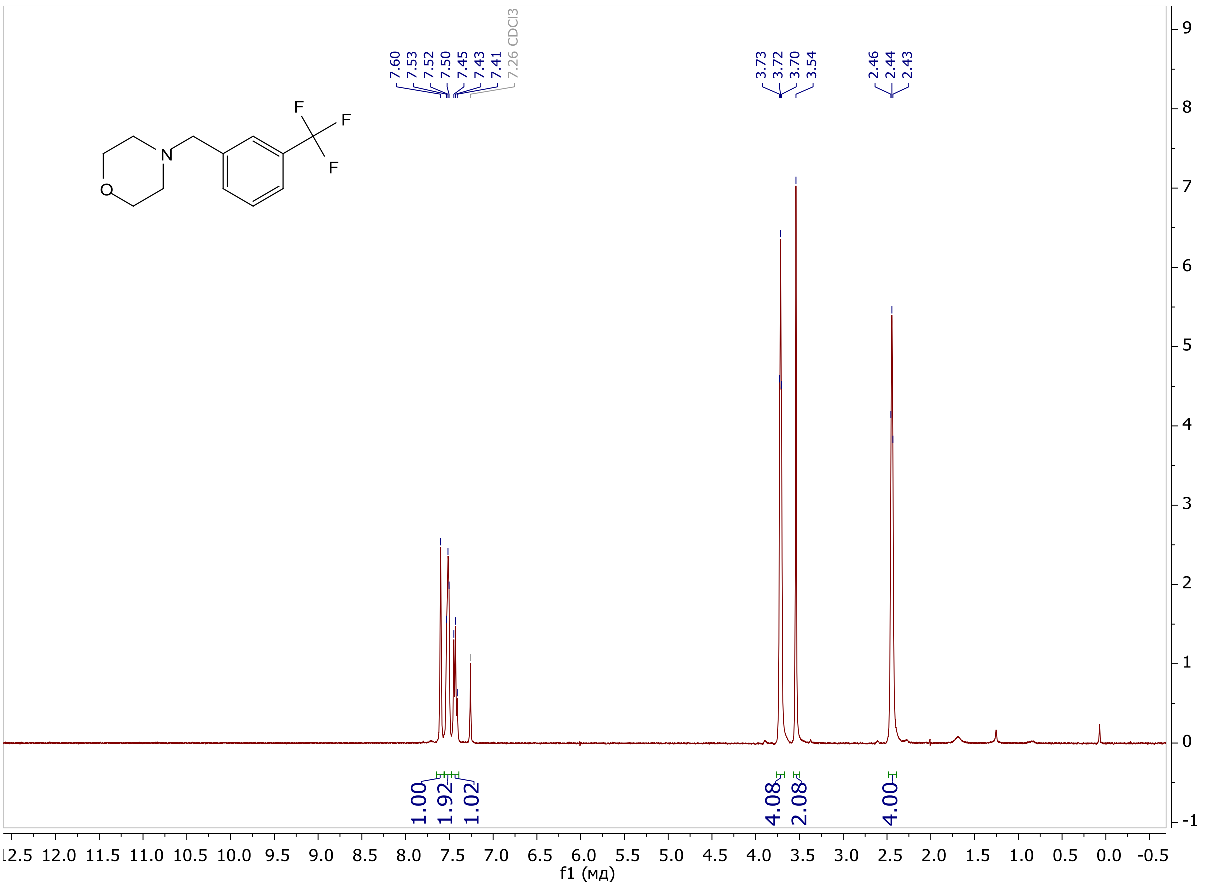

^{13}C spectrum of 4-(3-(trifluoromethyl)benzyl)morpholine (18) ($CDCl_3$, 101 MHz)

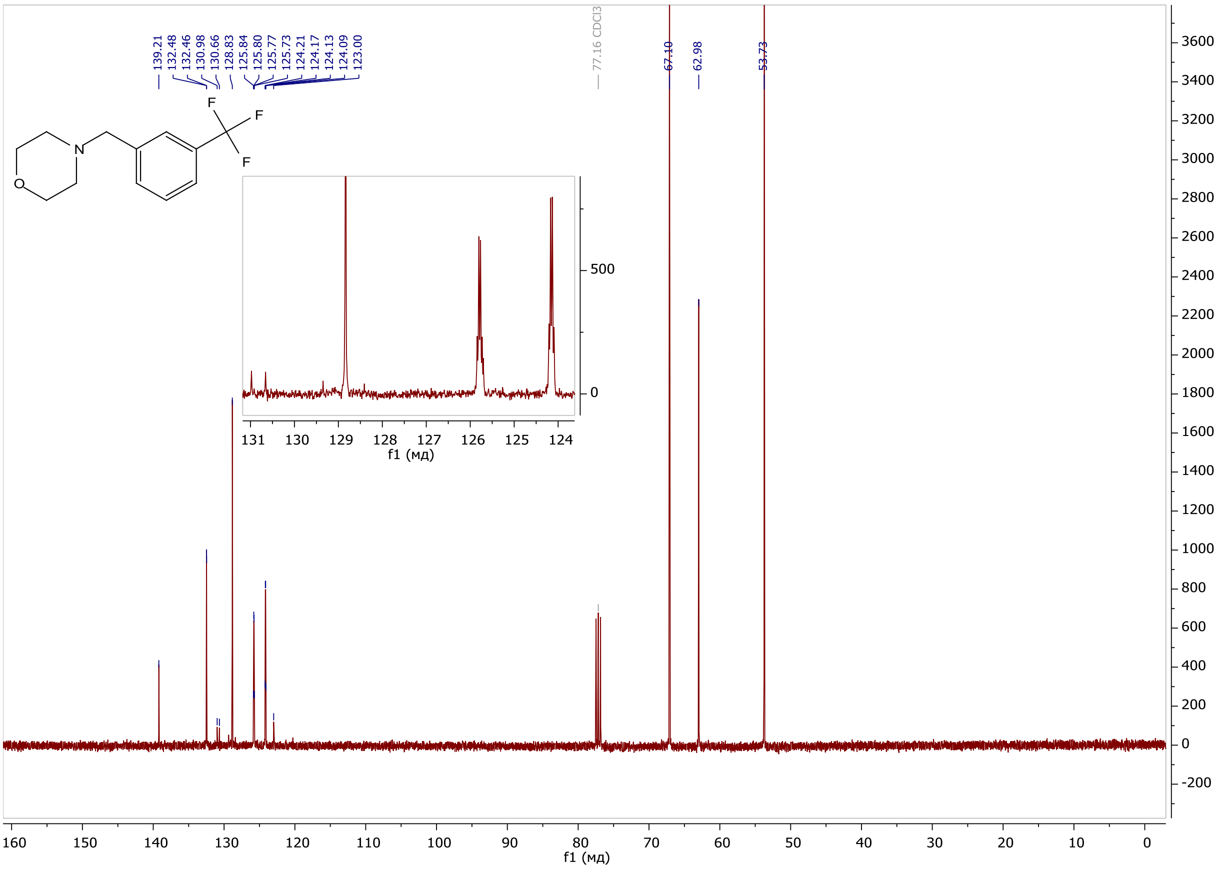

^{19}F spectrum of 4-(3-(trifluoromethyl)benzyl)morpholine (18) ($CDCl_3$, 376 MHz)

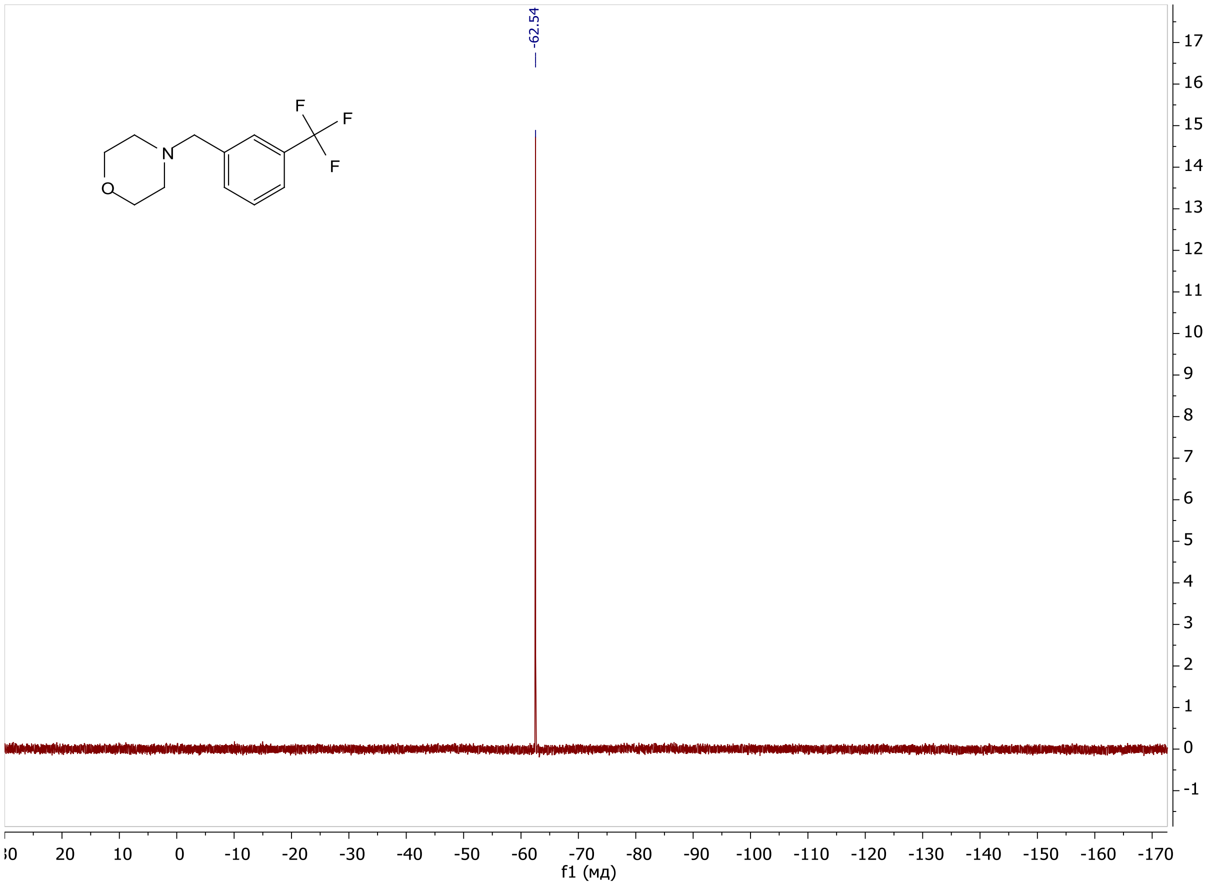

^{1}H spectrum of 4-(morpholinomethyl)benzonitrile (19) ($CDCl_3$, 400 MHz)

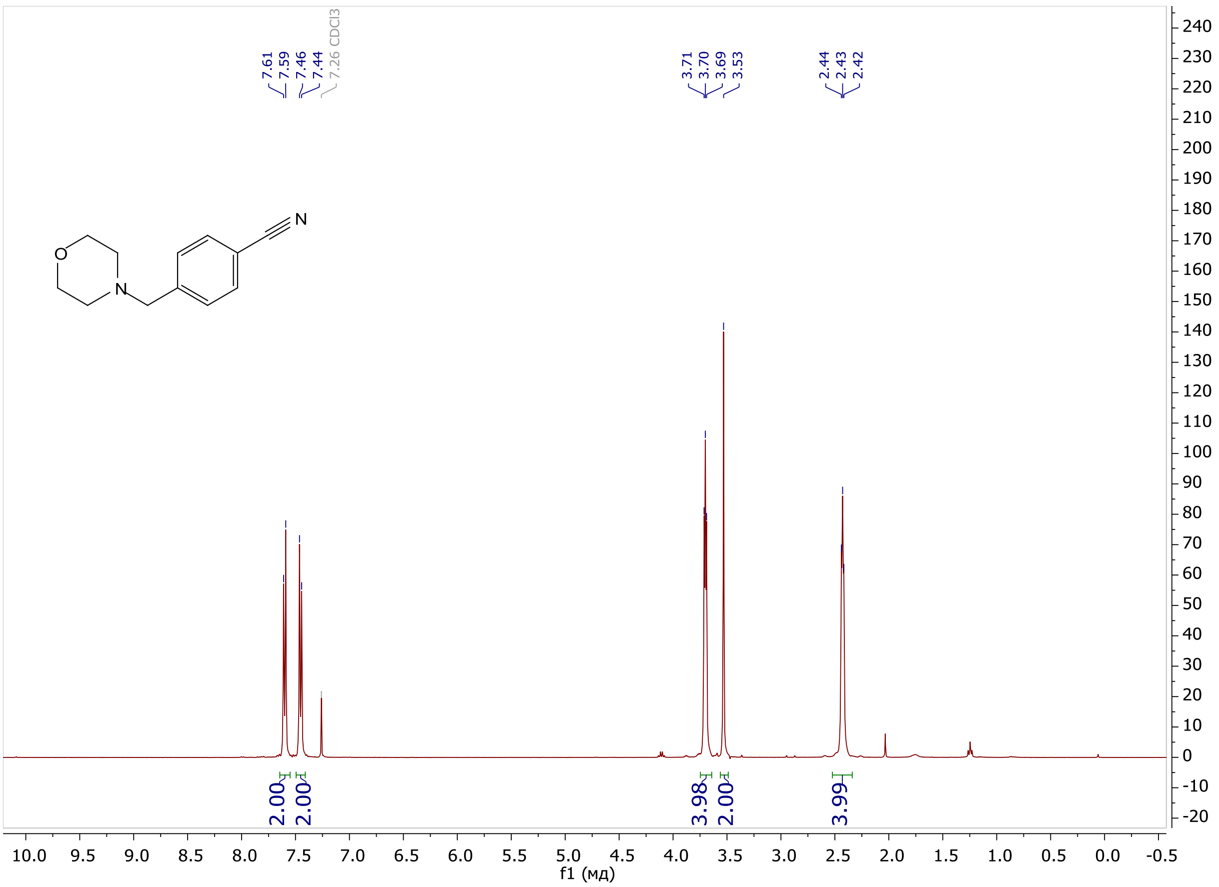

^{13}C spectrum of 4-(morpholinomethyl)benzonitrile (19) ($CDCl_3$, 101 MHz)

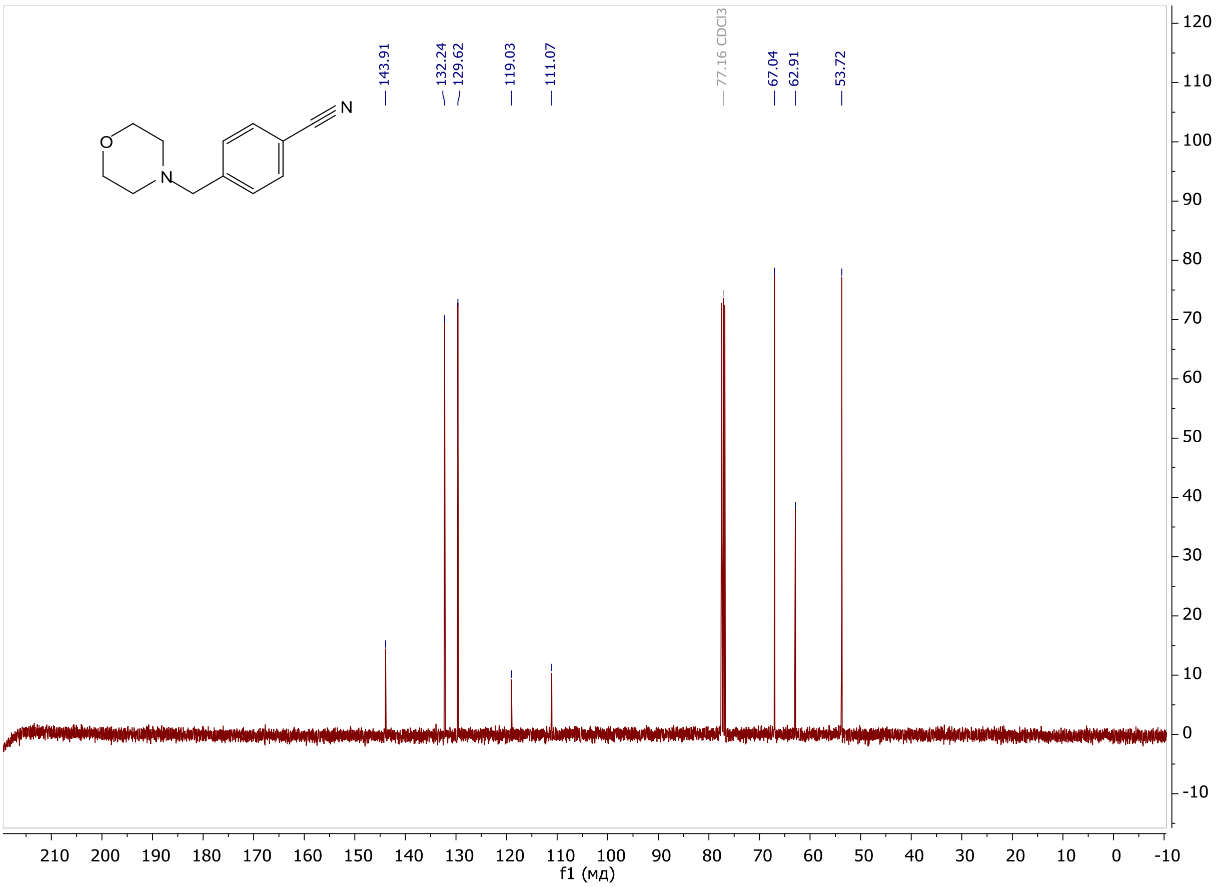

HRMS spectra of the products

HRMS of 4-benzyl-1-(4-methoxybenzyl)piperidine (2)

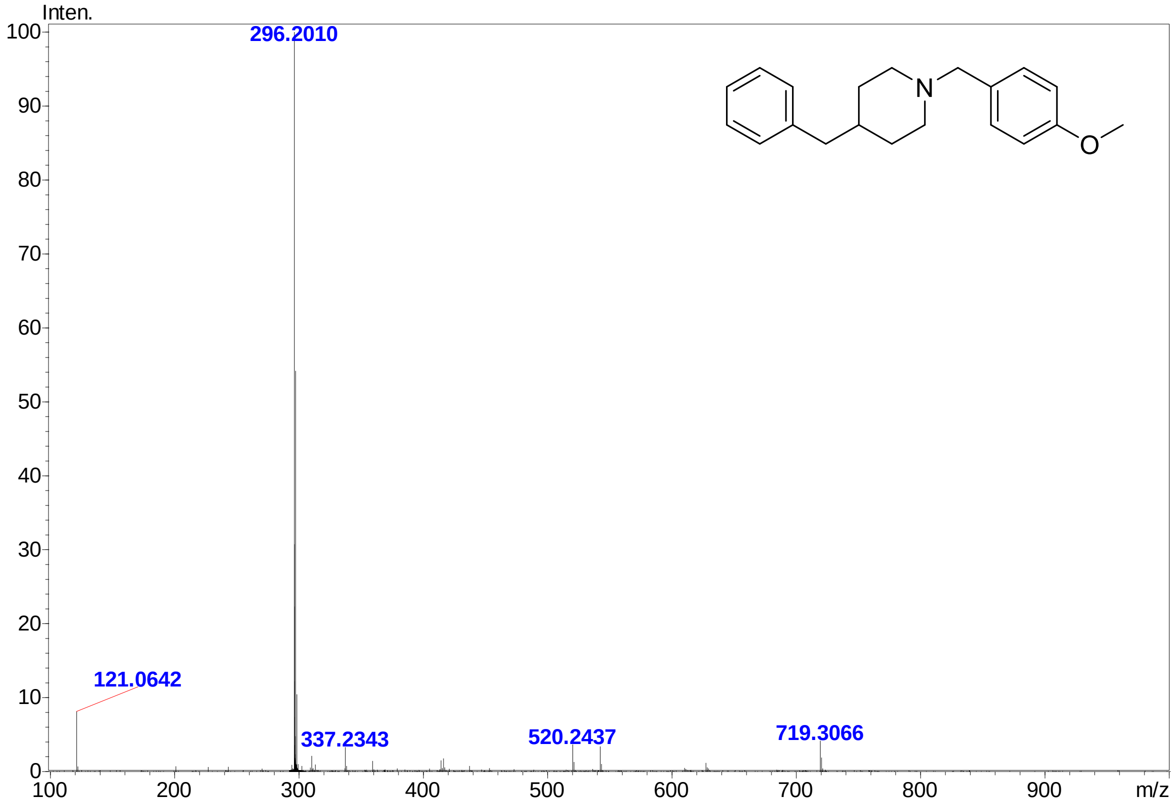

HRMS of ethyl 1-(4-methoxybenzyl)piperidine-4-carboxylate (3)

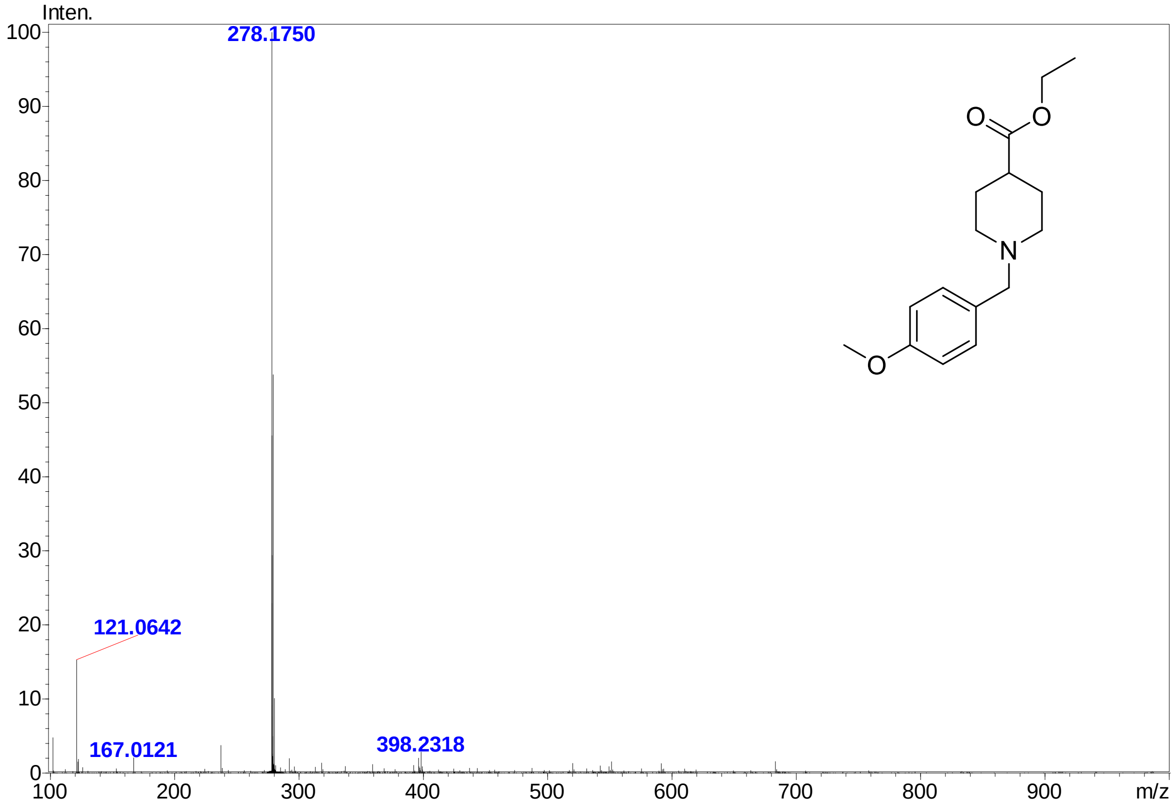

HRMS of *N*-allyl-*N*-(4-methoxybenzyl)prop-2-en-1-amine (5)

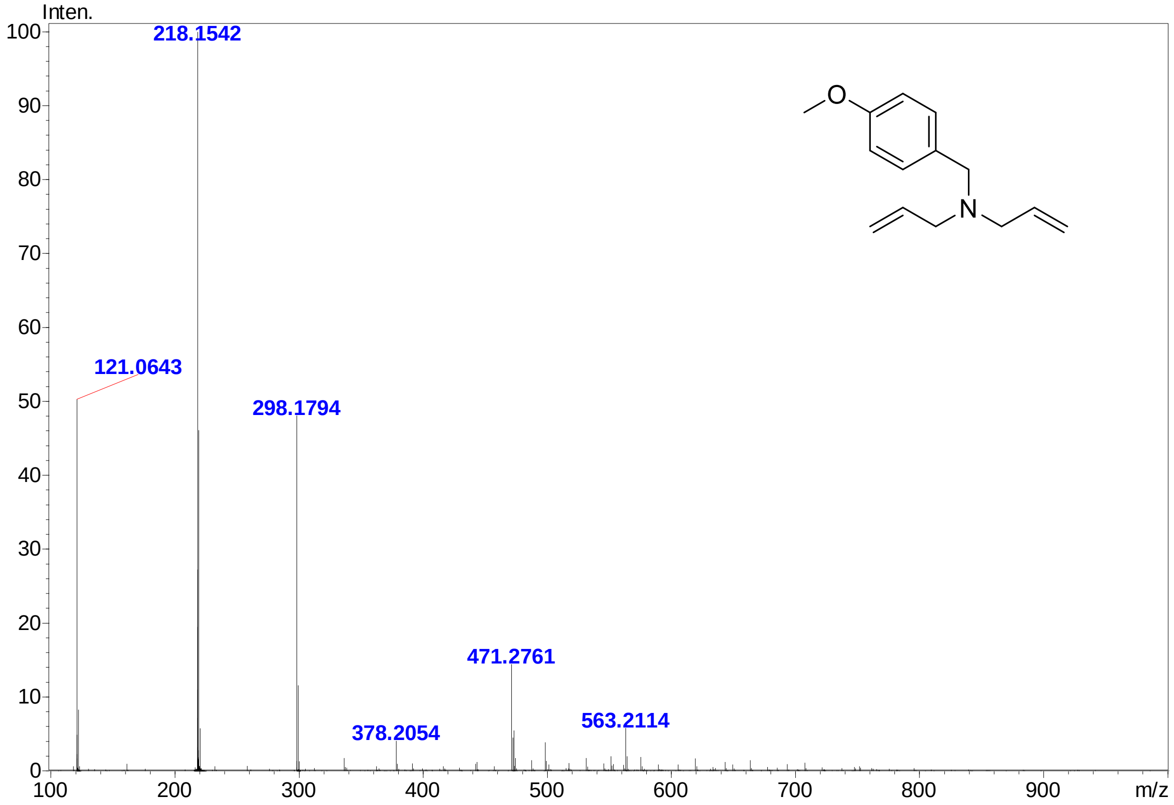

HRMS of *N*1-ethyl-*N*1-(4-methoxybenzyl)-*N*2,*N*2-dimethylethane-1,2-diamine (6)

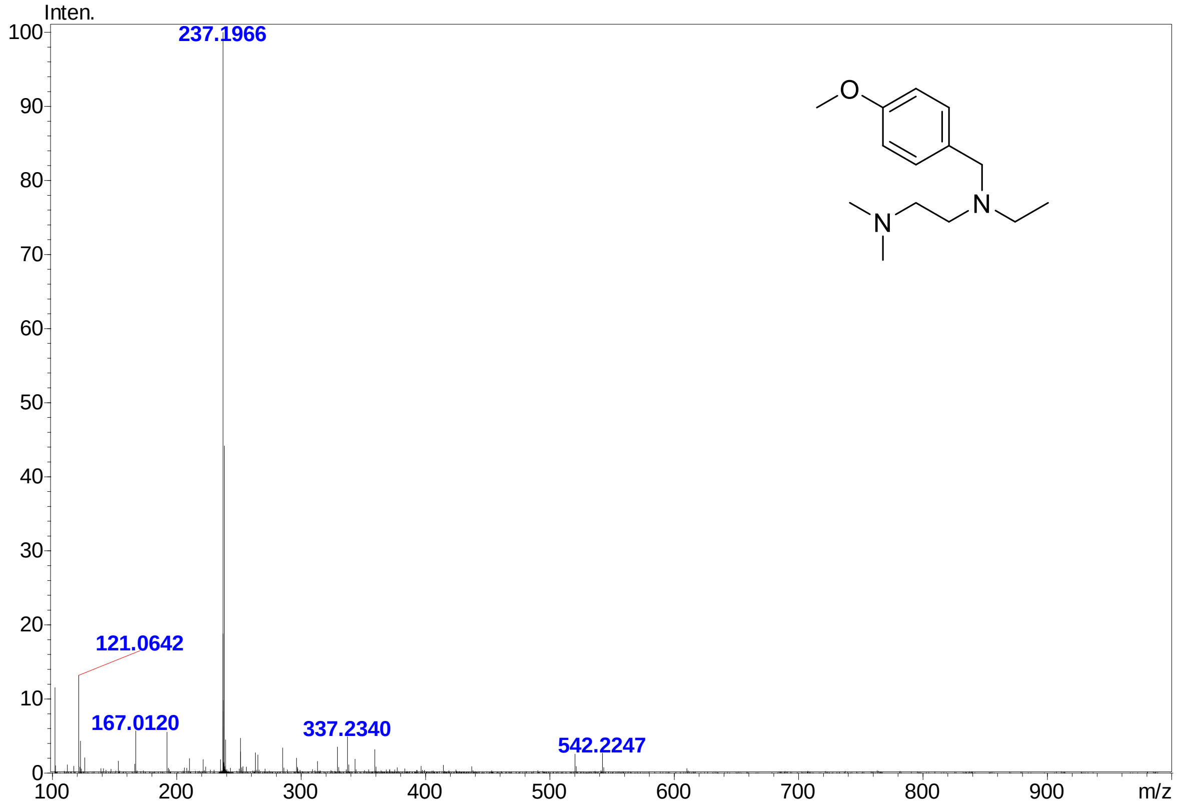

HRMS of 2-(4-(4-methoxybenzyl)piperazin-1-yl)pyrimidine (8)

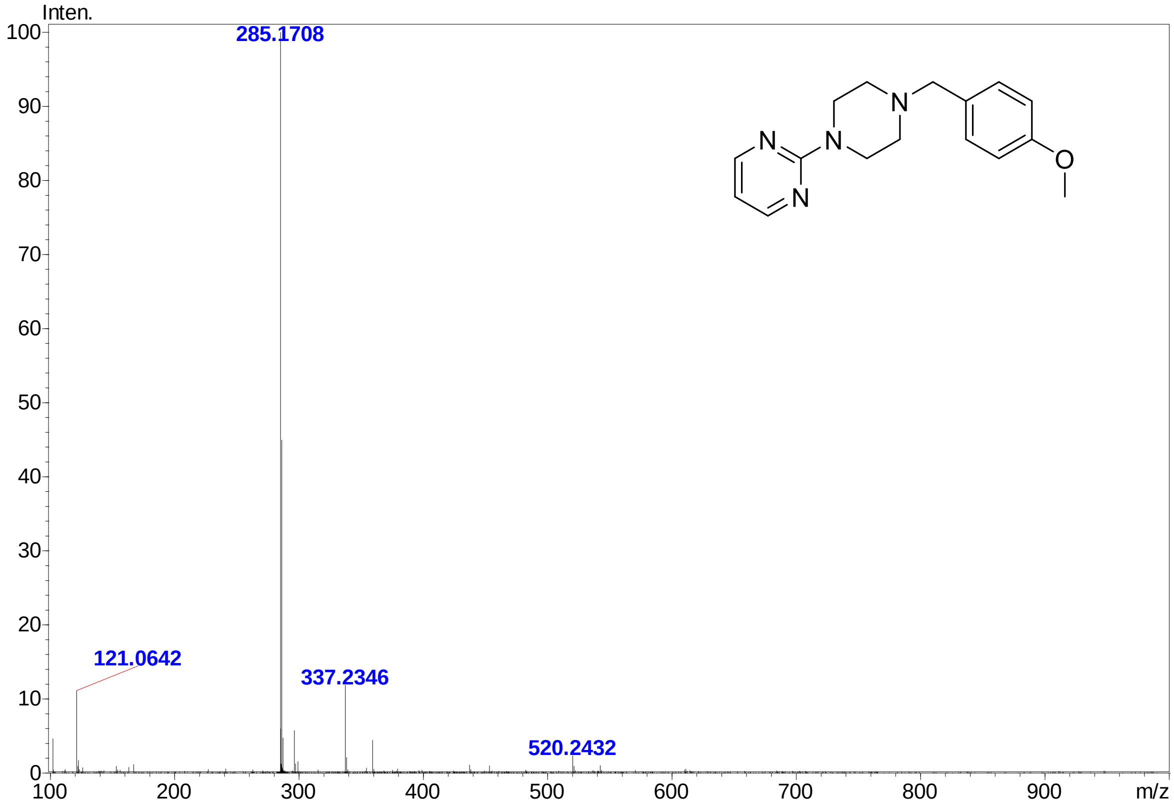

HRMS of *N*-butyl-*N*-(3-(4-isopropylphenyl)-2-methylpropyl)butan-1-amine (11)

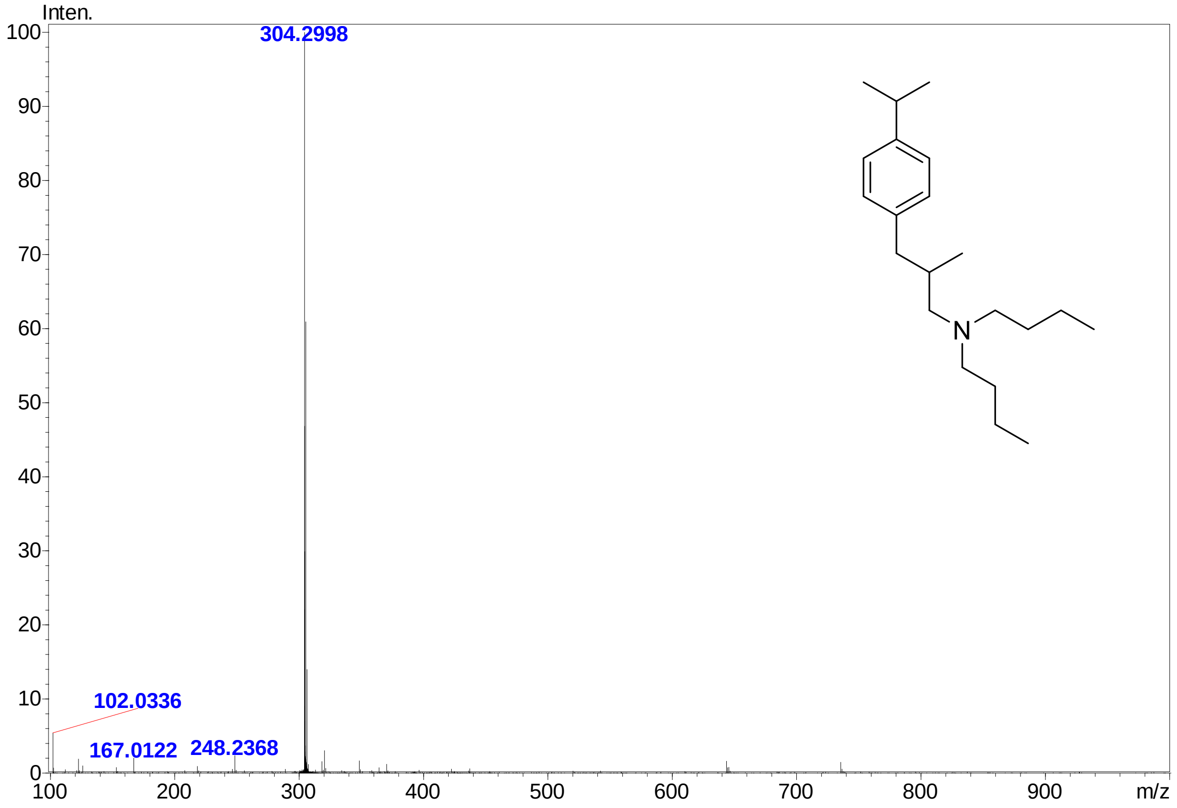

HRMS of *N*-allyl-*N*-(2-(4-(*tert*-butyl)phenyl)propyl)prop-2-en-1-amine (12)

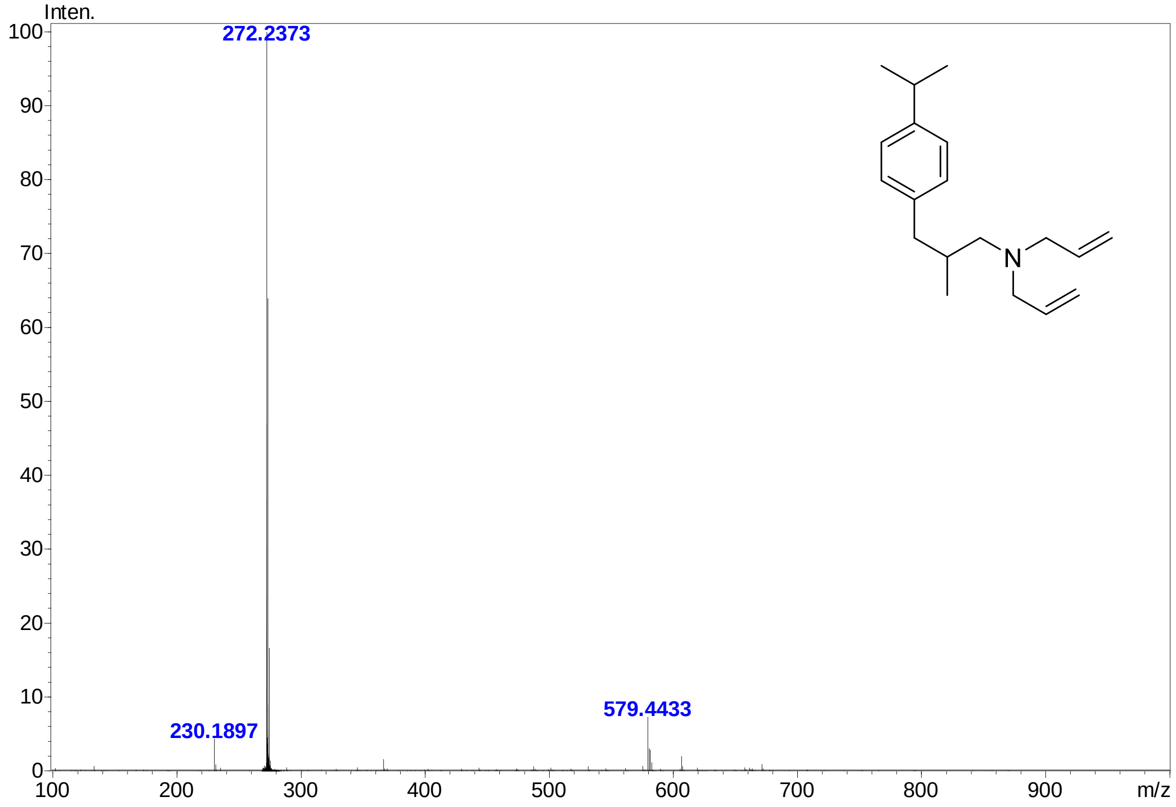

References

(1) Moondra, A.; Mathur, A.; Banerji, K. K. *J. Chem. Soc. Dalton Trans.* **1990**, No. 9, 2697–2700. doi:10.1039/DT9900002697

(2) F. Neese, *WIRES Comput. Molec. Sci.*, **2022**, 12, e1606.

(3) Zhao, Y.; Truhlar, D. G. *Theor. Chem. Acc.* **2008**, *120*, 215–241. doi:10.1007/s00214-007-0310-x

(4) B. P. Pritchard, D. Altarawy, B. Didier, T. D. Gibsom and T. L. Windus, *J. Chem. Inf. Mod.*, 2019, **59**, 4814–4820.

(5) Barone, V.; Cossi, M. *J. Phys. Chem. A* **1998**, *102*, 1995–2001. doi:10.1021/jp9716997

(6) Kliuev, F.; Kuznetsov, A.; Afanasyev, O. I.; Runikhina, S. A.; Kuchuk, E.; Podyacheva, E.; Tsygankov, A. A.; Chusov, D. *Org. Lett.* **2022**, *24*, 7717–7721. doi:10.1021/acs.orglett.2c02807

(7) Enyong, A. B.; Moasser, B. *J. Org. Chem.* **2014**, *79*, 7553–7563. doi:10.1021/jo501273t

(8) Das, B. G.; Ghorai, P. *Chem. Commun.* **2012**, *48*, 8276. doi:10.1039/c2cc33185c

(9) Runikhina, S. A.; Afanasyev, O. I.; Kuchuk, E. A.; Perekalin, D. S.; Jagadeesh, R. V.; Beller, M.; Chusov, D. *Chem. Sci.* **2023**, *14*, 4346–4350. doi:10.1039/D3SC00257H

(10) An, J.; Gao, Z.; Wang, Y.; Zhang, Z.; Zhang, J.; Li, L.; Tang, B.; Wang, F. *Green Chem.* **2021**, *23*, 2722–2728. doi:10.1039/D1GC00113B

(11) Luo, Z.; Wan, S.; Pan, Y.; Yao, Z.; Zhang, X.; Li, B.; Li, J.; Xu, L.; Fan, Q. *Asian J. Org. Chem.* **2022**, *11*, e202100707. doi:10.1002/ajoc.202100707

(12) Blessley, G.; Holden, P.; Walker, M.; Brown, J. M.; Gouverneur, V. *Org Lett* **2012**, *14*, 2754–2757. doi:10.1021/ol300977f

(13) Le Gall, E.; Decompte, A.; Martens, T.; Troupel, M. *Synthesis* **2010**, *2010*, 249–254. doi:10.1055/s-0029-1217108

(14) Li, Q.; Liskey, C. W.; Hartwig, J. F. *J. Am. Chem. Soc.* **2014**, *136*, 8755–8765. doi:10.1021/ja503676d
